# Supplementary material for: RNA sequencing-based exploration of the effects of blue laser irradiation on mRNAs involved in functional metabolites of D. officinales
Source: PeerJ. 2022 Jan 4;10:e12684. doi: 10.7717/peerj.12684 (PMC8740519; doi:10.7717/peerj.12684)
Supplement: Supplemental Information 1 [file peerj-10-12684-s001.zip › Supplemental information/Table S1.docx]

| **Table S1** Primers information used for real-time PCR analysis of *D. officinale* genes | | | |
| --- | --- | --- | --- |
| **Gene name** | **Primer sequences（5’→3’）** | **Size（bp）** | **TM（°C）** |
| HSP70-F | AACTCTTTCCTCCACTGCCC | 166 | 62 |
| HSP70-R | TGTCCATCTTCGCATCTCTCA |  |  |
| CYP86A4S-F | CTTTCCAGTCGCCTAAATGCT | 161 | 62 |
| CYP86A4S-R | ATCTCGTCAAACTCCTCCGC |  |  |
| ERF013-F | GCTGATGCCTTTGAATGTATGG | 100 | 62 |
| ERF013-R | GAAGAGGGCTGATTTAGGGC |  |  |
| CRY DASH-F | AACAGTGATTGAAACGGCTG | 136 | 62 |
| CRY DASH-R | GCTGAATGATGTTGCTTCGT |  |  |
| SPA1-F | TCAGAACCAACTTGAACTGCC | 175 | 62 |
| SPA1-R | GCCACTTTGTGAATCCTTCG |  |  |
| HY5-F | CAACCCTGCGACTCACTCAT | 163 | 62 |
| HY5-R | CTTGCGTTTGAAGCCGTAAT |  |  |
| CO16-F | GTGGCAGAACAAGTTGATGCTA | 131 | 62 |
| CO16-R | AGCGTTTGCTGGATTGGAAT |  |  |
| MYC2-F | GATTCCTCATCGGGTGGTTC | 184 | 62 |
| MYC2-R | CACTACAGGACCTCATTACGGC |  |  |
| PIF4-F | AACTCTTTCCTCCACTGCCC | 127 | 62 |
| PIF4-R | TGTCCATCTTCGCATCTCTCA |  |  |
| actin-F | AGCCATACTGTCCCAATCTACG | 114 | 62 |
| actin-R | AGCCACGCTCGGTAAGAATC |  |  |
