# Supplementary material for: RNA sequencing-based exploration of the effects of blue laser irradiation on mRNAs involved in functional metabolites of D. officinales
Source: PeerJ. 2022 Jan 4;10:e12684. doi: 10.7717/peerj.12684 (PMC8740519; doi:10.7717/peerj.12684)
Supplement: Supplemental Information 1 [file peerj-10-12684-s001.zip › Supplemental information/Table S10.docx]

| **Table S10** Anthocyanin contents of leaves in *D. officinale* under different light treatments | | | | | | | | |  |
| --- | --- | --- | --- | --- | --- | --- | --- | --- | --- |
| Light treatments | Light intensity (µmol·m^-2^·s^-1^) | Photoperiod (h) | Anthocyanin contents 1  (mg·g ^-1^DW) | Anthocyanin contents 2  (mg·g ^-1^ DW) | Anthocyanin contents 3  (mg·g ^-1^ DW) | Average anthocyanin  contents  (mg·g ^-1^ DW) | Standard deviation | Duncan (5%) | Duncan (1%) |
| White | 100 | 12 | 15.322 | 14.809 | 15.094 | 15.07 | 0.210 | c | C |
| Blue | 100 | 12 | 16.119 | 16.973 | 17.372 | 16.82 | 0.523 | b | B |
| Blue Laser | 100 | 12 | 20.448 | 20.676 | 20.847 | 20.66 | 0.163 | a | A |
