# Supplementary material for: RNA sequencing-based exploration of the effects of blue laser irradiation on mRNAs involved in functional metabolites of D. officinales
Source: PeerJ. 2022 Jan 4;10:e12684. doi: 10.7717/peerj.12684 (PMC8740519; doi:10.7717/peerj.12684)
Supplement: Supplemental Information 1 [file peerj-10-12684-s001.zip › Supplemental information/Table S12.docx]

| **Table S12** polysaccharide contents of leaves in *D. officinale* under different light treatments | | | | | | | | |  |
| --- | --- | --- | --- | --- | --- | --- | --- | --- | --- |
| Light treatments | Light intensity (µmol·m^-2^·s^-1^) | Photoperiod (h) | polysaccharide contents 1  (mg·g ^-1^DW) | polysaccharide contents 2  (mg·g ^-1^ DW) | polysaccharide contents 3  (mg·g ^-1^ DW) | Average polysaccharide  contents  (mg·g ^-1^ DW) | Standard deviation | Duncan (5%) | Duncan (1%) |
| White | 100 | 12 | 154.26 | 148.67 | 151.06 | 151.33 | 2.81 | c | C |
| Blue | 100 | 12 | 176.36 | 171.03 | 169.17 | 172.19 | 3.73 | b | B |
| Blue Laser | 100 | 12 | 189.40 | 181.95 | 187.81 | 186.39 | 3.92 | a | A |
