# Supplementary material for: RNA sequencing-based exploration of the effects of blue laser irradiation on mRNAs involved in functional metabolites of D. officinales
Source: PeerJ. 2022 Jan 4;10:e12684. doi: 10.7717/peerj.12684 (PMC8740519; doi:10.7717/peerj.12684)
Supplement: Supplemental Information 1 [file peerj-10-12684-s001.zip › Supplemental information/Table S14.docx]

| **Table S14** Alkaloid contents of leaves in *D. officinale* under different light treatments | | | | | | | | |  |
| --- | --- | --- | --- | --- | --- | --- | --- | --- | --- |
| Light treatments | Light intensity (µmol·m^-2^·s^-1^) | Photoperiod (h) | Alkaloid  contents 1  (mg·g ^-1^DW) | Alkaloid  contents 2  (mg·g ^-1^ DW) | Alkaloid  contents 3  (mg·g ^-1^ DW) | Average alkaloid  contents  (mg·g ^-1^ DW) | Standard deviation | Duncan (5%) | Duncan (1%) |
| White | 100 | 12 | 51.86 | 50.10 | 49.32 | 50.43 | 1.061 | b | B |
| Blue | 100 | 12 | 72.16 | 74.11 | 71.38 | 72.55 | 1.149 | a | A |
| Blue Laser | 100 | 12 | 72.45 | 74.60 | 72.06 | 73.04 | 1.116 | a | A |
