# Supplementary material for: RNA sequencing-based exploration of the effects of blue laser irradiation on mRNAs involved in functional metabolites of D. officinales
Source: PeerJ. 2022 Jan 4;10:e12684. doi: 10.7717/peerj.12684 (PMC8740519; doi:10.7717/peerj.12684)
Supplement: Supplemental Information 1 [file peerj-10-12684-s001.zip › Supplemental information/Table S16.pdf]

Table S16 The Ct mean of DEGs in *D. officinale* under different light treatments

Light treatment	*HSP* 70 Ct Mean	*CYP* 86*A* 4*S* Ct Mean	*ERF* 13 Ct Mean	*CRY DASH* Ct Mean	*SPA* 1 Ct Mean	*HY* 5 Ct Mean	*CO* 16 Ct Mean	*MYC* 2 Ct Mean	*PIF* 4 Ct Mean	*Actin* Ct Mean
White-1	24.825	24.989	25.979	37.342	29.298	26.193	24.640	23.639	24.930	25.094
White-2	24.987	25.163	26.181	37.833	29.030	25.819	24.771	23.460	24.867	25.046
White-3	24.789	25.044	25.759	37.476	28.882	25.992	24.672	23.609	24.988	25.035
Blue-1	24.845	24.833	25.343	35.963	29.298	26.193	25.040	24.859	25.135	25.094
Blue-2	24.949	24.765	25.328	35.768	29.030	25.819	25.171	24.739	25.157	25.046
Blue-3	24.885	24.707	25.476	35.955	28.882	25.992	25.072	24.918	25.183	25.067
Blue laser-1	23.575	24.635	24.349	34.867	28.639	25.452	25.405	25.445	24.373	25.110
Blue laser-2	23.830	24.851	24.412	34.641	28.209	25.739	25.440	25.385	24.773	25.397
Blue laser-3	23.867	24.673	24.221	34.976	28.365	26.217	25.410	25.304	24.749	25.122
