# Supplementary material for: RNA sequencing-based exploration of the effects of blue laser irradiation on mRNAs involved in functional metabolites of D. officinales
Source: PeerJ. 2022 Jan 4;10:e12684. doi: 10.7717/peerj.12684 (PMC8740519; doi:10.7717/peerj.12684)
Supplement: Supplemental Information 1 [file peerj-10-12684-s001.zip › Supplemental information/Table S17.pdf]

Table S17 The relative expression of DEGS in *D. officinale* under different light treatments

Light treatment	*HSP* 70		*CYP* 86*A* 4*S*		*ERF* 13		*CRY DASH*		*SPA* 1		*HY* 5		*CO* 16		*MYC* 2		*PIF* 4	
	Relative expression	SD	Relative expression	SD	Relative expression	SD	Relative expression	SD	Relative expression	SD	Relative expression	SD	Relative expression	SD	Relative expression	SD	Relative expression	SD
White	1.000	0.106	1.000	0.089	1.000	0.211	1.000	0.254	1.000	0.211	1.000	0.187	1.000	0.068	1.000	0.096	1.000	0.061
Blue	0.990	0.053	1.237	0.063	1.517	0.082	3.172	0.111	1.007	0.211	1.007	0.187	0.763	0.068	0.418	0.091	0.859	0.024
Blue laser	2.397	0.159	1.411	0.115	3.475	0.097	7.331	0.171	1.762	0.218	1.275	0.387	0.673	0.019	0.317	0.070	1.364	0.224
