# Supplementary material for: RNA sequencing-based exploration of the effects of blue laser irradiation on mRNAs involved in functional metabolites of D. officinales
Source: PeerJ. 2022 Jan 4;10:e12684. doi: 10.7717/peerj.12684 (PMC8740519; doi:10.7717/peerj.12684)
Supplement: Supplemental Information 1 [file peerj-10-12684-s001.zip › Supplemental information/Table S2.docx]

**Table S2** Phenotype of *D. officinale* after different light treatments

| **Treatment** | **CK** | | | **B** | | | **BL** | | |
| --- | --- | --- | --- | --- | --- | --- | --- | --- | --- |
|  | Average value | Standard deviation | Duncan (5%) | Average value | Standard deviation | Duncan (5%) | Average value | Standard deviation | Duncan (5%) |
| **Plant height**  **(mm)** | 48.05 | 1.08 | a | 48.07 | 1.71 | a | 48.36 | 0.82 | a |
| **Stem diameter (mm)** | 2.41 | 0.03 | a | 2.47 | 0.11 | a | 2.42 | 0.08 | a |
| **Leaf area**  **(mm^2^)** | 104.51 | 0.61 | a | 105.00 | 1.24 | a | 105.71 | 0.77 | a |
| **Number of leaves** | 1 | — | — | 1 | — | — | 3 | — | — |
