# Supplementary material for: RNA sequencing-based exploration of the effects of blue laser irradiation on mRNAs involved in functional metabolites of D. officinales
Source: PeerJ. 2022 Jan 4;10:e12684. doi: 10.7717/peerj.12684 (PMC8740519; doi:10.7717/peerj.12684)
Supplement: Supplemental Information 1 [file peerj-10-12684-s001.zip › Supplemental information/Table S3.docx]

**Table S3** The related genes of *D. officinale* under different light treatments

| New genes | Functional annotation gene | Differentially expressed genes |
| --- | --- | --- |
| 3735 | 2888 | 2888 |
