# Supplementary material for: RNA sequencing-based exploration of the effects of blue laser irradiation on mRNAs involved in functional metabolites of D. officinales
Source: PeerJ. 2022 Jan 4;10:e12684. doi: 10.7717/peerj.12684 (PMC8740519; doi:10.7717/peerj.12684)
Supplement: Supplemental Information 1 [file peerj-10-12684-s001.zip › Supplemental information/Table S4.pdf]

Table S4 The FPKM value of differentially expressed genes of *D. officinale* under different light treatments

Gene ID	White1	White2	White3	Blue1	Blue2	Blue3	Laser1	Laser2	Laser3
gene-MA16_Dca009641	1.658292	2.566069	2.702823	1.752238	1.445384	1.226888	5.332218	5.220523	5.85391
gene-MA16_Dca009643	6.366107	6.095078	4.896574	2.808374	2.070931	2.876619	6.632579	5.659909	6.734943
gene-MA16_Dca009646	1192.67896	1175.1842	1197.53308	41.888077	37.71384	35.746586	44.681957	37.02438	41.693089
gene-MA16_Dca009647	4.963569	4.905734	4.941891	2.667691	2.560257	2.861358	2.549768	1.788654	1.744635
gene-MA16_Dca026251	22.256199	18.588081	19.173079	61.30666	62.86042	57.360909	55.932018	55.511448	58.295048
gene-MA16_Dca008992	60.222038	61.691887	59.904976	12.622072	12.688202	13.339287	12.226173	9.616714	10.482379
gene-MA16_Dca008991	497.713562	528.463196	493.852386	108.213448	97.901497	103.495918	23.25696	22.384073	23.784172
gene-MA16_Dca008990	37.52261	38.171249	34.762077	2.752981	2.408365	2.360204	0.382433	0.554732	0.313289
gene-MA16_Dca012410	15.864329	14.827536	14.372485	2.999695	4.33265	2.687616	4.936836	3.939702	4.140668
gene-MA16_Dca012413	9.209277	8.855454	6.573822	10.17786	8.111316	8.717493	18.704493	17.593657	19.694538
gene-MA16_Dca020896	8.640729	7.50895	6.145148	26.787239	27.186148	19.278399	16.938633	20.633738	23.764071
gene-MA16_Dca025251	4.549541	3.28391	4.161408	0.51892	1.320536	1.31812	0.881471	0.775492	0.625336
gene-MA16_Dca025250	1.902134	1.629873	1.852466	3.174389	3.432197	3.12281	4.739033	4.256949	3.978157
gene-MA16_Dca020892	63.914749	57.092831	58.646255	34.235695	42.085255	38.192474	21.81605	26.079573	19.23315
gene-MA16_Dca021574	9.473094	8.91333	7.831327	8.58313	7.174891	8.828023	19.620003	20.934626	19.859753
gene-MA16_Dca015280	12.365815	10.728758	10.784815	0.020459	0.159006	0.021498	0.037865	0	0
gene-MA16_Dca019509	8.21371	7.930823	11.75119	1.549938	1.966963	1.309485	3.773072	0.982754	1.766729
gene-MA16_Dca010107	76.833946	78.072601	70.493317	51.521133	56.835587	55.342686	198.595856	201.059967	201.005051
gene-MA16_Dca010106	11.75796	8.923689	11.102122	3.941591	4.810604	4.301401	5.846513	4.929545	4.975471
gene-MA16_Dca010103	0.72742	0.722737	0.491137	0.904319	0.695959	0.640905	1.35637	1.546221	1.59735
gene-MA16_Dca010108	8.846334	7.391052	9.133506	18.335281	16.177519	16.566782	25.595552	22.004299	24.317308
gene-MA16_Dca022945	4.811902	6.345496	5.406061	6.299818	8.558266	7.164092	12.244042	12.578744	11.32727
gene-MA16_Dca017346	4.203266	2.892491	3.447994	6.717684	6.717107	8.717289	8.353167	10.062441	6.024189
gene-MA16_Dca005958	7.855642	7.431098	7.542043	1.441869	2.007339	2.25775	2.385247	1.881701	1.892134
Dendrobium_catenatum_newGene_1799	0.212817	0.229681	0.330536	0.936623	1.232625	1.128895	1.308633	1.235515	1.694221
gene-MA16_Dca007625	17.981915	17.946711	19.006697	4.636596	4.910979	5.079114	8.460003	8.58659	10.492419

gene-MA16_Dca009844	1.116616	1.115207	1.404874	4.630752	2.682794	3.599787	4.647179	3.479199	3.874746
gene-MA16_Dca009843	47.924683	44.290237	44.416336	22.372267	21.321177	20.639605	25.349398	24.447081	20.333166
gene-MA16_Dca001668	2.296948	1.75806	1.551104	3.601727	3.45463	3.919559	3.931347	4.774144	3.415979
gene-MA16_Dca005174	0.503559	0.419495	0.397798	0.674927	0.400579	0.445956	0.874814	1.172837	1.01381
gene-MA16_Dca000045	0.472249	0.281202	0	3.034295	3.693813	2.368793	1.572414	1.755504	1.456589
Dendrobium_catenatum_newGene_10915	14.6744	14.279026	15.05829	7.848177	8.312548	6.875352	6.649791	7.941234	8.150553
gene-MA16_Dca008711	23.482878	26.876564	23.389757	7.079996	7.363502	7.11382	5.548484	5.265635	6.37227
gene-MA16_Dca008712	29.902515	32.998302	28.185596	8.421226	4.465575	8.141365	7.87335	7.817937	6.635999
gene-MA16_Dca012279	7.196107	6.787878	7.701901	1.483114	1.162957	1.300712	0.452353	0.501694	0.616508
gene-MA16_Dca010699	10.099895	6.770992	9.380078	4.52771	5.199044	3.353396	6.165405	4.02638	4.535913
gene-MA16_Dca010690	5.388248	6.87534	5.865035	0.362105	0.813982	0.38628	0.306169	0.521354	0.194422
Dendrobium_catenatum_newGene_12798	3.33668	4.272061	4.331232	1.789617	1.674436	1.0416	0.876567	1.261503	1.342436
gene-MA16_Dca016694	18.58098	19.816908	17.806908	2.682855	2.416314	2.022469	2.033325	1.335331	0.575994
gene-MA16_Dca016691	0.90077	1.190654	0.909006	1.610578	2.909891	2.682132	1.346735	1.409899	2.074418
gene-MA16_Dca016698	20.098072	21.858664	20.235874	15.145338	12.755418	15.572883	8.127331	9.51425	8.473893
gene-MA16_Dca011568	2.356342	2.265993	2.46006	3.984618	3.014128	3.592095	5.886805	5.829269	6.401222
gene-MA16_Dca020931	35.188362	34.902096	35.067371	18.71072	15.984541	16.06753	15.149943	13.547636	14.912179
gene-MA16_Dca011563	11.841834	9.563985	14.348316	3.165042	4.879581	4.779254	11.525544	7.807665	6.508638
gene-MA16_Dca003538	13.255772	11.380824	12.43918	9.17534	5.787411	10.120182	3.398877	1.845998	5.539255
gene-MA16_Dca013586	23.505934	20.531414	21.595472	11.367783	14.619647	11.747747	9.47603	10.056181	11.250118
gene-MA16_Dca013582	24.174173	26.176321	27.551937	12.37529	12.67522	12.75561	19.876463	16.870197	18.165159
gene-MA16_Dca013637	13.551759	12.829635	13.043636	2.877093	2.824287	2.470899	1.331708	2.073071	2.114176
gene-MA16_Dca013631	4.275321	4.451621	4.715714	2.597911	3.162522	3.332059	2.58091	1.755337	1.588008
gene-MA16_Dca016036	116.443375	113.545486	115.074028	34.741646	31.06077	32.9715	31.181175	30.503811	28.532726
gene-MA16_Dca002370	26.38818	26.378073	29.822147	10.005492	10.54143	9.422817	16.72361	16.768669	18.459084
gene-MA16_Dca023560	2018.01563	2027.90967	2116.92358	842.958252	887.531677	874.049683	729.001404	736.898987	726.691895
gene-MA16_Dca023565	15.65853	17.65633	16.563852	30.172413	25.804211	26.19216	45.041401	47.453953	42.711617
Dendrobium_catenatum_newGene_2328	2.758285	2.06148	1.899723	2.194286	3.979694	3.017301	16.250065	16.562233	12.55974

gene-MA16_Dca015672	19.257647	20.941694	20.246506	1.785787	1.848893	2.408086	3.800844	3.301211	3.090129
gene-MA16_Dca015673	12.750539	11.365664	12.395491	5.598394	5.474911	4.891869	7.761062	5.009017	6.36345
Dendrobium_catenatum_newGene_663	0.229132	0.23881	0.190183	0.591212	0.421338	0.932317	0.409485	0.535065	0.591715
gene-MA16_Dca015090	5.192533	5.051192	5.321474	2.257453	2.386121	2.198416	1.961554	2.459822	1.620336
gene-MA16_Dca015091	42.45253	45.749458	42.05687	10.488151	10.064028	12.418077	22.978983	22.525957	23.365225
gene-MA16_Dca006117	146.874664	137.037811	154.073227	304.821472	307.663177	310.133667	537.113525	485.152039	479.382599
gene-MA16_Dca006653	13.947125	14.417376	13.117318	14.033447	17.682348	18.916916	9.112082	6.861753	6.433334
gene-MA16_Dca001462	0.30534	0.323203	0.319962	0.451463	0.326843	0.547465	1.007641	0.849136	0.864755
gene-MA16_Dca002171	0.728026	0.949857	1.187714	0.271781	0.257977	0.271468	0.581886	0.382459	0.302673
gene-MA16_Dca012847	10.677165	11.906968	11.846411	17.501682	16.930986	16.044388	73.504875	81.983231	79.531662
gene-MA16_Dca002826	2.240414	2.586104	4.050134	7.091232	8.724222	5.895685	1.695149	4.088308	2.280719
gene-MA16_Dca002823	8.5839	7.949702	7.413487	3.605383	3.503112	1.373568	2.129306	1.171219	1.746047
gene-MA16_Dca002828	27.863688	25.450317	28.758072	9.693532	9.860595	11.713637	9.926217	12.578808	10.787671
gene-MA16_Dca012607	2.769793	3.189788	2.189731	2.861794	3.137383	2.379113	0.394954	0.694716	0.692885
Dendrobium_catenatum_newGene_14515	3.129221	4.463377	3.011333	1.033371	1.352805	1.034748	0.613712	0.91065	0.276247
gene-MA16_Dca003709	110.204895	109.904518	106.426895	56.635174	61.63792	51.97797	36.551422	38.490597	34.341663
gene-MA16_Dca027052	226.772934	252.044495	242.417023	70.919479	73.639313	75.328148	65.489738	55.042225	57.353199
gene-MA16_Dca003703	4.402529	4.254477	4.212016	2.015804	1.137972	1.815851	3.924277	1.056631	0.834749
gene-MA16_Dca014460	0.345566	0.052117	0.194225	0.217573	0.827404	0.488023	2.476834	2.172096	1.99018
gene-MA16_Dca014467	0.772448	0.790118	0.497206	1.642915	1.721684	1.524964	3.671255	2.835669	3.737098
gene-MA16_Dca014464	3.818422	3.150934	3.577317	0.413095	0.045521	0.247503	2.230505	1.374722	1.642387
gene-MA16_Dca026096	1.915039	1.682746	2.081591	1.656702	1.579142	1.317073	0.202032	0.795559	0.453164
gene-MA16_Dca027942	17.260365	18.524574	18.785671	7.35706	5.958388	7.157195	6.142989	5.249653	5.313179
gene-MA16_Dca025545	2387.65796	2431.0647	2388.3877	221.677521	217.91098	217.671677	130.556564	122.724495	120.06498
gene-MA16_Dca019218	49.720673	48.687817	48.359608	18.028357	15.880489	17.945124	16.012732	14.884919	15.657144
gene-MA16_Dca024635	38.33532	38.808247	30.83429	5.466505	3.234308	4.199501	0.479427	0.66818	1.315879
Dendrobium_catenatum_newGene_481	3.021147	4.648329	3.131685	0.636001	0.403221	0.500174	0.760081	0.810815	0.595268
Dendrobium_catenatum_newGene_8579	2.053676	3.868573	2.651651	12.877064	11.427227	10.968602	5.630397	4.21304	3.874892

gene-MA16_Dca021044	12.363139	11.226523	11.515233	1.316637	1.270529	1.171375	2.670722	1.942601	2.703953
gene-MA16_Dca020021	72.257988	76.360825	74.324654	36.380501	36.59206	36.093815	28.808344	26.905968	27.841579
gene-MA16_Dca020020	6.080023	6.979284	7.917366	2.914343	2.572452	3.866099	2.460761	2.206023	1.661805
gene-MA16_Dca007470	4.238676	4.966752	3.183223	7.813809	7.432941	8.568675	24.649862	20.951815	22.606653
gene-MA16_Dca007475	0.616389	0.738758	0.819514	1.15546	1.742121	1.806801	3.461602	3.998134	4.762926
gene-MA16_Dca006705	87.146843	69.508415	79.094215	27.11031	54.080063	19.060892	20.171965	34.395348	35.323418
gene-MA16_Dca004412	1.883419	1.585358	2.117036	0.87776	1.152509	0.966522	0.221143	0.74139	0.320385
gene-MA16_Dca008560	16.378105	17.570236	16.627827	19.576805	20.484348	21.190123	42.376381	41.043705	43.570705
gene-MA16_Dca003142	1.150718	0.529362	0.833833	1.907776	1.701106	1.65668	3.458775	3.849427	2.667902
gene-MA16_Dca010955	28.901834	27.54076	29.119688	17.843035	17.799997	11.877248	12.943532	10.079637	12.523608
gene-MA16_Dca008919	5.047843	4.468331	4.789759	8.295363	7.456138	9.128947	10.658237	12.404647	11.005762
gene-MA16_Dca013981	24.574278	22.676517	22.872581	57.087589	55.462582	56.988861	54.558327	54.650627	54.943886
gene-MA16_Dca013984	4.238015	5.023045	5.37918	1.46168	3.601055	0.912546	3.157988	4.668092	4.083761
gene-MA16_Dca020627	5.736387	5.310966	6.657529	0.329396	0.037074	0.392512	0.058978	0.031412	0.109499
gene-MA16_Dca020624	0.706504	0.961644	0.794811	1.430405	0.98539	0.21489	0.250836	0.400771	0.268117
gene-MA16_Dca011271	23.765953	25.341496	25.325998	13.560681	10.371207	12.355282	10.047375	10.965225	9.965454
gene-MA16_Dca016729	24.003485	18.406065	22.286373	4.61261	5.035272	5.965602	2.044032	1.77122	2.496094
gene-MA16_Dca012614	35.509739	37.110489	33.889282	4.104381	4.27173	5.000117	0.857959	1.39588	1.233901
Dendrobium_catenatum_newGene_11547	0.759484	0.791741	0.876463	0.340699	0.3109	0.431876	0.541573	0.575472	0.59297
gene-MA16_Dca014617	2.587677	2.19898	2.994057	2.002421	1.609629	1.0506	0.991143	0.891012	0.915108
gene-MA16_Dca014616	7.171559	6.462214	7.562139	11.093671	6.654712	6.911003	21.465826	21.1222	17.29356
gene-MA16_Dca014618	32.851116	29.713919	27.881804	9.811207	11.469394	10.428107	20.440294	19.218363	17.335413
Dendrobium_catenatum_newGene_178	0.363339	0.808848	0.847871	1.261161	0.842504	0.700758	3.023908	3.070716	2.306357
Dendrobium_catenatum_newGene_1719	11.700722	13.159568	16.970078	16.061218	15.159207	13.255983	35.345932	33.994884	31.925236
Dendrobium_catenatum_newGene_9528	8.848422	10.29142	11.691975	1.072114	2.678124	0.547772	1.494981	5.192012	3.626515
gene-MA16_Dca019022	0.342072	0.352483	0.175524	0.274268	0.340041	0.45495	1.049972	0.974205	0.776951
Dendrobium_catenatum_newGene_8438	0.732814	0.364827	1.041679	0.578561	1.693355	1.014596	1.881335	2.665396	1.442078
Dendrobium_catenatum_newGene_8434	4.93785	4.858305	3.642577	7.250889	6.752233	7.833336	10.858386	12.651432	9.090528

Dendrobium_catenatum_newGene_8977	1.689919	2.043409	2.418211	0.499538	0.641973	0.736345	0.437281	0.61909	0.797794
gene-MA16_Dca013825	19.542542	16.38175	15.66994	7.386106	9.062351	9.445529	9.452417	8.504608	8.020698
gene-MA16_Dca013820	22.826519	23.869162	23.978956	31.879038	30.498665	30.445707	117.103165	117.868805	103.645088
gene-MA16_Dca005245	9.002891	7.76614	7.133031	9.643361	8.518081	11.580675	15.51303	17.765863	19.34705
gene-MA16_Dca008245	21.730453	20.942291	20.967651	8.378895	8.534606	10.887348	39.591808	34.963795	39.301651
gene-MA16_Dca002661	1.467618	2.817765	2.234192	4.845007	3.664736	3.656358	4.903422	6.608046	6.940672
gene-MA16_Dca015741	7.113328	6.992909	6.737965	0.863933	0.611819	0.606503	1.205759	1.057763	1.437422
gene-MA16_Dca010850	2.614703	4.127182	2.826582	1.682801	2.081191	1.615915	4.61981	4.607056	6.156195
Dendrobium_catenatum_newGene_16659	32.60548	32.056656	30.819283	45.458096	43.605286	44.369789	72.844742	78.011597	80.177628
gene-MA16_Dca012753	13.731807	13.692368	12.268743	7.004754	5.671819	7.504566	12.124527	9.870992	9.417773
gene-MA16_Dca026354	13.326223	16.013737	10.956358	50.441807	43.914192	47.28397	47.900238	47.72015	48.820759
gene-MA16_Dca023653	22.223053	24.140352	23.059937	14.282666	13.488003	15.11871	11.104266	8.733317	8.884228
gene-MA16_Dca023644	16.764593	15.605596	15.797394	37.82045	43.64827	38.856693	61.21505	45.500717	53.670139
gene-MA16_Dca022290	146.367355	147.876266	145.8423	82.389359	90.700188	89.994865	43.945774	34.928314	45.113724
gene-MA16_Dca022293	221.607513	228.916382	225.284546	111.171593	111.779198	111.754044	99.139252	95.882263	94.356209
gene-MA16_Dca022295	1206.55237	1175.61719	1199.77844	322.226562	334.65033	324.805725	246.411835	229.565186	226.095261
gene-MA16_Dca024868	8.730331	6.81085	7.149791	3.542828	3.719135	4.498582	5.535723	5.324213	6.294759
Dendrobium_catenatum_newGene_2902	7.429216	8.899996	8.289098	3.117679	2.782185	2.195321	2.591785	2.785398	3.45918
gene-MA16_Dca014361	7.479127	10.410995	7.865105	12.073659	10.608933	12.358958	21.284945	19.377468	18.409397
gene-MA16_Dca019625	256.782227	256.195618	268.428619	1.907475	2.534703	2.691611	11.389315	7.038892	8.298379
Dendrobium_catenatum_newGene_11041	8.779991	9.044375	8.372605	6.556789	6.860123	6.66619	2.710454	1.850482	2.492778
gene-MA16_Dca024241	37.123688	39.760685	39.554115	18.329325	19.128918	18.031672	18.409786	17.027588	17.977123
gene-MA16_Dca027741	0.811224	0.661792	0.686084	0.777641	1.273482	1.202694	1.595664	1.560405	1.67393
gene-MA16_Dca000214	4.560983	6.004847	6.479138	35.448017	32.328579	34.153702	57.971355	54.697426	59.296318
gene-MA16_Dca000211	3.000499	1.944998	4.20592	10.939588	6.810061	9.343085	15.468883	15.90309	20.087645
gene-MA16_Dca000213	447.439331	463.30426	450.635223	159.382812	157.839325	152.899994	163.674194	165.082413	162.141251
gene-MA16_Dca018236	7.495198	7.490864	6.777834	8.809406	8.629836	9.900806	16.848145	14.235588	14.913707
gene-MA16_Dca018232	3542.8689	3605.55566	3406.12427	1774.57776	1713.88403	1714.0647	1390.90784	1409.23267	1404.93543

gene-MA16_Dca025873	0.77387	0.407594	0.67813	1.249723	1.302702	1.935862	1.076051	1.129309	1.206677
gene-MA16_Dca005858	125.632187	123.525253	121.227722	26.417273	24.387667	27.529501	21.937557	21.587961	18.387304
gene-MA16_Dca006866	2.444879	2.522476	2.232006	0.244893	0	0.34242	0	0.126135	0
gene-MA16_Dca012064	10.396387	11.850069	10.263147	5.784807	5.427629	4.613011	5.582949	5.500383	4.935394
Dendrobium_catenatum_newGene_6737	3.014536	2.764257	2.23494	1.208747	1.611061	1.738263	1.076673	1.248059	1.625067
gene-MA16_Dca003841	6.862239	5.503376	3.361273	1.309555	0.331048	0.646486	0.227678	0.493474	1.088048
gene-MA16_Dca003000	32.252464	28.10441	31.785576	6.327178	10.884755	8.375478	22.710356	12.950989	17.434231
gene-MA16_Dca007544	6.318885	6.0107	7.837581	13.218139	14.422477	12.546339	14.770327	22.911169	14.002497
gene-MA16_Dca007549	1.955548	1.578927	1.289571	3.004873	3.504802	2.562178	6.574948	3.895455	4.376661
gene-MA16_Dca003002	2.268007	2.191898	1.887249	0.11054	0.063884	0.102518	0.234293	0.354741	0
Dendrobium_catenatum_newGene_3617	8.646739	7.766114	8.013858	2.417367	1.845542	1.775366	0.809913	0.873553	0.781013
gene-MA16_Dca005036	107.35215	96.015953	102.886116	90.790482	90.886482	83.028305	36.717152	34.709213	27.920561
gene-MA16_Dca005033	23.7894	25.247986	23.939861	6.216903	6.118084	8.901501	10.712378	12.137855	12.282277
gene-MA16_Dca005030	107.772629	104.835686	114.263725	166.822662	167.356323	165.798752	248.163818	242.910278	228.188354
gene-MA16_Dca018655	6.681031	7.604597	7.038932	4.229957	3.072587	3.560448	4.283439	4.152862	3.818067
gene-MA16_Dca016957	11.029781	12.940441	9.083063	5.379364	5.431588	6.82313	3.511517	4.865557	3.964216
gene-MA16_Dca016950	94.205345	77.880768	92.266724	18.953205	11.100581	19.99865	18.671	15.836651	20.100853
gene-MA16_Dca003471	0.660258	0.313421	0.868782	1.289919	0.733335	1.613842	4.778308	3.10504	3.956651
gene-MA16_Dca003472	119.164993	124.822136	119.488625	145.213776	137.277634	134.568115	63.25317	58.085396	63.392235
gene-MA16_Dca026649	10.062496	10.555089	12.202906	4.184744	3.762376	2.367464	4.05512	6.557108	5.22093
gene-MA16_Dca022822	2.289684	2.275541	2.071321	0	0	0.020238	0.05423	0	0
gene-MA16_Dca022821	52.861141	53.577187	52.261574	1.450215	2.469512	1.373904	3.078564	1.420292	2.445362
gene-MA16_Dca023448	64.144676	63.466618	65.774727	17.250164	16.290842	17.241837	30.112104	33.371307	31.639521
Dendrobium_catenatum_newGene_10821	6.662258	6.348441	7.580664	27.433475	28.012413	31.382768	70.469737	61.960927	62.077546
gene-MA16_Dca017779	17.228264	18.797699	17.861706	9.668708	12.396991	10.977446	8.54019	7.287602	7.805451
gene-MA16_Dca026050	520.448486	520.62085	518.74231	226.131119	210.271393	227.965469	390.031097	361.332428	379.203461
gene-MA16_Dca013291	2.689059	2.739746	3.03121	1.929736	1.185942	1.931035	1.527482	1.57162	1.091305
gene-MA16_Dca013294	2.518695	2.715612	3.08262	0.296512	0.523823	0.256281	0.270076	0.333983	0.359604

gene-MA16_Dca013296	17.229006	15.858603	15.292431	3.391899	4.114486	3.395364	2.980499	2.934363	2.832683
gene-MA16_Dca018749	63.122478	60.592896	62.975906	67.932541	62.43182	65.776733	24.278498	21.908884	22.729254
gene-MA16_Dca018748	1.339821	0.837953	0.904582	2.269755	1.299125	1.962605	3.213668	2.628397	1.932967
gene-MA16_Dca025096	2.459718	2.027198	2.678389	3.949678	3.357455	4.145884	7.781981	6.799569	5.99374
gene-MA16_Dca015198	4.67144	4.965937	4.672152	9.114257	9.47165	9.514088	14.665153	15.539238	14.152398
gene-MA16_Dca025326	87.018547	90.162346	90.236351	101.683861	121.705589	95.41996	191.669434	190.215302	180.388245
gene-MA16_Dca020314	46.40789	47.892635	46.123772	84.152687	77.554413	80.21064	25.556601	23.078489	22.164566
gene-MA16_Dca007941	15.784923	15.569082	14.40264	7.837721	9.072017	7.704155	6.577209	4.816784	7.277586
gene-MA16_Dca004376	3.578394	3.476745	4.160467	5.009146	6.931222	5.549756	8.770145	8.5919	9.070062
gene-MA16_Dca002239	1910.03259	1940.08386	1959.69666	1135.19043	1146.15332	1127.97888	997.542603	955.096802	980.674011
gene-MA16_Dca009065	181.860748	176.140503	169.562042	37.026695	39.429256	40.153328	73.727768	67.820824	71.077927
gene-MA16_Dca006261	59.825027	63.6968	65.800858	30.306126	34.692009	34.634903	29.582573	30.115253	32.508625
Dendrobium_catenatum_newGene_3423	0.704201	0.962204	0.947303	2.162371	2.052449	2.085981	1.508184	1.272278	1.119129
Dendrobium_catenatum_newGene_5869	0.942513	0.937604	0.767003	1.493497	1.663312	1.829485	2.660357	2.792852	2.866224
gene-MA16_Dca010766	19.06148	20.816034	18.65963	33.514557	33.438705	32.794674	44.211727	43.238064	43.546398
gene-MA16_Dca023768	19.572203	16.498741	17.084135	25.576815	34.25388	23.878635	55.340866	57.702446	58.85643
gene-MA16_Dca026195	0	0.080965	0.258407	0.169742	0.805613	0.544571	1.967962	1.719849	1.2279
gene-MA16_Dca026198	124.467888	126.471603	123.164108	30.493629	28.785198	24.926641	32.397064	30.002583	30.127775
gene-MA16_Dca027862	12.238776	12.035643	11.438347	5.049982	5.459836	5.389506	5.743677	5.479286	4.950338
gene-MA16_Dca015404	11.363853	12.809128	12.672411	18.022257	17.178896	17.176174	27.817875	28.959772	28.584389
gene-MA16_Dca025427	1.091564	1.492409	0.517873	2.166934	2.250855	2.132242	17.599218	18.381987	19.110945
gene-MA16_Dca025424	1.753779	2.693769	2.285885	4.431488	4.833064	4.330845	5.607817	4.61374	5.61764
gene-MA16_Dca017920	4.693549	4.39219	4.087259	7.808829	8.179956	6.45933	13.50716	13.114467	15.831872
gene-MA16_Dca017921	7.830634	7.610195	7.296832	10.066652	11.950611	12.107107	24.803402	26.185631	25.348551
gene-MA16_Dca026733	28.286686	22.534367	22.877234	1.024782	1.114003	0.966574	1.06719	1.032243	1.313436
gene-MA16_Dca024777	6.003364	4.398547	4.764089	2.34381	0.774823	2.030174	2.242899	2.751791	1.866376
gene-MA16_Dca023861	1.82575	2.1388	2.332726	0.411637	0.973656	1.035301	0.826388	0.93409	0.971304
gene-MA16_Dca012184	3.420974	3.268788	3.879191	0	0	0	0	0	0

Dendrobium_catenatum_newGene_710	7.813807	6.549833	6.761043	3.776905	4.35705	3.546984	3.331672	3.045914	3.11692
gene-MA16_Dca020107	76.915894	70.869568	71.72554	129.51593	120.652367	124.601387	254.042267	238.123077	241.160614
gene-MA16_Dca020104	3.493709	4.968814	2.836146	5.213854	9.310731	10.031557	9.275866	12.548754	11.913565
gene-MA16_Dca027453	10.808982	10.677159	10.304626	4.520122	6.614183	4.380721	4.839518	3.644601	3.884406
gene-MA16_Dca027452	1661.33289	1504.07666	2273.80664	590.825012	1073.30286	628.265442	1231.48096	992.574646	1026.76135
Dendrobium_catenatum_newGene_8958	1.928315	1.130446	1.321844	4.660218	3.567276	2.862543	5.933751	6.109972	5.100896
Dendrobium_catenatum_newGene_8956	4.623426	4.285929	3.991467	0.341797	0.12456	0.174875	0.204225	0.074545	0.166969
gene-MA16_Dca006065	3.409974	3.907326	3.6301	7.748889	7.4024	7.509891	10.204731	11.80906	10.796724
gene-MA16_Dca006063	8.481469	7.161865	6.977128	10.764285	11.007068	10.836043	22.354589	20.152365	17.186569
gene-MA16_Dca006062	86.012161	92.873032	91.70314	43.127266	45.282543	48.814842	36.199444	35.663273	38.9039
gene-MA16_Dca004511	97.83709	90.334267	95.042343	125.385262	131.38736	137.766602	206.519714	200.223297	223.760864
gene-MA16_Dca001590	52.87883	49.790417	48.100636	80.511696	78.267303	79.335556	118.59893	116.955627	117.015808
gene-MA16_Dca001591	1.916126	2.107506	2.136103	3.364202	3.152975	3.24485	5.154783	5.574709	4.251137
gene-MA16_Dca001046	114.206688	119.99633	112.981537	45.048969	57.696667	45.065948	36.31852	33.168549	28.072006
gene-MA16_Dca001048	146.618607	154.962341	139.522476	77.938362	74.591217	75.612373	102.053711	102.957306	97.050499
gene-MA16_Dca005740	0.31094	0.134806	0.31328	0.270183	0.515296	0.417271	1.444969	1.345396	0.935798
gene-MA16_Dca016529	25.275816	27.249311	24.49707	11.883848	8.031821	9.135067	1.756264	2.590823	1.644661
gene-MA16_Dca009380	1.52354	1.021506	2.796734	4.117688	3.384307	2.811982	4.321981	4.951344	5.610135
gene-MA16_Dca009384	0.308374	0.320866	0.449372	0.815503	0.611357	0.810977	1.048897	1.262155	1.091474
gene-MA16_Dca007073	77.313622	77.469528	70.859581	34.687851	31.464457	30.318048	37.570595	38.167099	32.761124
Dendrobium_catenatum_newGene_6350	1.632972	1.057534	1.089851	0.857533	0.875966	0.652039	0.169681	0.367383	1.138154
gene-MA16_Dca013009	11.72116	11.30366	10.968534	7.303444	8.411302	7.920752	5.317327	5.919452	5.607511
gene-MA16_Dca013008	169.61731	148.132156	162.885071	167.357712	177.895447	148.344254	393.056061	377.310883	376.513336
Dendrobium_catenatum_newGene_2635	7.446256	6.800407	7.22574	1.818428	1.639419	1.488659	2.636628	1.842415	2.080906
Dendrobium_catenatum_newGene_4677	2.942849	4.174388	4.53357	1.319999	3.375437	1.097406	1.081136	1.043313	0.558012
gene-MA16_Dca000547	6.703961	8.00497	7.112604	13.103999	13.501674	11.659259	16.252195	15.449968	16.278076
gene-MA16_Dca000541	15.129352	13.579083	15.382708	20.777861	18.563089	28.409908	40.845875	51.001202	54.108959
gene-MA16_Dca027123	0.407416	0.477583	0.284528	2.492834	2.224434	2.322815	1.534906	0.661331	1.133225

gene-MA16_Dca012154	0.41498	0.469669	0.741807	1.562399	1.392289	0.635942	1.219653	1.88308	1.340336
gene-MA16_Dca023354	1.670913	1.923087	1.698568	4.71528	3.752436	3.764784	3.803962	2.59629	2.428141
gene-MA16_Dca022407	2.646155	2.960675	3.071916	5.840163	5.220431	5.718315	20.432537	15.278643	14.468274
gene-MA16_Dca022406	14.239972	13.673553	13.88761	4.648391	4.741949	6.239476	18.154694	16.546169	16.543728
gene-MA16_Dca017689	3.306089	2.947726	3.235325	8.338638	7.910751	8.180756	6.098807	6.847921	6.300244
gene-MA16_Dca014082	114.413925	119.853111	117.221802	6.231662	6.457825	7.104795	4.025798	5.256289	4.354627
gene-MA16_Dca018850	2.659101	2.294297	3.344862	1.100032	1.195583	0.832997	1.765008	1.564861	1.494903
gene-MA16_Dca015685	7.288418	8.762541	6.897025	4.16611	3.620675	3.115715	7.198681	6.819463	8.076762
Dendrobium_catenatum_newGene_8772	4.234008	3.169416	3.119578	5.895502	5.740876	6.956009	8.724307	9.670753	7.638117
gene-MA16_Dca004897	4.406164	4.161916	3.420366	5.119334	4.440021	4.929059	18.576004	15.401467	14.330288
gene-MA16_Dca004893	8.54538	12.305061	7.353747	1.221621	1.804851	2.293654	1.229053	0.95882	0.766349
gene-MA16_Dca004890	63.196629	64.751671	62.695198	44.881947	43.21373	44.843094	34.072308	32.14082	32.314491
gene-MA16_Dca020734	0.543969	0.119052	1.204739	3.507329	2.243914	3.443012	2.195024	2.706674	3.11534
gene-MA16_Dca001292	1.800651	1.570033	2.135369	3.253557	3.469544	4.897256	6.163455	5.591477	5.590405
gene-MA16_Dca007271	7.434366	9.003416	8.513214	6.059476	5.83328	6.385664	4.071405	2.872201	3.967427
gene-MA16_Dca007277	98.54673	92.41481	101.865456	18.657505	18.553885	18.07082	18.163414	15.352233	14.548734
gene-MA16_Dca007274	1.441621	1.762321	1.279078	0.93054	0.427963	0.4508	1.075797	1.218566	0.80484
gene-MA16_Dca015027	21.364756	20.7356	13.719336	19.205076	14.568161	17.544271	9.51307	8.865909	8.262714
gene-MA16_Dca008360	2.657696	1.80094	2.420908	0.712229	0.230972	0.446861	0.151837	0.314964	0.107
gene-MA16_Dca003037	7.78509	5.453685	7.671699	0.792578	0	1.050403	3.761733	1.43941	0.989735
gene-MA16_Dca001361	32.471436	32.321648	29.572701	2.133877	1.950404	2.045045	1.402208	0.708046	2.015737
gene-MA16_Dca003030	86.746193	88.993752	91.313957	71.779175	68.511719	66.599144	43.838463	48.019997	45.364319
Dendrobium_catenatum_newGene_3878	6.682134	6.645726	7.461062	13.450514	14.942072	13.165996	16.251399	14.735229	15.282365
gene-MA16_Dca002706	10.346064	10.589922	9.908044	5.097908	5.509735	5.07971	8.402285	8.676934	8.457743
gene-MA16_Dca002707	1.910859	1.656813	1.804559	0.102799	0.358398	0.137989	0.165628	0.246381	0.27683
gene-MA16_Dca002708	3.208488	2.402995	3.178525	5.26006	4.837774	4.954382	6.686518	8.837219	8.856949
gene-MA16_Dca003110	1.275876	1.536465	0.977979	0.813539	0.917797	0.651799	0.317949	0.504134	0.834763
Dendrobium_catenatum_newGene_1464	85.765961	80.129692	87.391762	42.763138	42.057934	41.616631	41.55991	35.83152	44.029274

Dendrobium_catenatum_newGene_1461	0.98605	0.756592	0.760497	1.724015	1.959051	1.846154	1.231301	1.055996	1.103395
gene-MA16_Dca004285	176.271286	170.101715	166.609024	48.742554	52.172909	57.114407	20.333876	17.319166	10.836575
Dendrobium_catenatum_newGene_16467	12.768607	12.800227	12.171169	3.53236	3.641475	4.328072	2.642265	2.607491	2.497951
Dendrobium_catenatum_newGene_12372	8.043965	9.507688	6.120464	23.637775	26.764748	22.717142	16.559252	18.723009	16.44779
gene-MA16_Dca011912	149.721786	152.683548	153.321472	261.130035	262.957458	253.558609	361.604156	355.659241	341.234711
gene-MA16_Dca011915	4.532511	4.567214	4.320053	6.484455	6.460792	6.813075	8.485785	8.571712	11.819341
gene-MA16_Dca022396	7.071723	4.714296	5.870857	0.081995	0	0.409011	0.439123	0.235415	0.273648
gene-MA16_Dca008821	2.322802	2.773945	3.421168	4.029626	4.233087	3.953648	6.148891	6.14921	6.240673
Dendrobium_catenatum_newGene_11111	3.610821	3.308976	3.576839	4.146888	4.395349	4.830475	8.938966	8.132433	7.006208
gene-MA16_Dca025017	1.027098	0.497382	0.420539	1.809148	2.128715	1.949613	0.814063	1.616293	1.168128
Dendrobium_catenatum_newGene_230	1.343638	1.278621	1.593014	0.149488	0.381526	0.116292	0.072963	0.130068	0.456728
gene-MA16_Dca013217	136.980835	129.195541	142.793533	53.388069	59.847702	55.888622	47.795662	47.583134	43.970383
gene-MA16_Dca015119	24.607077	29.464718	24.255701	7.803403	12.025157	9.669583	11.368976	5.456651	11.794483
gene-MA16_Dca019470	2.219819	2.261982	2.406603	1.036363	1.013085	0.577231	0.629547	0.630113	1.264649
gene-MA16_Dca020532	77.361237	83.35685	83.549011	24.344671	20.158585	24.939552	26.424391	27.485149	24.043465
gene-MA16_Dca027627	86.315277	95.133873	87.955162	129.421921	129.871399	122.990479	267.418335	257.556427	263.087677
gene-MA16_Dca015801	17.270201	17.525225	17.593023	4.948707	4.109403	5.126971	6.007466	6.243844	5.708197
gene-MA16_Dca015808	90.69873	87.760162	89.978188	28.645933	26.018818	27.170486	21.323284	21.766745	17.596394
gene-MA16_Dca022665	2.832663	3.472669	3.118331	8.643416	7.591738	10.123178	7.74286	7.498069	4.877554
Dendrobium_catenatum_newGene_16703	3.045824	3.606414	3.744803	2.546554	1.876248	2.267938	0.914842	1.523789	1.777164
gene-MA16_Dca026025	56.769325	55.438282	57.745525	30.277548	29.096914	27.980518	24.086281	29.54282	28.372629
gene-MA16_Dca014538	4.005242	4.511627	4.689315	5.904057	5.274292	5.201257	12.314268	11.298425	11.622485
gene-MA16_Dca014537	1.873595	1.047327	1.964401	3.875028	3.144202	2.731858	7.478746	5.188999	7.319672
gene-MA16_Dca019731	21.964045	20.897247	18.653975	12.347671	12.36586	14.302126	11.017974	8.756812	10.457029
gene-MA16_Dca021349	10.882627	8.957353	14.278018	1.253541	1.447535	0.812776	0	0.460486	0.200492
Dendrobium_catenatum_newGene_1664	8.925074	8.919895	11.150193	4.872208	3.151479	4.034495	4.004497	3.252717	3.860888
gene-MA16_Dca004914	55.835949	58.75576	58.447212	89.086243	86.577591	88.606071	130.339951	127.086334	130.33667
Dendrobium_catenatum_newGene_7541	1.484905	2.204804	1.836377	2.401323	2.831643	3.185046	4.595843	4.160806	3.48291

gene-MA16_Dca008782	2.796165	2.194438	2.406086	4.623737	4.048658	3.889519	6.864673	6.482496	5.782689
gene-MA16_Dca008783	8.720615	8.347756	7.37063	38.15086	35.583481	37.524086	48.551651	48.006336	50.293453
gene-MA16_Dca002989	1.473424	1.104352	1.47394	0.838709	0.433339	0.648985	1.070597	1.284816	0.732812
gene-MA16_Dca006408	96.901291	89.157532	90.87265	54.969166	53.493458	55.971554	44.921307	42.654114	45.556698
gene-MA16_Dca001750	204.598373	198.523697	193.958511	76.240883	77.223877	81.440094	47.724125	48.505913	52.913109
Dendrobium_catenatum_newGene_2743	508.854248	508.185181	570.432465	243.529034	250.639324	241.343211	218.665971	235.599167	243.055535
gene-MA16_Dca010457	77.091759	81.522568	78.402328	3.999347	4.16607	4.358573	5.430338	3.918069	3.091871
gene-MA16_Dca012349	307.660278	297.939148	298.841949	450.593445	438.458374	434.572998	673.414124	632.891724	650.762573
gene-MA16_Dca012343	37.557217	40.844311	50.753872	17.766821	16.646173	19.061094	23.003824	21.121185	19.836641
gene-MA16_Dca013438	89.122177	86.64772	88.24823	45.500488	47.249695	49.676693	43.782902	40.760601	37.366428
gene-MA16_Dca000936	1.012682	0.91452	0.740129	0.536153	0.813321	0.306206	5.436548	6.967587	5.674837
gene-MA16_Dca023091	43.017014	39.901051	39.9697	2.407141	1.586577	2.291636	1.478651	1.198334	2.086586
gene-MA16_Dca016149	0.865841	0.768711	0.618445	1.056879	0.60495	0.807716	2.210323	1.855045	1.661149
gene-MA16_Dca026666	142.576828	147.520309	144.386627	5.479687	6.605347	4.588611	8.029406	8.501561	7.656904
Dendrobium_catenatum_newGene_11093	0.661603	1.529108	1.028011	0.812269	0.615805	0.732117	0.085788	0.202024	0.349849
gene-MA16_Dca024403	47.223866	43.278793	45.080486	19.230038	17.178143	17.542439	16.325897	15.911658	14.635517
gene-MA16_Dca014162	21.335356	23.603275	23.090242	63.90218	59.840954	63.899849	77.40255	72.556267	73.194862
gene-MA16_Dca025200	8.073725	7.256897	10.003392	4.453538	4.228275	4.157125	5.062358	5.414163	6.701043
gene-MA16_Dca001510	4.893055	7.475761	6.999546	2.007175	2.403239	2.099967	2.757237	2.238218	2.397241
gene-MA16_Dca001511	3.851882	4.56981	4.602582	14.478857	13.648626	13.167067	17.746099	25.676208	22.560135
gene-MA16_Dca022935	10.59439	10.867541	7.762828	3.064256	3.350957	3.231119	2.441514	4.081083	4.060902
gene-MA16_Dca018340	5.089595	4.432902	3.770317	8.251331	7.014762	7.055127	9.888291	8.130802	10.555983
gene-MA16_Dca018344	0.856803	0.998957	1.531303	2.203566	2.631888	2.361392	5.270888	3.78842	4.279475
gene-MA16_Dca006886	3.56479	4.523133	3.925926	4.850202	5.410419	5.199476	17.514982	18.928617	15.559177
Dendrobium_catenatum_newGene_9590	37.724129	36.666126	35.725456	4.798561	6.997639	6.302513	2.711557	2.732475	3.631899
gene-MA16_Dca014034	5.527428	6.2225	5.647761	13.859383	12.225949	14.434786	19.817974	17.666439	19.80003
gene-MA16_Dca025745	10.234913	9.639657	8.78399	2.616171	2.127146	1.722164	3.006016	3.529877	2.188549
gene-MA16_Dca025744	15.920968	16.707439	19.092176	13.087358	11.597659	12.613782	31.503658	27.728231	25.756603

Dendrobium_catenatum_newGene_6024	4.882432	6.694026	5.441566	3.306891	1.883389	2.77333	2.322168	1.882281	1.679656
gene-MA16_Dca006308	7.754073	8.942397	8.050686	6.257235	4.875738	4.881703	4.122134	3.160455	2.752733
gene-MA16_Dca009872	14.349888	14.399882	12.320138	25.962917	24.935143	26.55686	30.932709	30.004986	31.592859
gene-MA16_Dca010397	28.358528	28.593313	27.889185	21.983921	20.480717	22.499537	14.105028	13.665264	13.977147
gene-MA16_Dca010393	5.388101	4.492794	3.781018	6.494824	6.353464	6.782239	8.381884	9.627333	12.080458
gene-MA16_Dca000010	4.587956	3.993433	4.677005	0.200981	0	0.701871	0.271789	0.450908	0.117865
gene-MA16_Dca000019	1.027167	0.842471	0.519171	0.925542	0.75904	0.965713	2.3647	2.809879	2.524572
Dendrobium_catenatum_newGene_5013	2.29782	2.284471	2.827226	1.292949	1.404335	1.224984	0.812781	1.07028	0.839837
Dendrobium_catenatum_newGene_5014	2.88852	2.033785	2.071954	1.283573	0.976806	1.46439	0.689694	0.58479	0.6087
gene-MA16_Dca009464	5.814496	5.40186	4.561016	11.518147	13.340556	17.104334	17.751581	17.496815	16.350054
gene-MA16_Dca009469	2.299642	1.979594	2.087144	1.732594	2.822944	1.568362	5.688942	5.099192	4.933382
gene-MA16_Dca002325	1.816163	1.889038	1.369925	0.131181	0.442032	0.937761	1.024543	0.842273	0.974625
gene-MA16_Dca018585	8.16694	7.971194	8.805899	1.122398	0.703559	0.57484	0.777311	1.170588	0.970462
Dendrobium_catenatum_newGene_2377	0.4304	0.232625	0.382503	4.232242	4.239397	4.831122	1.040558	1.164602	0.94287
gene-MA16_Dca026478	0.699727	0.789355	1.183748	0.35111	0.383744	0.175734	0.623493	0.69351	0.755875
gene-MA16_Dca008347	3.705968	3.38296	2.962268	10.938956	10.763415	10.67952	3.827157	4.316832	3.719462
gene-MA16_Dca015242	1.22679	1.09558	1.775598	0	0	0	0	0.215802	0.061102
gene-MA16_Dca006615	5.587772	6.646594	6.658143	10.228878	12.55405	10.028158	20.457363	20.059931	20.134249
gene-MA16_Dca009524	9.60886	7.979972	8.738235	19.731348	15.716341	17.735497	28.604383	29.656607	27.716421
gene-MA16_Dca027324	0.549345	1.495267	0.651959	3.348908	1.724701	1.628218	4.371476	5.782018	4.584309
gene-MA16_Dca020220	3.605957	4.068065	4.054343	5.594918	5.756899	6.71016	10.263142	10.198313	11.70295
gene-MA16_Dca020226	88.492393	87.899078	88.568367	12.662827	12.64701	10.436783	19.891563	19.056377	20.133492
gene-MA16_Dca026750	5.155163	3.77771	4.946677	0.057684	0.045686	0.174401	0.054959	0.073082	0
gene-MA16_Dca026758	92.767822	77.537949	99.263054	36.087383	69.050613	23.212149	35.825897	43.743557	37.281525
Dendrobium_catenatum_newGene_8859	4.758147	2.822202	5.958218	5.121674	3.519044	4.464083	1.326965	1.875058	2.24793
gene-MA16_Dca020895	6.274096	7.973536	5.000211	17.550539	19.781332	16.538702	20.126141	17.386383	16.726572
gene-MA16_Dca006141	30.055454	28.803637	30.582195	64.322571	56.948776	61.108585	92.477356	76.463478	80.073662
gene-MA16_Dca001497	1.740569	2.339806	2.026979	0.367746	0.330533	0.750181	0.481934	0.615116	1.195725

gene-MA16_Dca016402	11.58076	11.984859	10.855188	6.413137	5.288515	5.2822	11.071782	11.584668	9.13138
gene-MA16_Dca016403	252.743164	242.514542	245.226257	129.883163	125.184616	126.113197	165.073227	163.484634	166.081543
gene-MA16_Dca002120	213.011734	197.26268	210.375687	1087.3667	1106.48547	1095.28796	1110.86353	1112.11426	1054.77502
gene-MA16_Dca011999	1.085153	1.659847	1.132041	0.323927	0.729264	0.586856	0.749403	0.24362	0.238076
Dendrobium_catenatum_newGene_2573	0.481139	0.449209	0.489074	0.664066	0.888036	0.848004	1.081435	1.131105	1.202906
gene-MA16_Dca013831	44.261166	40.856445	42.658329	13.093921	12.586858	14.696838	14.298408	12.661346	13.440297
gene-MA16_Dca023421	18.111166	17.138044	18.257439	9.991678	9.229102	10.245271	8.376632	8.387672	7.599804
gene-MA16_Dca019960	1.025919	1.148127	1.275148	2.563444	2.841049	2.518228	2.961706	2.706289	2.85735
gene-MA16_Dca010560	21.220621	18.489174	19.120487	0.964356	0.37428	0.412298	0.993838	1.025174	0.70564
gene-MA16_Dca000221	8.555229	9.003831	10.067516	0.977745	1.119383	1.154479	1.604931	1.788037	1.260812
gene-MA16_Dca000220	9.190623	8.928535	7.289249	14.77621	14.968014	14.422637	31.971764	33.459248	32.261463
gene-MA16_Dca023427	28.283545	26.404982	33.767082	2.805453	3.247954	2.863231	2.366801	2.221046	0.73583
gene-MA16_Dca000224	9.300261	9.121093	9.543278	31.97925	29.104784	28.284006	42.799763	37.340809	39.671219
Dendrobium_catenatum_newGene_10793	0.707198	0.608136	0.800393	0.089498	0.350906	0.208099	0.324061	0.348233	0.227913
gene-MA16_Dca017103	0.812609	1.220704	1.156912	0	0	0.022228	0.0286	0	0
gene-MA16_Dca022561	1.933794	2.14454	1.523724	0.910057	0.883154	0.83847	0.749771	1.684708	1.049011
gene-MA16_Dca018170	10.621435	10.993655	11.321788	6.587258	5.663874	5.225405	3.560233	2.896431	3.253003
gene-MA16_Dca025519	119.215965	124.782318	122.888	58.094002	56.300117	53.403099	53.112728	51.430264	51.599705
gene-MA16_Dca025517	6.607812	6.741122	6.738625	2.38197	2.491868	2.940369	12.181029	11.543315	14.018497
gene-MA16_Dca025514	136.79454	140.62915	134.790283	13.155588	12.437669	11.520442	33.867683	32.958122	37.472115
gene-MA16_Dca017815	20.249241	18.676661	16.571325	12.843713	9.699461	13.106793	11.535027	7.913401	7.91352
gene-MA16_Dca018793	1.558772	1.302735	3.052169	0.731633	0.872994	0.820554	0.366174	0.108944	0.381691
gene-MA16_Dca015418	108.755501	103.797974	108.462288	55.43922	56.350471	55.026642	71.73391	65.875656	71.914062
gene-MA16_Dca015415	2.555256	1.995716	2.56325	6.684635	6.336207	7.330087	7.639754	5.406802	6.702245
gene-MA16_Dca015413	341.628265	337.375793	352.59549	317.647369	310.881348	312.190948	117.124001	113.845108	111.16198
gene-MA16_Dca027821	2.759716	2.6625	2.940537	3.294935	4.456172	3.014166	6.016733	5.678893	8.759887
gene-MA16_Dca001866	58.196823	63.813133	74.737953	22.304993	19.019548	24.286823	12.339294	14.303495	9.02748
gene-MA16_Dca005377	51.466057	49.99511	53.490196	17.882328	19.031988	19.319832	10.844859	11.464055	6.80882

gene-MA16_Dca005372	2.452891	2.08035	3.224102	3.979155	4.736447	4.96666	5.558423	5.382217	7.774683
gene-MA16_Dca005373	73.718773	85.784233	79.569252	20.828077	21.09918	20.748833	14.509983	11.591121	16.3216
gene-MA16_Dca001281	2.841213	2.325817	3.356121	4.364085	2.291472	2.463334	6.172465	8.855696	6.063423
gene-MA16_Dca008559	0.15553	0.087517	0.091243	1.76841	1.61549	1.414506	0.802749	1.591424	1.261803
gene-MA16_Dca001133	22.305557	24.396408	21.946804	12.320278	13.107605	11.748317	44.448971	35.876247	36.741192
gene-MA16_Dca001134	152.673523	152.900253	150.429459	17.051874	17.318264	19.793001	21.892338	21.430241	23.509729
gene-MA16_Dca026153	1.551211	1.714196	1.093517	3.720819	5.428409	3.540354	3.738142	4.996802	4.438926
gene-MA16_Dca026150	0.988681	2.004289	1.327616	0.717789	0.58869	0.653665	0.994714	0.384339	0.960956
gene-MA16_Dca000424	61.210899	63.011063	58.055523	16.430117	15.139533	14.032263	13.850997	11.895244	12.087526
gene-MA16_Dca016236	67.749977	70.463326	67.440659	5.369876	4.857898	4.612595	5.106042	5.672922	4.262838
gene-MA16_Dca010967	0.311754	0.803088	0.733862	1.429555	1.190265	0.94071	2.107182	2.56156	1.701384
gene-MA16_Dca010962	29.033043	30.727234	31.039978	20.045254	18.152132	20.562241	11.333124	11.986771	10.927546
gene-MA16_Dca023019	252.857193	247.958496	238.971405	110.824005	97.610489	106.781006	90.529358	81.706215	86.129814
gene-MA16_Dca023018	32.765442	34.209709	36.083084	16.156961	14.341851	17.120964	13.820833	10.428694	10.333119
gene-MA16_Dca028045	0.039759	0.04932	0	0.020053	0	0	0.616017	0.601242	0.547373
gene-MA16_Dca026225	16.551668	22.147516	18.440168	47.51099	49.998985	47.427063	90.822914	87.901459	86.377457
gene-MA16_Dca026226	732.121155	725.705872	713.972107	2287.13843	2149.7124	2287.51245	2520.51953	2434.80737	2419.03955
gene-MA16_Dca028599	1.130994	1.216531	1.38308	1.810025	2.087331	1.723135	2.591633	2.971214	3.386519
Dendrobium_catenatum_newGene_11016	1.252285	1.210948	1.255083	1.180876	0.514931	0.505234	0	0.292206	0.146591
gene-MA16_Dca024482	1.693079	3.007576	3.005231	3.017795	2.776966	2.593973	5.48022	6.826403	5.521121
gene-MA16_Dca020679	0.431099	0.146873	0.342135	0.92301	0.594317	0.814393	0.387949	0.354122	0.4035
gene-MA16_Dca020673	12.64875	12.591898	12.017243	1.464268	1.335179	1.580311	0.610737	0.929808	0.896211
gene-MA16_Dca020677	34.94939	30.560076	29.316551	3.570502	4.564204	4.185687	3.08377	2.913349	3.228297
gene-MA16_Dca015926	106.913788	115.377235	118.467857	62.63216	56.939045	57.166634	70.947235	66.291756	69.773048
gene-MA16_Dca011208	7.528618	7.470519	8.454829	14.011753	15.01071	14.604138	28.443186	28.723366	27.29468
Dendrobium_catenatum_newGene_10739	0.332822	0.713664	0.485098	1.703595	1.417276	1.478265	1.994323	2.314963	1.387776
gene-MA16_Dca026142	10.446222	11.446383	10.678606	4.402612	3.631526	3.541536	4.417106	3.940783	3.16315
Dendrobium_catenatum_newGene_819	6.575571	6.501811	6.000533	2.180041	1.971238	1.779161	2.526868	2.571536	2.532628

gene-MA16_Dca018333	949.28241	919.058289	923.979431	250.154297	241.308472	244.920181	193.833237	156.229111	179.671814
gene-MA16_Dca019073	24.769545	24.162767	26.211189	17.559704	15.553523	17.747362	12.695188	12.069632	12.38583
gene-MA16_Dca005928	2.375874	2.648099	2.72462	1.902656	1.125716	1.877238	5.236066	3.441818	4.637329
gene-MA16_Dca021260	35.285572	32.538055	34.15794	3.707411	2.812499	2.701488	2.635644	3.147276	2.616477
gene-MA16_Dca007388	0.842866	0.557244	1.053967	0.773456	1.590773	1.530478	1.730132	1.648091	2.297564
Dendrobium_catenatum_newGene_7375	0.443556	0.705	0.251015	1.584599	1.626091	1.476466	1.662995	1.314977	1.18389
gene-MA16_Dca007654	8.49372	7.068706	9.47646	4.537938	4.572447	2.70239	3.895686	3.15133	3.32518
gene-MA16_Dca008747	1.47208	1.5837	1.664395	2.929284	2.160835	2.172568	3.690018	3.623721	3.496614
Dendrobium_catenatum_newGene_1754	0.469614	0.51232	0.586188	0.518359	0.738069	0.78545	1.056576	0.883241	1.707001
gene-MA16_Dca011044	12.787799	10.125554	11.611502	1.138126	0.821369	0.472667	2.316483	1.387914	1.54235
gene-MA16_Dca013576	171.831009	140.965637	150.379776	130.631073	140.780258	121.043755	67.722885	78.382835	74.780838
gene-MA16_Dca011591	69.947906	75.687218	70.434029	7.597785	10.085662	7.609946	14.245523	13.647964	13.540539
gene-MA16_Dca016061	5.9491	5.544788	5.939426	2.677908	2.246278	2.799646	4.50927	3.917487	2.921925
gene-MA16_Dca023603	46.724915	52.466545	49.914417	85.21254	87.205338	79.87355	31.551378	33.91391	30.300928
gene-MA16_Dca023605	7.04007	5.958485	5.946958	1.023484	1.094924	1.222636	0.57966	0.697069	0.914032
Dendrobium_catenatum_newGene_3399	0.97989	0.192551	0.29963	0.675704	0.417018	0.289644	2.290154	1.502521	2.000228
gene-MA16_Dca011865	0.999458	1.406852	0.754376	0.318562	0.335804	0.494193	0.378839	0.882649	0.477073
gene-MA16_Dca021710	13.199978	14.277297	14.276307	4.981441	5.743976	4.79753	4.792688	4.533809	4.6699
gene-MA16_Dca003702	72.617149	69.070999	64.962128	57.408085	51.790691	48.643749	32.186615	29.978544	35.534546
gene-MA16_Dca001418	2.944957	3.256211	4.94187	2.883372	2.514447	2.071965	0.697472	1.192572	0.342643
gene-MA16_Dca001413	16.462688	18.65897	16.779016	5.114823	4.55347	4.873744	5.782043	6.037303	6.274166
gene-MA16_Dca018262	6.782475	6.154939	5.783067	2.439012	1.579098	2.400461	3.073347	2.657521	3.32066
gene-MA16_Dca018264	2.684251	2.852423	3.773098	0.69853	0.454381	0.568004	1.217218	1.43916	1.361397
gene-MA16_Dca018267	10.067213	10.982387	10.392176	3.67345	3.288351	3.5508	4.771956	3.700141	4.796836
gene-MA16_Dca018268	1.806615	1.4158	1.057949	0.140906	0.607275	0.856754	0.612654	0.932538	0.91882
gene-MA16_Dca003839	57.858212	59.432716	56.597538	29.916237	29.432039	29.93437	24.993193	29.908522	27.814375
Dendrobium_catenatum_newGene_1289	11.46326	9.697164	12.237211	4.357004	4.772669	4.066924	11.769823	10.864467	10.982005
Dendrobium_catenatum_newGene_3628	2.968906	2.527402	2.688195	16.039453	14.915609	15.938774	4.414106	5.346712	4.47234

gene-MA16_Dca005063	16.587864	19.771488	19.91753	10.938371	10.826444	10.077934	8.853906	8.162964	9.942122
Dendrobium_catenatum_newGene_9	0.507018	0.676081	0.821496	0.616525	1.055825	3.305794	3.591482	0.777701	3.031112
gene-MA16_Dca008463	2.823394	3.64259	4.533008	4.72086	3.613727	4.610604	9.675799	10.155279	10.833853
gene-MA16_Dca008467	12.975374	11.85455	10.99712	5.320559	4.272382	5.460196	12.021768	11.5811	13.198703
gene-MA16_Dca008465	4.867674	3.613553	4.838161	0.742188	1.076436	0.861793	1.747522	1.027289	0.964427
gene-MA16_Dca008469	3.413318	3.348155	3.423125	5.629082	4.617532	5.176291	18.346773	14.700353	16.24041
gene-MA16_Dca011651	6.865251	6.806225	7.524023	3.366661	3.214192	3.215873	4.194395	5.868392	4.942164
Dendrobium_catenatum_newGene_10545	7.155502	7.058623	6.904812	4.267537	4.454436	4.488244	2.962741	3.518232	2.887952
gene-MA16_Dca028284	16.185398	16.751328	14.964081	1.058247	1.276956	0.917177	4.209849	3.022573	4.207426
gene-MA16_Dca028281	15.134158	13.158403	16.324169	12.414881	13.008434	11.10838	6.157328	5.516811	7.217138
gene-MA16_Dca022875	81.840263	85.622284	89.019943	50.12574	41.428089	38.459045	63.761314	58.804005	61.62558
gene-MA16_Dca023476	16.232174	17.999943	19.471254	8.336483	6.235082	9.327372	5.539313	4.822375	5.927828
gene-MA16_Dca018713	98.44796	100.338547	96.388618	51.516888	55.801174	53.040237	52.841179	48.385471	52.948338
gene-MA16_Dca026641	18.126915	18.062618	17.669073	9.821965	9.279745	9.473364	14.689713	17.086878	17.212072
gene-MA16_Dca026640	6.971038	6.550737	7.855937	2.836335	3.859008	3.4589	4.345571	3.332228	3.045506
gene-MA16_Dca015496	6.708527	4.65718	5.577894	9.266246	9.448975	10.255515	14.430096	13.533048	13.2908
gene-MA16_Dca029217	9.270505	6.633779	6.882226	3.463231	4.500679	2.556904	3.211625	3.992568	2.185001
gene-MA16_Dca021690	37.139637	36.383545	29.478867	41.204185	43.449707	44.172371	20.227413	20.686943	20.065033
gene-MA16_Dca020348	96.302498	97.565369	94.993881	43.87727	36.576603	40.309521	39.379536	33.739708	37.322411
gene-MA16_Dca020342	0.645446	0.329599	0.53921	3.482883	3.232454	4.022128	2.250903	2.07943	1.912197
gene-MA16_Dca021962	98.78904	88.71772	98.980515	34.483875	34.183754	37.870995	24.056789	22.49024	22.358814
gene-MA16_Dca006778	2.368168	2.446516	2.064425	8.591846	7.436104	7.934191	16.012934	14.987633	14.607403
Dendrobium_catenatum_newGene_9917	0.616765	0.89495	1.098056	1.890943	1.932741	2.249655	1.551087	2.137291	1.612918
gene-MA16_Dca004662	23.40205	25.370775	23.663509	11.291018	12.076785	11.782909	15.394571	15.033395	14.660193
gene-MA16_Dca003320	7.116269	7.93994	6.503732	1.417706	1.439374	1.851343	1.162248	1.482587	1.589351
gene-MA16_Dca003119	70.531548	75.579468	74.464622	202.231522	212.517212	191.724167	314.640289	289.335815	287.670654
Dendrobium_catenatum_newGene_9135	1.311562	0.993969	1.651445	0.877758	0.730269	0.652204	0.900859	0.342892	0.599881
gene-MA16_Dca009019	5.358969	4.276191	3.15156	2.56809	2.173584	1.830581	14.573848	11.10007	6.949927

gene-MA16_Dca009014	6.444288	6.092557	4.980994	13.239332	12.696191	15.255171	20.458738	18.65239	18.93742
gene-MA16_Dca009017	55.611916	53.157707	52.630802	21.66827	21.046682	25.834688	14.878572	14.944002	14.913679
gene-MA16_Dca009012	23.901239	26.215778	24.359667	9.540107	10.341967	11.361591	6.190558	8.251167	7.869957
gene-MA16_Dca007145	31.471151	32.662968	30.67379	17.236904	15.348781	16.227909	17.950165	18.149025	17.262167
gene-MA16_Dca007147	93.643349	95.968933	94.882828	29.573528	29.61504	30.645018	22.165922	24.535528	23.383253
gene-MA16_Dca008052	30.317226	29.057217	26.885494	15.048295	16.079865	15.16668	14.830524	12.299467	11.774773
gene-MA16_Dca008051	28.888678	30.696463	29.840031	14.494408	13.761619	14.883574	9.866388	11.07675	10.119022
gene-MA16_Dca009907	2.312975	0.665284	0.734661	1.678398	3.163344	2.542322	2.936614	3.742344	4.639638
gene-MA16_Dca009904	1.325097	1.362832	1.033323	2.495892	1.255375	1.969444	2.929507	4.088364	4.732332
gene-MA16_Dca009908	14.54183	15.40506	14.067255	8.21646	7.647273	7.796744	7.317972	6.55442	5.899791
gene-MA16_Dca010738	1.250884	2.207764	2.084664	2.669716	2.063452	1.910114	3.911587	3.983621	4.413041
gene-MA16_Dca011281	52.725166	52.671528	52.283314	9.412738	8.397141	8.853532	11.767727	10.766691	10.790006
gene-MA16_Dca000308	19.614595	23.716368	22.55022	22.912096	23.821207	28.609646	10.90484	10.489718	11.916895
gene-MA16_Dca016888	603.947876	602.379395	567.983154	365.997009	349.325043	349.609497	252.212051	260.274017	266.984985
gene-MA16_Dca012399	1.260387	0.66705	0.805497	2.809612	3.058767	3.332803	2.939306	3.125373	4.109109
gene-MA16_Dca012394	1.641442	2.07926	2.106665	3.255493	3.210859	2.271854	7.047347	6.457549	7.108379
gene-MA16_Dca027329	3.928813	3.215216	3.360544	4.223713	4.410557	4.528058	7.862309	9.515631	9.725063
gene-MA16_Dca025902	9.575163	10.0739	10.765448	5.261813	4.164512	5.767371	6.277825	5.466222	4.735012
gene-MA16_Dca013113	12.825636	12.534278	13.64326	2.299388	1.85792	2.090989	2.775342	2.843372	2.796179
gene-MA16_Dca013112	10.179093	10.143894	9.482707	3.131333	3.253405	2.791494	1.693021	1.431994	1.876942
gene-MA16_Dca013117	0.889451	1.220706	1.63139	0.195738	0.690739	1.396405	3.956519	3.702786	4.074036
gene-MA16_Dca025659	23.733147	25.920965	21.215134	5.301977	5.685905	7.1819	19.955221	20.11882	21.508366
gene-MA16_Dca027810	319.561371	269.869354	285.340027	85.321266	218.817795	96.387672	87.512093	128.427231	133.51384
gene-MA16_Dca018036	379.343689	348.991241	485.741669	129.260513	121.112648	81.246109	125.623642	117.35244	222.982788
gene-MA16_Dca014247	112.899147	116.95784	103.843445	41.779541	42.84581	45.130634	45.113144	44.344158	45.385796
gene-MA16_Dca018695	8.693293	9.320556	9.748586	15.850699	15.10154	19.721375	6.160647	4.71144	6.503725
gene-MA16_Dca018694	7.470375	9.679241	8.963505	26.80444	26.612316	29.780893	10.026383	12.945946	10.738317
gene-MA16_Dca018690	122.054375	117.000046	117.822777	29.431305	25.771423	27.820192	31.688051	32.235428	33.772083

gene-MA16_Dca018699	2.984626	3.437412	4.665084	15.269617	16.658665	14.191979	7.407703	6.1391	6.538416
gene-MA16_Dca018962	0.324192	0.813232	1.204233	2.667842	2.621475	2.140319	3.982478	2.435743	6.12093
gene-MA16_Dca015517	1.044936	1.099827	1.018943	0.280337	0.113146	0.402044	0.422906	0.215718	0.153416
gene-MA16_Dca020137	65.369835	67.24054	70.539261	143.933868	129.225494	133.892746	185.997894	179.073044	185.989563
gene-MA16_Dca020134	6.410837	4.791124	3.937508	0.11044	0.152803	0	0.53964	0.187866	0.51566
gene-MA16_Dca007306	5.090106	7.669803	5.183772	4.530561	4.407712	4.467977	15.143767	12.177982	12.055692
gene-MA16_Dca007305	16.852285	12.895952	14.482527	5.47746	6.018644	7.066004	3.283035	3.610683	3.388945
gene-MA16_Dca007304	55.173328	55.001282	48.157104	37.069012	33.599369	34.241894	28.782585	26.989313	25.244812
gene-MA16_Dca007303	32.925018	30.091869	30.274574	17.462292	14.192596	13.671166	19.818018	19.51502	18.253981
gene-MA16_Dca008217	129.716629	124.100121	118.952477	51.763607	50.880573	55.245792	113.667091	118.04483	122.454636
gene-MA16_Dca007895	68.691536	66.566673	68.917862	26.396496	25.536783	25.545631	27.41165	26.810568	26.500223
gene-MA16_Dca005796	53.729137	57.885994	52.946007	25.173546	24.193537	25.971106	23.510866	22.833662	22.870302
gene-MA16_Dca005797	1.935967	1.94911	2.315183	0.553289	2.124795	1.296108	0.687286	1.396574	0.663569
gene-MA16_Dca005793	18.021198	17.06636	18.036459	9.044072	10.023428	9.56897	6.07549	6.570643	6.647848
gene-MA16_Dca005791	2.997373	3.251534	3.347579	5.339863	4.267497	4.424253	6.196261	7.542376	6.895044
gene-MA16_Dca000676	123.972763	112.215454	113.2416	46.777443	45.078957	44.029537	61.312096	59.011147	59.819374
gene-MA16_Dca000675	24.804773	23.350206	24.659071	9.658016	12.958136	12.398504	15.849529	18.529081	12.292338
gene-MA16_Dca006855	72.067513	76.789719	77.353111	46.475639	44.889961	46.770905	38.05228	34.509594	37.062218
gene-MA16_Dca016579	3.927036	3.585207	4.45588	5.702477	3.953971	5.709353	8.842226	9.795663	7.875183
gene-MA16_Dca026306	12.795215	13.027137	13.577705	7.178463	6.513945	7.083001	7.551565	9.166694	8.691971
gene-MA16_Dca022154	1.643467	1.488676	2.170542	3.817102	3.313264	3.733408	4.165439	4.045149	3.605567
gene-MA16_Dca008152	235.053497	229.51738	238.150024	56.468521	64.353928	65.110733	64.468056	65.831375	55.049206
gene-MA16_Dca019893	2.417095	1.780635	1.998387	5.202117	4.624671	4.094976	3.467844	3.846714	3.656248
gene-MA16_Dca011361	0.621419	0.596667	0.564959	1.117505	0.744209	0.723264	2.063568	2.918485	2.137708
gene-MA16_Dca023364	0.653835	0.763858	0.672258	1.453218	1.14924	1.092451	1.680768	1.875719	2.34305
gene-MA16_Dca014072	852.050781	824.974731	829.908936	372.686005	361.678955	364.883698	549.329529	522.794861	527.484009
gene-MA16_Dca014074	0.766962	0.542229	0.739591	0.173202	0.250485	0.286697	0.253253	0.217921	0.205856
gene-MA16_Dca021182	2.191517	2.481121	1.845765	0.672948	0.440921	0.331353	0.832813	0.533257	0.551843

gene-MA16_Dca021189	32.753418	31.811121	32.436485	15.038392	16.027367	14.482791	17.641647	16.953897	17.053555
Dendrobium_catenatum_newGene_3418	10.794722	15.722825	13.87149	13.892771	15.663375	11.847767	34.942154	26.130512	29.649212
gene-MA16_Dca015041	4.424187	4.765575	3.723241	0.73693	1.541375	2.345964	0.62881	0.622483	0
gene-MA16_Dca006641	1.321687	1.069549	1.201557	2.650806	3.246193	2.995661	5.714129	6.023852	7.675829
gene-MA16_Dca002303	193.611008	192.071198	198.910782	21.109529	21.88006	25.802702	16.150877	14.323799	15.352753
Dendrobium_catenatum_newGene_3840	0.98879	0.424067	0.890942	0.634041	1.132045	0.700076	2.712863	2.176581	2.671184
Dendrobium_catenatum_newGene_3846	1.09898	1.259354	1.204224	1.640886	1.865328	1.98814	2.354686	2.801543	2.47648
gene-MA16_Dca002484	14.409959	14.785685	14.731377	4.032681	7.109028	6.037743	8.157214	9.179873	8.459051
Dendrobium_catenatum_newGene_5733	5.525897	6.956408	7.355767	4.478266	5.72226	5.3532	2.311739	2.423181	2.074062
gene-MA16_Dca009701	0.563581	0.45037	0.319104	0.980701	0.748576	0.997428	1.545613	1.988775	1.638842
gene-MA16_Dca012530	0.275096	0.253203	0.367537	0.390129	0.099232	0.220816	2.428449	0.911798	1.40118
gene-MA16_Dca026539	10.193298	10.366797	9.586534	9.690078	9.699807	8.487202	3.982047	3.860395	4.666499
gene-MA16_Dca011948	8.344669	8.869973	7.692373	19.172443	21.241625	17.460951	16.015345	15.916806	15.551606
gene-MA16_Dca014277	898.657959	903.099731	904.802856	229.145462	228.832199	212.11557	334.866241	311.345734	305.814026
gene-MA16_Dca025374	25.811794	21.362949	25.060007	8.365833	8.742832	9.825624	7.151851	7.494291	7.509059
gene-MA16_Dca025379	16.230112	17.227327	20.275394	13.673971	13.135267	13.745014	43.997791	44.726173	40.177563
gene-MA16_Dca019447	8.42485	8.4992	6.536578	17.765064	17.324612	16.802505	25.733583	28.89806	28.876265
gene-MA16_Dca024023	18.542877	21.658182	19.100195	15.07841	14.246099	13.478417	9.327307	8.725049	9.009778
gene-MA16_Dca024029	2.440618	2.715139	2.701225	3.744281	3.552038	4.290771	5.321865	5.471243	6.001993
gene-MA16_Dca027610	0.964627	0.745197	0.479478	0.285862	0.113176	0.323543	1.702698	1.415548	1.094452
gene-MA16_Dca027619	1.663582	2.300179	2.36713	1.22344	1.929946	1.18052	0.750313	0.417522	0.999412
gene-MA16_Dca014390	36.096188	34.127811	34.173271	16.423399	17.038223	15.177027	18.754923	18.920315	21.242676
gene-MA16_Dca017203	0.79032	1.690363	1.370809	6.791312	4.0301	5.460779	3.320572	3.84	5.244882
gene-MA16_Dca017200	349.582245	340.890442	339.972107	81.882446	80.603752	77.35778	82.516029	80.380531	73.617699
gene-MA16_Dca007497	6.215485	8.212347	8.678372	0.986791	1.093551	1.859781	0	0.251969	0.347146
gene-MA16_Dca007722	532.826843	533.483887	555.722107	155.870682	149.078232	152.267883	128.214905	126.144424	124.317307
gene-MA16_Dca005385	0.471585	0.127881	0.197533	0.824244	1.243644	0.948055	3.073376	2.537963	2.987296
gene-MA16_Dca005656	85.55835	85.616943	81.554008	40.670517	37.365097	37.563427	28.638418	26.883448	27.291018

gene-MA16_Dca008584	1.190022	0.84879	1.378798	1.957494	1.715355	1.95642	2.463129	3.839568	4.013127
gene-MA16_Dca000481	1.058425	0.719235	0.338908	3.144359	2.946942	3.833131	1.641676	1.623795	1.865176
gene-MA16_Dca000380	10.371916	8.552205	9.512497	6.111792	6.483858	6.629917	4.659355	3.842952	4.469344
gene-MA16_Dca010400	1.808502	1.180245	1.329434	2.048101	2.39764	2.258643	5.305511	4.962591	4.122353
gene-MA16_Dca020815	4.899353	5.062459	6.613253	14.846392	11.995029	13.671292	14.051229	14.24761	13.064181
gene-MA16_Dca020810	30.858473	33.947319	35.231197	57.982098	55.044964	58.73111	85.577415	85.662941	79.383347
gene-MA16_Dca020812	4.337313	4.177366	6.073018	6.091506	9.750486	8.989821	10.595086	13.265145	9.621828
gene-MA16_Dca001727	1.782887	2.764784	2.563348	4.094702	3.994733	4.670829	5.035712	5.742408	6.013641
gene-MA16_Dca000966	5.319585	2.579937	2.665336	1.861241	2.676761	1.711015	1.846832	1.408442	0.496161
gene-MA16_Dca011463	21.140387	21.044851	21.395021	10.144481	10.316228	10.234278	10.863132	11.32523	10.765289
gene-MA16_Dca011462	12.064708	12.777987	11.940871	0.907838	0.575967	0.429539	1.723881	0.554943	1.095164
gene-MA16_Dca013198	27.584528	29.636742	28.954584	33.522583	28.836208	31.083294	61.435516	61.54969	60.916992
gene-MA16_Dca002524	5.03904	4.427201	4.503254	8.386313	5.358562	6.641744	11.539202	11.379706	10.257733
gene-MA16_Dca002526	559.716858	565.232178	569.973511	738.286072	746.131165	743.04248	347.9328	352.801605	332.146545
gene-MA16_Dca025941	22.38105	16.262701	30.109186	8.54624	13.561095	10.453119	17.18297	13.927875	17.81601
Dendrobium_catenatum_newGene_11594	2.656078	3.008658	2.624823	0.619317	0.870847	1.018139	1.004305	0.521821	0.968096
gene-MA16_Dca017441	1.610715	1.553366	1.453351	2.248899	1.605207	1.413113	4.801464	2.48858	2.817476
gene-MA16_Dca018618	3.161424	3.147516	3.497315	1.643412	1.16414	0.944072	3.239728	3.735869	3.221155
gene-MA16_Dca018612	38.53109	36.212391	35.823475	60.867661	59.132523	64.206078	80.399658	77.054359	75.871513
gene-MA16_Dca017996	8.017375	6.897339	4.720355	10.657601	9.134937	7.974691	21.520962	18.640364	19.71339
gene-MA16_Dca017990	133.356079	122.609894	133.012543	49.739906	42.664711	40.81015	45.636234	42.878704	38.865414
gene-MA16_Dca015597	4.775516	3.260525	3.753537	6.318737	6.066564	6.138925	7.710476	10.448767	7.151751
gene-MA16_Dca024727	22.31003	22.09791	22.167137	6.76254	6.848701	6.020261	8.494752	6.340964	4.787319
gene-MA16_Dca024723	5.439923	4.171961	4.71359	0.160004	1.708423	0.475431	0	0	0
gene-MA16_Dca024720	1.487278	1.065509	1.011148	3.340936	3.153025	4.586792	2.430288	2.175185	1.458786
Dendrobium_catenatum_newGene_3731	0.653676	0.345157	1.025134	2.24899	3.195064	1.959064	1.216854	1.785599	0.975524
gene-MA16_Dca025238	0.098165	0.406165	0.219175	0.073797	0.032943	0.220104	0.478846	0.555555	0.792953
gene-MA16_Dca005808	0.216872	0.159731	0.110571	0.667187	0.811852	0.697396	0.953424	1.07273	1.190197

Dendrobium_catenatum_newGene_768	4.019314	5.14575	4.334263	2.14876	2.284604	1.350915	1.022033	1.81158	0.92983
gene-MA16_Dca016167	7.752482	10.148689	9.98968	4.355995	4.828404	2.974681	6.293858	6.55318	5.434861
gene-MA16_Dca008007	4.345789	4.458572	4.354389	5.109112	6.503052	10.808836	7.998702	10.717733	12.155592
Dendrobium_catenatum_newGene_1042	15.930976	19.364831	21.540021	6.218424	6.152319	6.154141	6.840532	8.999627	10.474699
Dendrobium_catenatum_newGene_2107	0.204509	0.338794	0.641733	0.659027	1.17951	0.891154	1.442508	0.574563	1.783296
gene-MA16_Dca002050	11.232977	10.966715	10.60883	0.309893	0.20808	0.354941	0.280281	0.559429	0.530668
gene-MA16_Dca009354	2.18757	2.900082	2.316392	0.187393	0.411806	0.399318	0	0.09721	0
gene-MA16_Dca009355	69.176628	71.145638	68.07827	37.798859	38.776108	35.856441	23.422266	21.958534	21.433397
gene-MA16_Dca020852	2.715713	1.972909	2.622575	1.495724	0.951771	1.376289	0.969714	0.769966	0.57124
Dendrobium_catenatum_newGene_2664	39.678501	41.349209	42.175476	20.308483	17.476192	18.157053	13.805913	15.382375	10.656768
gene-MA16_Dca024524	1.898959	1.032146	1.782915	0.334253	0.279187	0	0.156832	0.419364	0.408701
Dendrobium_catenatum_newGene_13970	0.545893	1.513429	0.844921	3.050728	5.670893	6.446211	5.499332	3.929718	0.223764
Dendrobium_catenatum_newGene_5979	1.358165	1.947503	1.382445	0.909196	0.85085	1.116776	0.288836	0.365457	0.33107
Dendrobium_catenatum_newGene_5970	5.384611	4.710621	5.86324	8.873344	8.497638	9.660842	10.800802	10.281336	12.693626
gene-MA16_Dca019812	887.424622	915.425842	890.745361	390.320557	380.70932	364.794586	601.830383	634.393066	646.929443
gene-MA16_Dca019813	748.215759	720.500427	710.21228	263.022705	259.22345	274.203156	449.53299	425.255219	424.716248
gene-MA16_Dca026794	51.075684	49.008541	47.393353	94.09774	90.569435	91.93692	225.418091	227.484558	216.179352
gene-MA16_Dca000029	0.15169	0	0.28309	0.685521	0.427011	0.632977	6.159358	6.228363	5.636611
gene-MA16_Dca000028	4.725649	5.180632	3.333565	6.07097	7.28661	6.991711	10.368555	11.368128	9.541744
gene-MA16_Dca012298	24.454439	26.277891	24.295191	12.86046	12.40812	12.947224	15.644504	20.392572	18.024769
gene-MA16_Dca010185	1.554097	0.524951	0.663342	0.630816	0.58534	0.520962	2.569962	1.672819	3.522513
gene-MA16_Dca003598	9.919669	9.372254	9.61075	17.499849	15.391805	17.942038	26.411592	22.928329	25.221962
gene-MA16_Dca003593	2.338643	1.085583	2.294432	3.457264	2.628177	3.214871	8.275218	5.76441	6.277719
gene-MA16_Dca003597	82.768242	78.453423	81.56929	39.288078	41.370819	38.475842	57.491211	51.268074	52.757614
gene-MA16_Dca010182	3.482524	4.074446	4.146635	2.041531	1.628441	1.612704	2.79986	2.487923	3.629059
gene-MA16_Dca023503	2.014002	1.683705	1.987057	1.153468	1.948287	1.288241	7.937744	5.59586	10.255259
gene-MA16_Dca022456	1.235513	1.419089	1.292834	1.934532	1.981085	2.445054	2.844915	2.724367	3.133138
Dendrobium_catenatum_newGene_8707	0.449868	0.207815	0.091125	0.485745	0.890705	0.447184	0.49332	0.841562	0.579367

gene-MA16_Dca021107	67.185791	69.585854	66.72747	81.70089	75.314842	84.823082	16.409086	23.942966	28.450966
gene-MA16_Dca007220	221.346542	227.687469	223.894958	31.430809	30.094379	29.827766	29.637871	28.886993	27.002615
gene-MA16_Dca020767	1.438292	1.436549	1.590937	2.375122	1.618962	1.195091	4.115778	4.193816	3.474206
Dendrobium_catenatum_newGene_8880	2.466138	2.672189	2.227686	0.884712	0.754729	0.923438	0.704779	1.166249	0.718983
gene-MA16_Dca027236	0.759645	0.921167	0.740063	3.208868	1.230677	2.648923	2.958967	3.311887	2.05701
gene-MA16_Dca006171	16.514355	14.567722	13.886663	8.272305	8.135243	8.434796	4.092099	2.887554	3.323155
gene-MA16_Dca006175	19.144567	18.79743	16.90806	7.278165	7.179939	5.851008	7.166083	5.654205	5.077168
gene-MA16_Dca011134	5.32612	4.330866	4.56981	2.306627	2.003102	3.084558	2.437765	2.49059	2.800688
gene-MA16_Dca025263	11.694567	10.70698	12.442569	20.58066	25.883768	22.486567	25.326273	29.684446	25.533237
gene-MA16_Dca005528	115.396606	96.005653	98.524597	12.946404	11.838531	13.732891	21.330143	18.834076	15.345201
gene-MA16_Dca005525	179.35025	164.83754	164.967102	22.286554	21.03647	28.615896	38.127247	37.767097	31.11359
gene-MA16_Dca005524	186.006699	164.301941	181.93718	62.006191	68.422508	63.88398	69.157127	63.243202	59.7215
gene-MA16_Dca005527	533.242188	471.332611	496.993134	51.775402	50.40115	49.231144	75.313469	67.972633	65.807274
gene-MA16_Dca006955	2.743775	2.779238	2.848416	4.118694	4.115356	3.767846	6.239816	6.033001	5.557
Dendrobium_catenatum_newGene_8069	31.394188	30.930319	32.27816	12.085054	11.69854	10.847578	10.940348	11.894461	12.245987
gene-MA16_Dca003207	1.560552	1.527998	2.091428	2.601847	0.967061	0.35623	3.847263	4.141474	4.005361
gene-MA16_Dca008872	3.131532	4.299159	3.443979	7.869205	8.930686	7.893217	13.271404	13.125601	12.718551
gene-MA16_Dca013846	38.068634	36.829529	39.90723	26.132177	24.397907	26.619499	173.891479	161.400848	162.860977
gene-MA16_Dca013842	7.263982	9.309824	9.00397	2.415619	2.802207	1.682025	2.569001	3.120694	3.702202
gene-MA16_Dca014812	311.72525	323.192566	321.484375	139.943314	129.689865	125.238762	61.121605	59.270809	59.460335
gene-MA16_Dca015149	33.248051	32.289589	31.563421	17.492102	15.121733	17.138733	16.741577	16.399807	17.492668
gene-MA16_Dca015147	2.909635	2.832942	3.723447	7.704414	7.555339	8.292742	9.46551	9.675029	8.936082
gene-MA16_Dca015145	0.771377	0.425223	0.262652	4.454967	3.741653	3.867323	0.550255	1.016249	0.273616
gene-MA16_Dca019997	33.750778	27.175594	29.758247	8.540755	5.867078	7.41974	2.671927	2.654626	4.36274
gene-MA16_Dca017178	115.451378	108.773018	103.106804	46.603004	41.752789	45.668026	33.054508	29.921328	25.996323
gene-MA16_Dca017173	19.336533	19.201605	19.150002	9.941567	7.786643	9.114739	6.091285	4.951392	5.207516
gene-MA16_Dca017172	0.163491	0.250523	0.118501	0.366034	0.413622	0.458283	2.468953	1.231857	2.319067
gene-MA16_Dca026079	11.67833	14.044689	11.501451	3.683478	3.936134	4.104624	3.200452	3.759115	2.879843

gene-MA16_Dca016398	1.857871	2.272393	1.574294	0.316343	0.168099	0.153624	0.390886	0.49213	0.766093
gene-MA16_Dca016399	20.326731	21.579302	22.236448	9.042265	10.199821	10.719116	14.475559	13.626364	11.661735
gene-MA16_Dca025523	51.556007	49.706688	52.144615	19.747984	21.01272	22.425045	22.866022	23.944086	21.220274
gene-MA16_Dca025528	60.630783	64.980164	65.992355	3.662153	2.144803	3.532797	10.217051	9.604431	11.107584
gene-MA16_Dca015466	22.579287	22.155016	21.689901	13.532557	13.127431	14.719129	10.252819	9.520087	10.594346
gene-MA16_Dca004944	5.733602	6.336013	8.199449	1.055672	1.386088	0.930826	5.543314	5.607158	4.188497
gene-MA16_Dca004940	0.823815	0.477738	0.893901	1.50088	1.361117	2.100525	1.893633	2.856189	2.296662
gene-MA16_Dca020046	5.72787	6.20252	5.788178	5.734928	5.978353	4.579897	1.627634	1.919508	2.416589
gene-MA16_Dca007414	2.651121	2.816613	2.285018	0.525677	0.875887	0.902798	6.086767	4.570544	5.792255
gene-MA16_Dca001811	248.491318	250.042572	225.427933	75.550278	61.065815	70.823601	36.95546	29.315142	26.168261
gene-MA16_Dca006453	3.068717	3.233343	3.31516	6.622801	6.97361	5.301672	9.827422	8.116958	11.43172
Dendrobium_catenatum_newGene_15067	5.583955	7.517403	9.44493	20.179384	20.367041	18.880051	22.284964	19.857594	19.076054
gene-MA16_Dca008501	29.539108	32.052567	28.571424	11.492794	10.447053	11.472989	11.970285	7.434385	10.254706
gene-MA16_Dca008504	1.787463	1.648157	1.912628	0.752284	0.585974	0.983734	0.805161	0.661424	0.650557
gene-MA16_Dca008507	18.925858	15.41357	17.04962	4.159838	3.593842	2.552166	1.662547	2.389171	2.355934
gene-MA16_Dca004162	3.825505	5.496087	5.007257	11.780788	11.581245	11.092031	10.351626	9.132016	9.486937
gene-MA16_Dca004160	213.039566	208.427307	212.454407	12.106178	9.070713	10.648921	13.432404	13.076633	16.225132
gene-MA16_Dca001102	93.857582	92.125389	90.441376	60.759491	58.68895	62.708088	38.472607	42.273022	38.359936
gene-MA16_Dca001101	75.232384	74.010269	76.266281	6.893085	5.630816	6.107984	8.835839	8.168061	7.642563
gene-MA16_Dca010936	15.280392	15.339925	14.626529	6.858426	6.818599	6.666075	9.725429	9.852855	9.399885
gene-MA16_Dca023046	129.760269	114.275444	121.172607	26.999525	28.544146	31.64991	26.18679	23.58602	22.892223
gene-MA16_Dca012473	0	0.247744	0.028327	0.212407	0.407735	0.294088	1.226492	1.124105	0.88346
gene-MA16_Dca022795	228.969009	251.556656	236.223755	185.268356	175.958466	196.709991	108.38076	130.103104	128.215897
gene-MA16_Dca022799	20.393299	18.454945	17.527811	8.025134	7.132232	2.421449	4.041787	3.483346	2.767312
gene-MA16_Dca009591	1.301757	1.211967	1.725958	2.23915	3.517606	3.235964	4.15936	4.693065	3.503868
gene-MA16_Dca009592	473.672943	455.146271	460.230377	888.869141	905.220886	903.296753	1060.33533	1039.44885	1013.9458
Dendrobium_catenatum_newGene_3223	0.980487	0.738736	0.972262	0.99242	1.854123	0.408185	2.680229	1.955954	1.539524
Dendrobium_catenatum_newGene_451	7.053555	5.970802	5.701611	3.307627	4.699877	4.247851	1.566929	2.570813	4.241997

Dendrobium_catenatum_newGene_14943	2.34905	1.705369	2.60686	2.760422	1.838034	3.539566	15.379344	15.30184	17.106535
gene-MA16_Dca019560	8.013308	4.225912	5.568601	13.277834	11.40656	8.924573	43.398529	41.538612	42.974648
gene-MA16_Dca019562	4.202849	4.079117	5.088237	1.355723	1.123715	1.775101	2.477196	2.344514	2.893087
gene-MA16_Dca019563	160.471008	169.367065	166.929489	40.316147	42.741062	44.099758	49.025311	43.472019	43.851524
gene-MA16_Dca010167	77.285698	74.032234	74.428864	9.238434	8.771581	9.458076	12.063521	11.608277	11.139237
gene-MA16_Dca010166	6.308721	6.100961	6.048888	1.524613	1.566818	1.360393	2.870044	2.42615	2.161471
gene-MA16_Dca020688	6.632674	7.244302	6.769938	9.497178	8.23056	9.738404	16.952627	15.303642	17.336435
gene-MA16_Dca013939	0.194267	0.182994	0.556982	0.515295	0.536146	0.546182	0.871811	1.176281	0.797366
Dendrobium_catenatum_newGene_11813	1.826185	1.33654	1.704167	1.13318	1.221061	1.189812	0.21757	0.484196	0.430265
gene-MA16_Dca019021	0.920623	0.779287	0.913451	0.818195	1.548877	0.698432	6.445184	7.908681	8.422147
gene-MA16_Dca017328	2.772764	2.892	2.396942	7.33218	6.047855	5.944423	6.508593	7.118917	6.733454
gene-MA16_Dca014670	43.47641	43.060452	43.794991	19.549305	19.513361	19.976635	23.562979	23.833181	22.640957
gene-MA16_Dca019040	1.423468	1.534026	1.253029	0.697033	0.526435	0.389426	0.810265	0.468583	0.550382
gene-MA16_Dca019049	3.978786	3.851472	5.844205	5.182283	8.809846	9.19537	9.415807	10.939322	11.114021
gene-MA16_Dca022697	4.568428	4.236438	4.03999	2.385293	0.817767	1.867894	1.420006	1.140743	3.300927
gene-MA16_Dca022690	28.240803	28.764423	24.810625	8.929134	10.624861	11.360116	7.754346	8.341541	6.513264
gene-MA16_Dca026533	3.240096	2.737158	2.075059	1.634938	0.205874	0.799246	1.047403	1.693033	0.223815
gene-MA16_Dca001602	5.521608	4.510112	4.870068	1.928232	1.688653	1.304392	3.142128	2.061867	1.810784
gene-MA16_Dca005113	18.152613	22.314344	20.433121	42.89069	41.8339	45.196766	53.099098	55.822792	52.809742
gene-MA16_Dca010362	4.862666	3.940715	4.351807	2.197182	1.441533	0.947458	2.113658	2.497583	1.956746
gene-MA16_Dca008773	11.288926	10.538422	11.752018	3.843439	5.517171	4.237564	3.364383	3.357075	2.935828
gene-MA16_Dca008771	0.179149	0.243473	0.165401	0	0.22225	0.100788	0.772925	1.089541	0.770707
gene-MA16_Dca012213	31.093159	30.998981	30.342966	11.275664	11.732406	11.37408	21.021063	17.73567	18.84453
gene-MA16_Dca012211	18.877409	17.550228	14.403371	9.996808	8.051886	8.672988	10.712219	11.459189	11.642278
gene-MA16_Dca003516	55.295078	60.812496	55.234333	35.1506	34.857166	38.503807	26.823938	30.908741	31.271729
gene-MA16_Dca003517	0.298891	0.314821	0.439832	0.69404	0.349042	0.838837	2.857539	1.767149	1.662456
gene-MA16_Dca020910	2.455927	2.175433	1.948765	0.079243	0.126464	0.237377	0	0.033876	0
gene-MA16_Dca020914	29.633787	31.682037	29.266249	1.578321	0.700205	0.715633	0.577627	0.571733	0.560269

Dendrobium_catenatum_newGene_15803	127.071617	140.260773	142.7229	61.627811	53.304585	60.718349	111.916725	120.423447	122.784073
gene-MA16_Dca016013	5.213151	4.636063	4.607662	1.991107	2.476514	2.598956	2.534185	2.234238	2.329602
gene-MA16_Dca008434	31.209221	32.307568	31.044704	2.407508	1.765796	1.399499	1.253975	0.774989	0.428936
gene-MA16_Dca026404	0.664593	0.618131	0.505326	2.889944	2.184184	1.806644	3.611903	4.110118	4.74719
gene-MA16_Dca015652	5.293404	5.839169	4.832485	6.88639	6.002416	7.320254	19.354296	17.95225	20.859938
gene-MA16_Dca018550	72.059891	74.786125	79.972382	18.843571	15.392159	17.476072	15.213865	17.534704	15.101478
gene-MA16_Dca014302	117.761246	121.477821	125.368576	50.944759	43.565475	52.168236	62.749382	57.587311	58.991497
gene-MA16_Dca014300	2.131427	1.98257	2.530414	0.433159	0.925935	1.067581	2.963613	2.812089	2.573381
gene-MA16_Dca025173	47.703014	43.174854	48.242371	5.430444	4.796428	6.469054	8.951611	9.742276	8.778501
gene-MA16_Dca020275	1.804589	1.379895	2.455508	0.504014	0.49719	0.396284	1.469779	0.760788	1.303821
gene-MA16_Dca020277	0.228723	0.176163	0.141479	0.535823	0.708175	0.437834	0.728674	0.869642	0.813369
gene-MA16_Dca002474	1.250256	1.175351	0.589512	0.808854	1.474242	1.077039	2.383011	2.407506	2.426568
gene-MA16_Dca026892	655.632141	684.623047	665.584839	294.152008	291.229034	306.368286	210.108566	187.169434	181.23819
gene-MA16_Dca026890	9.08408	8.023445	8.841059	18.525467	19.287216	19.212996	30.457644	30.989496	29.548176
gene-MA16_Dca023910	0.79186	0.699657	1.0803	2.223272	1.931582	1.054652	2.683832	3.345649	2.234621
gene-MA16_Dca020554	6.154253	7.61986	8.877049	12.705159	15.005067	14.329482	15.298503	16.032419	17.791252
gene-MA16_Dca017514	92.602203	94.48362	94.906067	46.94614	44.472137	42.981873	42.707794	39.78508	40.796356
gene-MA16_Dca004256	6.970449	8.110442	8.156381	1.758091	2.375263	2.964234	0.080206	0.276588	0.131353
gene-MA16_Dca004258	18.441154	17.280806	18.262506	6.731085	6.606772	7.041055	4.223835	5.060592	5.730293
gene-MA16_Dca011578	0.473682	1.384397	0.892812	1.863745	2.362208	2.928278	1.265939	1.789797	1.590328
Dendrobium_catenatum_newGene_5226	0.811021	0.516623	0.833404	1.397375	1.473361	1.664697	0.772605	1.145995	0.958445
gene-MA16_Dca004877	544.850769	524.394226	512.61731	78.631096	76.70385	72.713287	70.876648	61.859562	57.786243
gene-MA16_Dca012829	8.238028	9.315829	9.008012	4.572725	3.001802	4.264084	4.925028	6.25777	5.866009
gene-MA16_Dca002849	0.136807	0.064582	0.175591	0.352808	0.07116	0.575246	2.00845	1.638916	1.449564
Dendrobium_catenatum_newGene_3670	70.966054	76.492573	78.117173	16.673395	15.69377	16.962841	17.047425	16.595215	16.336002
gene-MA16_Dca019913	15.566405	14.252094	15.255806	4.158259	4.893451	5.047127	5.962121	7.588264	4.440612
gene-MA16_Dca010533	19.958347	19.037514	20.122768	3.741804	3.255405	2.665926	14.138098	12.734688	11.373866
gene-MA16_Dca014286	23.628922	25.610783	25.626652	14.800207	12.799897	14.246221	10.516511	12.009134	9.206665

gene-MA16_Dca014287	10.466929	11.813188	8.487899	4.466177	4.194758	4.476305	5.210565	5.489786	5.302284
gene-MA16_Dca012007	145.319885	135.779007	126.600128	42.552231	50.43734	51.437305	62.552567	65.193008	60.076
gene-MA16_Dca012004	123.493698	132.677109	119.679939	32.363567	31.821388	33.780457	30.835548	27.2861	26.516914
gene-MA16_Dca012005	2.136492	1.317793	2.569156	1.96465	2.726016	1.617659	3.802835	8.07404	4.777398
gene-MA16_Dca012009	2.273502	2.190506	1.861911	6.372496	5.624137	6.877514	10.507814	10.890984	10.109139
gene-MA16_Dca003497	14.071625	14.693346	14.372046	8.829383	8.859178	10.037336	7.424939	7.540785	6.990135
gene-MA16_Dca003496	5.399819	6.748681	5.83302	21.558374	22.323925	19.941719	7.402001	5.969971	4.338418
gene-MA16_Dca003490	6.447836	7.273532	6.465577	1.311658	1.373633	1.387332	1.716136	1.841815	1.784604
Dendrobium_catenatum_newGene_16265	2.122388	2.16225	2.089203	1.761647	2.14582	1.279894	0.552622	0.803301	0.62394
gene-MA16_Dca009201	32.697166	29.579685	30.776131	4.979127	4.278012	4.207698	4.7615	4.370044	4.996428
gene-MA16_Dca012583	14.066031	12.091744	12.63749	7.92166	5.752188	7.373288	6.59329	5.36708	6.037907
gene-MA16_Dca012581	6.560632	6.859134	4.294645	0	0	0.789125	0.487853	0.26702	0.216664
gene-MA16_Dca012587	112.292778	104.684441	101.954102	37.983269	36.880558	39.311111	41.802956	40.055874	36.359154
gene-MA16_Dca012589	6.146739	8.448561	9.708661	4.891635	3.63851	3.832142	4.338809	5.366244	5.472278
gene-MA16_Dca019274	1.422439	0.763748	0.724966	1.058999	1.724734	1.13301	3.199625	4.33897	2.567777
gene-MA16_Dca021067	0.676584	1.120292	0.21748	2.499221	2.894427	1.356303	2.40807	2.459791	3.396497
gene-MA16_Dca021396	51.145821	54.961819	57.737324	6.885603	6.428008	6.633959	8.821249	7.599355	8.201691
gene-MA16_Dca021398	5.170457	6.679111	7.309778	11.477241	12.099767	10.503963	18.745495	14.806131	15.763996
gene-MA16_Dca021399	12.015894	7.630285	10.708616	3.102843	3.658335	2.628489	2.34308	1.907243	3.735467
gene-MA16_Dca021931	5.362659	6.243409	4.058394	5.966723	8.631639	9.393509	14.371451	11.595048	12.221441
gene-MA16_Dca002010	10.183562	12.585708	11.048365	6.016959	6.332484	5.073012	17.370602	17.096443	15.975649
gene-MA16_Dca002014	17.876841	22.198605	21.069746	2.81749	1.786525	2.904439	4.643345	4.157517	3.844856
Dendrobium_catenatum_newGene_1863	7.062341	7.199961	7.733477	10.00007	11.416843	10.022616	5.375591	4.424488	4.837381
gene-MA16_Dca003121	19.650799	15.317845	19.854052	59.088211	56.425838	58.875481	89.888153	95.178574	89.316284
gene-MA16_Dca002293	0.850444	0.32759	0.129798	1.14786	0.708671	1.076286	4.414571	4.067217	5.500182
gene-MA16_Dca008939	0.348774	0.584096	0.429628	2.126063	3.094063	2.325522	1.524232	0.987165	2.070717
gene-MA16_Dca013344	2.479622	2.73217	1.558628	1.68539	1.72893	1.711362	0.518201	1.355281	0.786514
gene-MA16_Dca025721	34.026985	33.804504	31.076284	49.010967	42.736839	41.291992	80.856438	71.618607	75.730301

gene-MA16_Dca016113	2.642287	2.371832	2.280964	1.737677	2.154685	1.446947	0.988578	1.040187	0.915284
gene-MA16_Dca028830	5.059188	5.810926	5.543142	1.486292	2.278932	2.47497	0.905648	1.996997	1.281556
gene-MA16_Dca003623	58.409744	59.165051	52.836163	31.908451	34.413174	37.14056	24.280426	29.303635	27.533817
gene-MA16_Dca000336	80.655502	76.291962	74.671043	140.981674	183.309433	181.316696	111.298523	104.254761	95.48336
gene-MA16_Dca016705	17.677601	15.09201	17.369297	7.703902	7.664173	7.405929	15.954723	14.331013	15.655791
gene-MA16_Dca012633	41.754601	37.313236	39.22644	11.850611	10.061936	11.931542	15.422178	14.327641	17.232351
gene-MA16_Dca012756	1.839656	2.70111	2.244865	0.103441	0	0	0.020793	0.026551	0.093513
gene-MA16_Dca012759	0.088475	0.019507	0.079577	0.037274	0	0.091831	0.862245	0.847523	0.693048
gene-MA16_Dca018938	35.846344	26.027943	30.20573	3.095328	4.563255	3.846175	4.682485	5.679995	4.865986
gene-MA16_Dca028602	3.977803	4.005533	4.148496	2.219503	2.578967	1.914522	1.254119	1.621135	1.757077
gene-MA16_Dca021215	17.88698	13.97079	12.622798	0.115169	0.035701	0.828554	0.470573	0.705213	0
gene-MA16_Dca021216	24.95418	29.538343	25.773495	3.589388	3.729708	3.020276	1.660866	2.324072	2.217211
gene-MA16_Dca006081	43.416859	40.753674	44.329617	5.98975	4.274849	3.814785	6.143955	6.41612	6.077266
gene-MA16_Dca008269	0.29461	0.679148	0.827099	1.125847	0.453665	0.795161	0.25188	0.111361	0.151951
gene-MA16_Dca006555	0.539626	0.375025	0.545785	0.837978	0.647031	0.636137	1.441465	2.185937	2.111116
gene-MA16_Dca006554	31.715059	37.753269	32.779888	17.319237	17.562761	18.521145	10.602677	7.44286	8.776315
gene-MA16_Dca001024	22.579702	21.929909	22.138327	11.345784	11.551394	11.083961	9.686652	8.468639	10.579689
gene-MA16_Dca013523	175.69046	170.08667	176.62352	75.987938	70.97879	72.494545	54.684643	49.367443	48.906384
gene-MA16_Dca000646	27.847723	25.412018	28.382032	16.681326	14.790474	14.616577	10.224199	10.332355	10.237159
gene-MA16_Dca000641	6.9624	7.486274	7.162707	2.790685	2.434927	2.585579	3.489434	2.382732	3.066468
gene-MA16_Dca013596	18.381496	18.71694	15.369707	10.484704	9.823689	11.499192	6.242231	7.763161	6.510535
gene-MA16_Dca016541	0.310108	0.325045	0.123845	0.600711	0.485246	0.480167	1.223674	1.21557	0.862922
gene-MA16_Dca028197	197.579391	165.081985	205.9095	121.690422	83.545944	111.729431	66.207619	78.082909	68.918678
gene-MA16_Dca012736	36.500813	34.428204	37.622704	66.113724	68.885986	67.477135	82.266518	84.306351	87.927185
gene-MA16_Dca012732	62.057423	66.81385	68.651489	120.026398	118.291977	113.723595	264.080872	259.06134	258.374786
gene-MA16_Dca026330	0.699851	0.379615	0.525629	1.042577	1.239478	1.573759	1.172995	0.950989	0.821175
gene-MA16_Dca023679	7.617344	7.377472	6.856803	1.806651	2.252504	1.724773	3.898588	3.707273	2.774508
gene-MA16_Dca009499	1.362021	0.988337	0.766257	1.66664	1.443282	1.361457	2.728446	2.516546	3.775578

gene-MA16_Dca022100	283.12616	287.993805	281.352997	714.360168	641.929626	653.968445	314.005493	318.800507	333.165405
gene-MA16_Dca022104	66.556526	69.989601	68.079758	162.616516	155.55545	154.524338	20.59086	18.519157	21.801773
gene-MA16_Dca011819	2.074898	1.617088	2.389464	0.986478	1.564189	1.106832	1.239017	0.624291	0.454015
gene-MA16_Dca025684	14.372757	15.320045	14.251567	2.876367	1.923765	3.934699	7.259425	6.864341	5.462162
gene-MA16_Dca025688	18.078028	17.362698	16.799694	0.26536	0.062736	0.165847	0	0.272656	0.108882
gene-MA16_Dca011815	0.453906	0.724898	0.614001	4.454219	3.406946	4.888716	1.588356	1.940924	1.506055
Dendrobium_catenatum_newGene_2698	0.263395	0.402568	0.12758	0.479147	0.83443	0.492345	1.29541	0.562878	0.795825
gene-MA16_Dca013060	1.051619	0.879349	0.450692	1.257883	2.337154	2.080324	1.428586	1.344835	1.724364
gene-MA16_Dca019600	2.751595	2.459354	2.058489	0.688207	0.877903	1.279745	1.014316	0.648074	1.740285
gene-MA16_Dca019605	1.799099	2.170932	1.843671	0.393564	0.180617	0.863064	0.986357	0.576276	0.175455
gene-MA16_Dca021769	11.450897	9.774193	8.889373	11.638506	12.850528	16.885586	30.567362	27.412867	30.676006
gene-MA16_Dca020415	9.440959	8.142774	8.399698	5.33477	4.770756	5.438354	4.033429	4.868227	3.987011
gene-MA16_Dca023994	1.2435	1.616144	2.609643	0.962697	0.609884	0.888004	0.528936	0.297268	1.324206
gene-MA16_Dca023991	0.939617	1.111005	1.04845	1.790785	1.949983	1.907262	2.834352	3.046575	2.128285
gene-MA16_Dca002301	6.034251	4.866084	4.476252	3.115129	3.887646	2.690087	2.449132	2.805305	2.573811
gene-MA16_Dca017595	17.148674	19.811836	17.961435	2.013629	1.472257	1.936283	1.504502	0.896511	0.859842
gene-MA16_Dca005837	16.871752	16.271225	18.003914	34.216129	30.663124	33.504997	44.076408	51.003704	43.460659
gene-MA16_Dca005836	3.633027	2.853194	3.232489	1.084615	1.179564	0.891328	0.974461	1.157643	1.078037
gene-MA16_Dca021400	13.672129	13.544176	14.217912	4.093509	7.287108	3.619697	3.098798	5.358202	4.920438
gene-MA16_Dca021401	432.554657	432.455383	426.967194	760.255493	701.682373	761.756226	1245.05481	1201.27942	1202.71021
gene-MA16_Dca015017	65.959953	69.012924	60.452435	1.071413	2.122379	1.601251	4.58136	4.643504	3.014157
gene-MA16_Dca018566	11.92712	11.26083	10.777637	3.980756	5.322628	6.840888	4.578956	4.696503	4.899789
gene-MA16_Dca003005	13.576363	12.886905	17.2264	14.844312	18.521202	17.736723	36.17728	32.373184	29.664049
gene-MA16_Dca008437	2.324162	2.427119	2.219723	0.931021	1.284894	1.235074	0.915159	1.327374	1.511197
gene-MA16_Dca003414	2.419126	2.391014	2.876236	0.788912	0.93895	0.751646	2.104469	2.76085	2.252349
gene-MA16_Dca012081	11.280355	8.793203	10.66974	3.174076	4.31708	3.894754	4.016693	2.62464	4.391533
Dendrobium_catenatum_newGene_4814	15.279175	14.365472	15.563971	3.693071	2.828429	3.74319	4.629928	4.370427	5.027854
gene-MA16_Dca000853	73.707954	74.610703	69.360146	29.075369	32.582008	33.16433	31.423225	31.978825	33.339218

gene-MA16_Dca000859	0.080844	0.126562	0.075965	0.141219	0.313549	0.23164	0.942877	0.595014	0.84333
gene-MA16_Dca028782	107.228317	121.459404	111.700302	59.154537	57.282463	56.654579	90.661659	94.509834	94.323792
gene-MA16_Dca028432	49.069916	56.1133	54.157974	0.518965	0.573254	0.350114	1.603139	1.867395	1.556111
gene-MA16_Dca008881	1.100973	1.100291	1.152689	2.052029	2.074566	2.255946	1.069229	0.963072	0.882626
gene-MA16_Dca017752	0.703196	0.568219	1.280749	3.053283	3.109805	2.114176	4.311031	4.311492	4.403704
gene-MA16_Dca022379	0.787988	1.103249	1.836079	3.429167	3.481614	3.15171	1.863039	1.981922	3.664247
gene-MA16_Dca024929	25.74773	21.501513	21.201223	25.480522	22.8235	24.795979	8.369024	7.890027	7.870515
gene-MA16_Dca024926	20.997856	22.068296	24.999084	3.520517	3.277011	3.404941	2.632458	2.882229	1.760611
gene-MA16_Dca011973	8.614626	9.326377	11.113053	3.007446	2.615183	2.787263	5.475179	5.981509	6.204602
gene-MA16_Dca014220	119.246437	117.255653	125.240631	73.138329	80.90947	79.530205	65.084854	62.438889	54.42173
gene-MA16_Dca018760	2.939046	2.624605	2.41085	0	0.778843	0.400279	0.570568	0.259026	0.787744
gene-MA16_Dca020595	137.62735	140.814728	135.59259	49.820946	48.942631	50.22361	48.118523	47.208111	50.49485
gene-MA16_Dca027649	22.601	23.553459	21.65023	2.425705	2.2459	2.626081	2.336664	2.610746	2.205005
Dendrobium_catenatum_newGene_6102	1.505964	1.179591	1.2339	2.719763	3.723251	3.889398	3.807192	3.133167	3.105787
gene-MA16_Dca026998	49.24094	50.076096	51.212166	56.577614	51.745426	59.05254	24.911781	19.160006	18.591291
gene-MA16_Dca026996	0.789516	1.576593	2.10605	7.079668	3.398671	6.272202	10.57585	10.681898	10.222921
gene-MA16_Dca026995	7.882354	9.279314	10.260575	16.151775	16.401892	16.560646	101.491196	93.235199	98.10154
gene-MA16_Dca001279	2.486213	2.619007	2.596589	3.623865	3.844445	3.405607	6.60712	7.672298	6.817839
Dendrobium_catenatum_newGene_9944	0.117243	0.206139	0.192021	0.230734	0.82525	0.41135	0.774835	0.70125	0.806075
gene-MA16_Dca017252	1.493397	2.453103	1.724421	3.252628	4.632741	3.633858	6.804303	7.144999	5.143853
gene-MA16_Dca017256	46.522949	46.418896	41.686359	9.486532	9.087682	9.518305	9.007455	8.755187	8.363094
gene-MA16_Dca009043	0.746169	0.613781	0.414974	0.961373	0.708324	1.28245	1.992793	4.713508	1.744044
gene-MA16_Dca006242	60.757122	63.701595	60.166283	31.826218	26.776043	29.206955	49.511803	50.157383	41.616894
gene-MA16_Dca018845	4.914037	5.42317	4.338019	2.110952	2.699349	1.659283	3.134302	2.519658	2.434696
gene-MA16_Dca001734	2.386782	3.243402	4.428099	4.097242	4.75992	5.07693	9.817783	5.195798	8.883451
gene-MA16_Dca008603	4.162632	3.611772	4.369736	10.199544	9.660613	12.312757	15.149525	16.479259	16.575848
gene-MA16_Dca008608	24.022438	24.340893	28.298782	7.043481	6.348764	7.986384	5.340582	3.827541	4.351541
gene-MA16_Dca012323	19.250475	21.988054	22.048935	3.562486	2.39437	2.796079	4.276102	3.305166	3.515043

gene-MA16_Dca012324	3.069645	2.11702	2.384206	0	0	0.127731	0.185878	0.740142	0.118168
gene-MA16_Dca011789	1.768929	1.000816	0.730178	4.850949	3.531593	3.646431	5.880185	9.956285	6.608152
Dendrobium_catenatum_newGene_10270	44.597648	42.547748	43.093572	5.950052	4.572826	4.487972	5.231009	5.683674	5.217164
gene-MA16_Dca015693	186.717453	187.354675	198.429657	103.805145	93.861595	99.306015	101.999374	100.707047	98.030846
gene-MA16_Dca011430	0.233355	0.178837	0.067118	0.509572	0.852869	0.704767	0.782894	0.319704	1.199314
gene-MA16_Dca011437	3.232567	2.831972	3.607587	7.845395	7.170053	7.675014	6.483412	6.581145	6.210235
gene-MA16_Dca003601	30.522472	30.417076	27.459904	18.584295	15.475249	15.353321	15.701795	14.747753	14.378609
gene-MA16_Dca013492	18.073643	19.704657	18.116001	5.220911	4.769777	4.329494	5.241272	4.265942	6.663382
gene-MA16_Dca013491	15.606444	17.865971	16.708021	8.874722	7.680909	8.136248	12.479753	10.50851	7.73125
gene-MA16_Dca013496	12.168482	13.775951	11.933109	1.661323	1.29956	1.391522	4.260672	3.597348	2.976031
gene-MA16_Dca002555	0.407767	0.410993	0.471239	0.241451	0.218437	0.215714	0.227668	0.198357	0.185904
gene-MA16_Dca013149	13.641515	13.213708	14.281076	0.920371	0.322863	1.355487	0.844249	0.428914	0.291763
gene-MA16_Dca013146	40.830502	43.817802	41.217785	4.767673	4.99877	5.868849	6.859934	5.53651	7.110829
gene-MA16_Dca013140	1.099972	0.834249	1.430427	1.975363	3.04462	3.467623	3.353212	3.678893	2.447962
gene-MA16_Dca013141	52.859818	54.19548	51.705982	35.161644	32.897484	35.429359	27.341627	25.182295	28.701075
gene-MA16_Dca023746	1.361628	1.812592	1.594885	0.547582	0.46924	0.387411	0.639661	0.711698	0.748619
gene-MA16_Dca023743	9.836678	11.249011	10.352073	8.530229	7.115743	5.638126	19.435547	18.513765	17.263187
gene-MA16_Dca009568	0.589227	0.591905	0.538633	0.950182	1.601273	1.46103	1.586873	1.844836	1.23828
gene-MA16_Dca017498	28.879576	26.930817	30.809801	5.486957	4.499387	1.491608	4.512545	4.950642	3.533805
gene-MA16_Dca017499	8.540038	8.01945	7.892347	2.056269	3.582722	1.683431	2.202748	2.961726	1.509879
gene-MA16_Dca019330	2.619415	2.841008	1.775285	1.447863	1.761234	1.373467	4.10163	3.004365	3.932871
gene-MA16_Dca019333	74.385719	78.012558	74.270569	124.746178	122.591705	117.291618	172.343658	166.450729	170.160767
gene-MA16_Dca024719	19.545588	18.645496	19.762434	7.004239	8.242469	7.622202	8.875725	10.665547	9.928963
gene-MA16_Dca024717	4.988059	4.783084	5.158648	2.084817	2.150122	1.915653	2.60399	1.596516	2.117274
gene-MA16_Dca019594	1.612078	1.327204	1.080511	0.83556	0.206074	0.607206	0.931297	0.280346	0.914043
gene-MA16_Dca021299	7.541304	7.741376	7.572738	1.476073	1.495101	0.895916	1.370528	0.71139	0.793953
gene-MA16_Dca023963	4.496747	5.097569	4.121806	0.365099	0.706541	0.545914	1.466237	1.571771	1.696145
gene-MA16_Dca000969	0.716609	0.207124	0.166765	0.305039	0.244416	0.112621	1.62894	1.209237	1.275638

gene-MA16_Dca027439	0.872035	1.330942	1.387055	0.459429	0.448709	0.312407	0.261396	0.357456	0.361064
gene-MA16_Dca020818	77.721642	70.340485	76.081078	13.66848	13.660193	13.930296	20.200676	16.613861	21.348761
gene-MA16_Dca018334	12.690687	14.340178	11.761597	3.557338	4.448421	4.561255	2.815939	2.04021	3.493901
gene-MA16_Dca006003	13.715981	15.570245	15.138266	6.803293	7.703155	8.729364	6.33685	6.296913	4.746224
gene-MA16_Dca005722	21.466736	19.959085	17.349169	11.295634	12.607924	10.848762	7.712151	8.264527	9.865269
gene-MA16_Dca005857	3.264544	3.395716	3.685109	1.529873	1.046287	1.084783	0.498784	0.572556	0.230466
gene-MA16_Dca003950	17.456348	17.031326	18.121372	6.851181	5.813931	6.35255	4.626729	5.426896	4.484854
gene-MA16_Dca002021	3.287295	2.45962	2.394142	2.604592	2.91725	2.217089	5.861323	8.118483	7.868758
gene-MA16_Dca016119	0.456049	0.563949	0.360201	0.995846	0.955604	0.737124	2.157461	1.409871	1.329158
gene-MA16_Dca024558	1.773387	1.170722	1.653664	8.377999	6.452819	9.601232	0.2478	1.200974	0.334044
Dendrobium_catenatum_newGene_16914	47.794075	46.219524	53.716198	7.771771	7.771759	10.203952	8.773293	6.938619	6.628057
gene-MA16_Dca010087	37.179752	35.909931	41.844753	18.874166	18.280249	16.029884	17.742073	18.081188	19.553238
Dendrobium_catenatum_newGene_14404	2.059943	2.758655	2.369392	0	0	0	0	0.263924	0
gene-MA16_Dca010626	28.374426	27.8482	27.054928	21.887135	20.371302	21.125988	57.014198	54.356319	55.701099
gene-MA16_Dca023730	12.540042	13.406954	13.325459	18.87945	16.315327	21.194736	27.383394	31.678907	28.112989
gene-MA16_Dca023731	3.7755	2.4934	3.008794	8.297211	10.507377	11.875751	20.504559	20.299417	18.565844
gene-MA16_Dca000563	4.665293	5.265604	5.285934	2.688835	2.005718	2.25116	2.300952	2.672851	2.512389
gene-MA16_Dca000560	28.01317	25.231401	26.989922	13.827211	13.040193	13.121932	12.754531	12.976315	12.764253
gene-MA16_Dca016661	103.249687	101.944946	94.001335	25.534615	26.137634	24.177195	25.411259	23.680735	24.107693
gene-MA16_Dca017045	5.738208	5.556069	5.999593	1.407997	1.521799	1.720668	1.660225	2.234595	1.399117
gene-MA16_Dca008487	1.846194	1.864045	1.808912	5.596331	4.137138	5.676678	5.204988	4.441821	4.814641
gene-MA16_Dca022466	0.808054	1.164912	1.150384	2.754213	3.198802	2.620367	2.109833	1.891693	1.894178
gene-MA16_Dca006602	74.005318	66.124001	71.565506	17.414942	44.907406	16.628395	21.77153	30.360748	29.142292
gene-MA16_Dca021481	13.206264	12.698703	11.069509	10.701864	9.396873	9.403752	40.101482	34.195015	33.091061
gene-MA16_Dca021482	3.180641	1.943713	2.340939	2.220041	1.512732	1.826911	0.79008	0.996502	0.332575
gene-MA16_Dca020756	71.845314	66.797935	70.897705	31.121508	22.887085	30.446432	36.54607	45.699585	28.693892
gene-MA16_Dca020755	24.476582	23.489494	22.813761	4.717737	3.66748	4.493362	4.948535	4.766914	4.177467
gene-MA16_Dca020753	5.561392	5.407404	4.905918	0.898813	0.950589	1.6499	1.212925	0.899164	1.18592

gene-MA16_Dca020752	17.436306	14.377192	18.276283	8.214264	7.686444	7.806939	7.512171	6.170334	6.671571
gene-MA16_Dca020750	1.252558	1.396801	1.481123	2.631606	3.505702	3.269243	3.595055	3.438941	4.224724
gene-MA16_Dca007255	32.333546	31.653658	34.150669	14.468893	16.291407	14.12717	16.278629	13.944231	16.061068
gene-MA16_Dca023006	46.549671	46.793583	42.646557	8.86553	9.292439	7.554417	10.047732	12.333953	11.371001
gene-MA16_Dca001961	618.007263	572.066711	602.510559	199.342407	195.30751	207.475983	174.876266	177.339828	156.069565
gene-MA16_Dca001965	8.755512	7.657007	8.22574	15.509728	13.176636	14.205403	22.625032	20.129211	20.904192
gene-MA16_Dca006614	2.079762	2.600602	2.096035	4.327442	5.191478	5.507735	2.973294	3.463912	2.572125
gene-MA16_Dca001340	107.806297	114.281181	109.584648	25.183187	26.345078	25.411369	13.264496	12.65976	15.296001
Dendrobium_catenatum_newGene_4898	9.715736	9.262827	10.093651	1.626672	3.076115	3.145074	3.059035	2.275422	0.974092
gene-MA16_Dca015291	0.349534	0.350926	0.476108	0.815468	1.079009	1.016798	2.221879	1.918662	2.386021
gene-MA16_Dca003233	3.407817	6.6057	2.293375	9.358157	10.029029	5.933899	16.42874	12.786035	11.138921
gene-MA16_Dca028706	12.586219	14.06409	15.557288	4.631042	2.532282	2.350097	2.467517	1.429094	2.248204
Dendrobium_catenatum_newGene_6239	6.575426	4.390238	7.209244	11.241385	7.812823	8.894777	15.366231	13.15499	14.355589
gene-MA16_Dca008807	0.571026	1.342833	0.575953	3.554023	2.49229	2.467766	2.163289	2.497217	3.772381
gene-MA16_Dca013232	3.41106	3.937462	3.756619	2.813403	3.542248	2.912428	1.21378	1.374188	1.536457
gene-MA16_Dca013236	5.243225	5.072171	3.506537	3.308479	3.492732	2.532314	2.233454	2.017395	2.347362
gene-MA16_Dca013237	89.956665	87.992233	90.97757	37.241924	33.675255	34.966919	33.007404	28.876001	27.58514
gene-MA16_Dca024070	1.797044	2.199689	2.178212	1.179088	0.831231	1.132238	0.831782	0	0.652402
gene-MA16_Dca024071	1.97539	3.062554	3.040575	3.951655	5.23427	4.400774	8.053465	6.535772	8.448073
Dendrobium_catenatum_newGene_11187	1.218392	1.065304	1.335041	0.099526	0.482381	0.29602	0.397423	0.264644	0.327936
gene-MA16_Dca026002	25.708506	26.247257	27.698397	12.468369	11.226394	8.584459	18.474121	15.053986	20.125275
gene-MA16_Dca019759	55.686234	60.81321	55.136463	70.722771	69.470543	70.50119	119.002151	124.392807	121.949364
Dendrobium_catenatum_newGene_1604	0.761067	1.290182	1.480552	1.00893	1.498403	2.131072	3.079366	3.426141	3.704138
Dendrobium_catenatum_newGene_1603	2.761573	2.285488	3.021985	3.105285	4.783392	4.613935	9.823048	9.163038	10.528431
gene-MA16_Dca004932	1.472854	1.224821	1.080383	3.456121	4.419974	3.841321	8.349778	4.469302	8.070464
Dendrobium_catenatum_newGene_7524	6.034744	5.4581	5.926003	2.36893	1.838	1.810777	2.272643	2.620531	2.53292
gene-MA16_Dca006425	1.293847	1.486873	0.969663	0.553786	0.813779	0.43774	0.254377	0.869802	0.592723
gene-MA16_Dca006421	103.672089	112.553658	112.57077	6.101269	5.028955	5.276577	4.014305	4.201046	3.27264

gene-MA16_Dca006428	577.211853	599.836914	562.810303	71.796501	71.265594	69.417587	97.50058	91.710762	96.161514
Dendrobium_catenatum_newGene_15098	34.739748	38.386284	33.739022	14.029102	9.582793	10.014068	12.874722	11.57009	9.473654
gene-MA16_Dca004483	26.065937	29.402992	31.639256	6.375833	7.707114	7.421489	17.330568	17.64979	14.670007
gene-MA16_Dca008681	103.701775	100.462784	95.821869	3.955209	2.53387	2.814332	1.048497	1.694376	0.725295
gene-MA16_Dca008685	102.014984	100.611473	111.255341	122.481812	127.857903	109.667992	281.818604	231.302582	255.592636
gene-MA16_Dca022606	11.45614	13.720873	12.132385	16.275084	13.803703	15.260028	29.810017	29.737141	28.532879
gene-MA16_Dca003680	44.000328	43.354565	44.242332	15.163366	11.720742	13.09304	11.89403	10.524882	11.92444
gene-MA16_Dca011709	12.92225	12.926784	12.878722	17.159294	15.335714	15.764896	45.278061	44.595837	45.79932
gene-MA16_Dca011706	27.927204	25.334551	31.169027	48.619179	48.172676	53.490761	71.698677	82.875603	67.745979
gene-MA16_Dca013411	5.818428	6.865199	6.899131	11.733448	10.016381	11.57406	13.027611	13.711686	14.66146
gene-MA16_Dca009658	0.833275	1.216865	0.668665	2.240016	1.495278	1.915861	1.954668	2.781827	2.735026
gene-MA16_Dca016165	1.472677	0.99682	1.727632	0.514436	0.450491	0.791067	3.002348	1.936655	2.396836
Dendrobium_catenatum_newGene_16529	0.827355	0.462331	0.763412	1.199779	1.376028	2.119787	0.738748	1.530737	1.059333
gene-MA16_Dca025915	64.071121	56.122589	57.745361	8.403287	7.064623	6.83695	7.678751	6.89318	7.972718
gene-MA16_Dca017418	16.391693	17.753166	14.883648	6.866187	5.885159	4.126163	4.11918	4.5711	4.222287
gene-MA16_Dca022035	6.207791	4.409066	3.905539	1.75035	1.726581	1.382846	0.675742	0.595376	0.526083
gene-MA16_Dca028710	135.615417	138.558151	136.216599	41.170158	42.474792	39.264805	43.915878	30.916025	41.540733
gene-MA16_Dca019510	4.002693	3.317993	3.451093	1.011217	1.04261	0.886847	1.411771	0.412267	0.478749
gene-MA16_Dca025262	12.727157	11.995938	12.692677	25.150866	25.33304	24.072905	28.720512	30.529753	31.535606
Dendrobium_catenatum_newGene_10039	2.006725	2.455326	1.670177	2.005154	1.225482	1.262299	1.126143	0.515101	0.556333
gene-MA16_Dca010132	128.164734	124.552841	123.466286	69.190361	64.655678	63.996578	70.280769	63.422131	66.842201
gene-MA16_Dca001535	36.926453	39.887589	42.680283	12.295716	10.711347	12.625419	11.265434	9.306492	9.22168
gene-MA16_Dca018361	13.437832	11.268531	12.644709	0.404082	0.352885	0.70303	0.11591	0	0.21447
gene-MA16_Dca004017	26.610056	23.706209	24.304749	35.431076	39.141087	37.059669	70.367233	64.778603	68.403671
gene-MA16_Dca004010	10.472411	9.390699	11.519279	5.360954	5.170995	5.500863	5.616339	5.055366	5.148834
gene-MA16_Dca007091	0.348547	0.096471	0.169838	0.664409	0.378013	0.452143	0.763286	0.571987	0.548725
gene-MA16_Dca007099	1.753222	1.776203	2.106109	0.712516	1.372739	0.65736	0.628894	1.107428	0.732663
gene-MA16_Dca007638	98.304047	108.523621	107.515465	23.764084	26.081173	25.203751	26.79632	23.44245	24.467024

gene-MA16_Dca006325	0.654769	0.640983	0.519765	0.172849	0.133574	0.277885	0.652324	0.741366	0.620116
gene-MA16_Dca006323	2.19852	2.341585	2.721756	1.9844	1.437174	1.917544	4.972778	3.815412	4.05536
gene-MA16_Dca006320	481.583557	486.277954	461.582916	209.860657	199.490128	208.599167	198.840591	227.219864	223.359634
gene-MA16_Dca006321	3.027029	1.882609	2.038492	3.698817	3.540334	3.790357	5.178793	5.786359	5.813104
Dendrobium_catenatum_newGene_124	0.564735	0.494795	0.557874	1.016113	1.375441	1.320752	0.878663	0.960371	1.118275
gene-MA16_Dca008182	4.074468	4.798277	4.664312	3.745604	3.551168	2.92169	1.588449	1.995573	1.953501
gene-MA16_Dca008189	6.30666	5.625268	4.90959	9.500414	11.477681	10.903803	15.751886	12.106339	12.701021
gene-MA16_Dca000078	8.07154	7.161716	4.734348	1.683685	1.280362	2.148335	3.110575	3.845487	3.813244
gene-MA16_Dca008721	4.451554	4.222323	4.562741	8.208678	9.580788	8.837539	22.689825	21.784616	22.676842
gene-MA16_Dca008728	52.027145	49.141636	54.387455	27.03274	26.482971	27.127808	28.162262	32.710617	30.967936
gene-MA16_Dca012246	23.807768	18.133711	20.538687	58.229252	65.951538	65.432579	124.632286	133.349854	111.737503
Dendrobium_catenatum_newGene_10337	1.958706	1.735944	1.586898	0.346894	0.448996	0.299104	0.899957	0.445783	0.454994
gene-MA16_Dca013592	16.823477	17.213533	17.141624	7.007859	7.168813	8.459026	6.611669	6.351923	6.340403
gene-MA16_Dca002694	1.647041	2.010123	1.433733	2.888273	2.731073	3.900915	4.214738	3.831918	4.319565
gene-MA16_Dca009442	4.011442	3.171645	3.108701	2.742066	2.730525	2.201168	5.896289	6.303842	6.579893
gene-MA16_Dca002344	46.043842	45.214237	46.189919	30.639954	28.164904	30.164448	20.233545	24.017694	21.34437
gene-MA16_Dca002346	3.36029	4.368906	4.043497	10.813385	9.965106	9.498718	17.15139	17.248741	18.483404
Dendrobium_catenatum_newGene_12227	17.246023	14.980519	14.731099	4.747147	4.711686	4.09401	4.704218	5.5149	4.863878
gene-MA16_Dca018567	28.84709	28.88505	25.674513	9.091374	10.586275	13.197415	9.383427	10.531015	9.519834
gene-MA16_Dca017628	2.187065	3.002535	2.325094	3.122013	1.971576	1.675979	0.858951	1.03802	0.514762
gene-MA16_Dca017627	4.160466	2.925686	3.276531	3.764476	3.31944	2.369967	1.706921	1.13406	1.458385
gene-MA16_Dca017621	1.199253	2.308422	1.414898	0.069087	0	0.18889	0.3074	0.208053	0
gene-MA16_Dca026450	6.327521	6.895947	6.657297	1.782084	0.929558	1.483164	1.084357	1.30036	1.345298
gene-MA16_Dca021157	1.431224	1.210812	2.31624	4.767343	4.639595	5.397563	5.393615	6.816071	3.821206
Dendrobium_catenatum_newGene_11199	0.403505	0.590328	0.678681	1.545561	0.907433	1.531183	2.49241	0.963688	2.258418
gene-MA16_Dca015736	67.856247	64.809517	67.547165	37.325981	39.409103	40.787144	30.425295	30.382387	26.063845
gene-MA16_Dca001471	16.73506	16.214964	13.187758	10.92348	12.422376	11.489163	6.400443	6.765849	5.566232
gene-MA16_Dca004228	83.118088	80.786179	78.934822	35.402615	30.64077	33.35199	53.768749	50.817226	52.46608

gene-MA16_Dca004224	154.270447	153.012421	156.609039	81.126419	79.591896	78.183624	94.573288	92.280067	77.385727
gene-MA16_Dca004226	112.782845	111.200264	113.132759	42.275341	43.634945	45.162083	32.629688	33.152531	32.328602
Dendrobium_catenatum_newGene_9761	1.255007	0.899171	1.000242	0.463411	0.459658	0.232057	0.835436	0.65836	0.553854
gene-MA16_Dca002101	37.825451	39.717426	38.795078	24.616282	21.167946	22.26795	19.554285	20.53533	17.821476
gene-MA16_Dca018747	29.132641	31.611317	32.304749	114.401604	104.873665	114.239433	65.823524	74.127701	71.308174
gene-MA16_Dca009132	15.310203	16.533525	14.475851	6.383309	7.110242	6.490065	13.605052	12.517573	12.443871
gene-MA16_Dca009134	0.287161	0	0	1.15367	0.839297	0.351199	3.188499	3.110783	2.851948
gene-MA16_Dca012855	44.275787	40.131027	42.74062	9.545417	9.932446	9.05361	12.667234	14.50215	13.825112
gene-MA16_Dca009138	99.20977	85.317154	95.493401	46.381939	51.274483	47.334393	38.670589	36.81068	39.221893
gene-MA16_Dca012853	33.467949	33.099907	38.470882	17.858593	16.598501	18.582066	27.256453	21.559704	26.915768
gene-MA16_Dca002815	17.313261	17.602613	17.455133	29.24962	27.229687	28.612415	53.848007	50.314915	50.919224
gene-MA16_Dca002818	76.7015	78.556496	73.40818	24.24143	23.320814	20.797724	16.993723	18.280317	15.972157
gene-MA16_Dca019941	58.722038	61.581333	56.325115	28.682852	28.703636	30.75054	29.929474	31.589907	31.976065
gene-MA16_Dca025327	124.802292	124.489212	128.551727	124.300377	156.123123	144.649506	297.58429	278.829224	275.362549
gene-MA16_Dca010247	1.497253	1.465963	2.432507	4.088834	3.333304	3.42451	4.9992	5.302083	4.406107
gene-MA16_Dca027060	194.128647	188.292694	187.839142	82.481903	76.266144	75.067352	72.374405	65.400482	67.155785
gene-MA16_Dca027061	201.116318	203.548355	193.90799	88.035851	83.433762	91.38047	56.905655	61.478348	67.472107
gene-MA16_Dca017122	0.911641	1.00932	0.591342	0.145821	0.26652	0.173065	0.056321	0.121975	0.156143
gene-MA16_Dca022509	8.464237	11.104244	8.606768	15.861105	10.838417	12.41842	0.80803	2.046244	2.28834
gene-MA16_Dca014474	0.204946	0.409094	0.473356	0.690516	0.685322	0.504026	2.850817	2.888959	4.332316
gene-MA16_Dca022502	2.799862	2.793388	1.839973	0.825178	0.611433	0.825951	0.688583	1.224659	0.645294
gene-MA16_Dca018151	0.987773	0.680634	0.602853	0	0	0	0	0.18614	0.025609
gene-MA16_Dca017787	1.314979	0.915531	0.680944	1.44326	2.225204	1.943911	2.823486	2.901742	3.719125
gene-MA16_Dca018159	0.435528	0.549062	0.594702	0.975826	0.714616	0.683589	2.134835	1.202508	1.050576
gene-MA16_Dca014781	25.27211	25.211569	22.792334	8.881583	7.442062	6.336221	9.633058	8.977515	9.242822
gene-MA16_Dca021058	3.713116	3.632125	4.538158	5.863342	4.843236	5.827784	8.379115	10.204506	10.49543
gene-MA16_Dca015430	1.270863	1.271878	1.374499	2.176976	2.222199	2.641894	3.633688	2.629844	2.712716
gene-MA16_Dca015431	4.253742	3.42533	3.696445	7.274945	6.57856	7.83422	9.551747	11.701255	12.79234

gene-MA16_Dca009353	23.042522	18.809269	18.972881	7.249478	8.940759	5.260386	5.076502	8.988096	6.246507
gene-MA16_Dca020016	6.999515	4.711761	6.937274	2.984555	3.172377	2.58516	4.823391	4.996512	3.53949
gene-MA16_Dca004405	165.265305	164.331238	162.338196	54.807178	51.138195	51.656391	58.574898	55.100361	57.07058
gene-MA16_Dca004404	78.151283	80.75164	77.448082	22.972815	24.563974	28.323402	11.714057	8.822197	11.093137
gene-MA16_Dca004406	84.359924	81.89502	81.229691	40.777958	40.378384	34.211384	18.419653	14.432117	15.559163
gene-MA16_Dca004401	184.723862	187.652374	201.373306	17.225477	23.106756	17.744856	12.500439	9.785314	9.614083
gene-MA16_Dca008573	38.281391	29.882673	42.66008	9.414371	21.956459	10.468069	9.416116	14.884788	10.839203
gene-MA16_Dca008575	69.8265	65.756851	63.953503	35.193832	34.144611	31.852167	38.125679	36.503792	36.454529
gene-MA16_Dca001151	0.770436	0.991275	0.928991	0.255839	0.730243	0.340305	0.097767	0.198394	0
gene-MA16_Dca004657	3.8717	5.89029	5.997135	2.679997	2.627434	2.788201	1.91973	2.686881	1.811432
gene-MA16_Dca012900	160.768402	167.153275	174.19313	126.90786	115.263031	118.291115	84.541153	80.696526	77.234489
gene-MA16_Dca028170	5.582323	4.331089	5.744275	11.656896	9.698138	11.691899	12.821494	12.733191	11.199642
gene-MA16_Dca028175	275.349152	275.165649	266.645966	147.889221	141.249023	143.429153	118.764877	104.759216	115.995392
gene-MA16_Dca010943	10.380017	11.71589	11.919525	25.960361	23.307793	28.886885	13.864779	11.883847	13.716912
Dendrobium_catenatum_newGene_7801	10.553647	11.080901	11.583448	6.009098	6.334708	5.261421	5.749195	5.237228	6.038399
gene-MA16_Dca007126	0.743417	1.62015	1.322892	2.338688	2.773489	3.536677	2.915129	2.301717	1.868435
gene-MA16_Dca008327	9.070103	10.313351	10.113477	30.759068	29.743492	29.567142	50.452293	42.736561	40.992138
gene-MA16_Dca013998	8.506045	8.334637	9.318428	16.940863	16.473673	16.543058	23.375814	20.211769	21.470785
Dendrobium_catenatum_newGene_3498	1.269086	0.711655	1.171155	0.962661	0.840837	0.977263	0.41367	0.554931	0.136146
gene-MA16_Dca015901	3.307017	2.629472	2.005875	2.6003	2.532528	2.097758	6.685507	5.365084	6.428637
gene-MA16_Dca015906	0.735966	0.937186	0.573739	3.223735	4.088661	3.9524	3.765689	3.332561	3.84653
gene-MA16_Dca027381	116.339005	134.228439	129.188721	54.391354	41.379055	52.924786	26.059254	25.835705	28.010368
gene-MA16_Dca027382	128.62735	134.670013	136.237671	67.251747	64.397667	75.458588	71.165489	62.129208	60.495152
gene-MA16_Dca019011	22.452179	28.217531	24.006489	4.959863	4.837951	4.172052	4.580463	4.217752	4.433481
gene-MA16_Dca015572	61.094498	51.621471	57.083687	19.230822	19.137215	23.595127	13.247279	12.882863	16.599144
gene-MA16_Dca015577	10.286148	8.87839	10.237609	0.160089	0.09825	0.437343	0.581496	0.078603	0.559349
gene-MA16_Dca015576	5.876374	4.961871	5.761222	0.094338	0.216536	0	0	0.216622	0
gene-MA16_Dca015574	2.73952	4.255828	2.066972	0.949077	1.663241	0.439615	0.522189	0.682978	0.437661

gene-MA16_Dca001256	0.100155	0.159022	0.02489	0.228275	0.134719	0.11588	2.197581	1.672257	1.284417
gene-MA16_Dca005251	2.374645	2.109602	3.078088	1.884629	1.518792	1.257559	0.928107	1.270621	1.100672
gene-MA16_Dca005256	0.783487	0.561749	0.506034	1.41171	1.044094	1.092179	1.164191	1.618363	1.582039
gene-MA16_Dca027544	21.752104	20.390236	20.497969	27.955894	28.973978	28.940739	55.918175	47.493389	56.005257
gene-MA16_Dca010339	48.818962	45.926914	46.235512	4.964004	5.327466	5.885215	9.671349	8.041576	10.648259
Dendrobium_catenatum_newGene_9464	4.561819	4.066871	3.166544	0.259521	0.683099	0.331153	0.992177	0.741143	0.727498
gene-MA16_Dca011068	19.944254	17.554756	20.239071	8.904773	6.937733	7.422277	6.86442	6.649683	6.177563
gene-MA16_Dca011061	0.546269	0.437612	0.472513	1.006019	0.993365	1.085226	0.695578	1.092029	1.451618
gene-MA16_Dca013514	0.868756	1.331265	0.944361	0.638322	0.270322	0.366275	0.74439	0.675801	1.197806
gene-MA16_Dca016598	0.17877	0.405372	0.014817	1.753765	1.514292	1.552117	6.151312	6.126907	5.514235
gene-MA16_Dca016597	0.047291	0	0.188536	0.12391	0.381423	0	1.325332	1.401843	1.483577
gene-MA16_Dca002614	236.0168	219.788391	221.597153	113.903473	115.940155	117.917633	101.063148	100.883972	103.902046
gene-MA16_Dca002619	15.354406	17.866011	19.120419	5.048756	8.423977	5.114792	3.621001	2.748715	2.653534
gene-MA16_Dca016047	1.215322	2.094376	3.29695	8.61212	7.282031	8.835866	8.577407	9.929128	10.197039
gene-MA16_Dca016043	0.146986	0.223187	0.239775	0.163127	0.202116	0.338192	0.993484	1.010463	1.785884
gene-MA16_Dca020990	451.114258	445.639435	428.061707	196.970749	204.035645	201.777634	148.890457	122.28363	160.763
gene-MA16_Dca020992	209.298035	172.844833	247.071777	70.148094	154.135147	74.155014	99.453308	93.205399	106.423637
Dendrobium_catenatum_newGene_2061	3.800637	3.040684	2.951973	0.762775	0.486533	0.718895	0.73153	0.701233	0.528365
gene-MA16_Dca012749	7.586142	8.933111	7.936367	17.417343	18.960543	20.534117	24.277445	25.91662	27.529968
gene-MA16_Dca012745	0.701109	0.976158	1.069728	6.259858	3.696162	3.260313	2.314783	4.169542	4.525181
gene-MA16_Dca011804	0.540566	0.878837	0.709434	1.10628	0.556117	0.838872	2.2291	2.006674	1.883341
Dendrobium_catenatum_newGene_2933	3.553375	2.911145	2.414396	4.650664	3.960058	5.185942	6.186038	5.919367	6.586832
gene-MA16_Dca014352	85.53241	82.084427	85.114265	51.481972	48.66465	51.414898	40.612572	39.564835	39.435314
gene-MA16_Dca014972	1.608681	1.503259	2.681292	0.545628	0	0.398474	1.050838	0.530404	0.305828
gene-MA16_Dca015005	0.34075	0.696115	0.383072	2.180112	1.267289	1.478207	3.558214	3.681337	4.763478
gene-MA16_Dca015057	3.495002	3.226665	1.741977	0	0	0.071927	0	0.062937	0
gene-MA16_Dca015053	0.627926	0.571124	0.515415	0.186781	0.210237	0.155182	0.099003	0.240563	0.186033
gene-MA16_Dca028958	76.826843	72.495659	68.662697	76.819656	74.879143	76.238495	182.15152	170.77594	167.535309

gene-MA16_Dca027194	3.880104	2.634894	2.774045	8.612286	8.81782	11.49614	8.034765	6.4087	4.601589
gene-MA16_Dca027195	2.679822	1.662709	1.227844	4.02202	4.369359	4.398906	4.223558	3.386614	2.468402
gene-MA16_Dca025846	11.265182	7.049494	11.361902	5.055788	4.121376	4.579413	5.197424	6.392736	7.804476
gene-MA16_Dca017098	87.824005	94.172134	93.407303	43.661289	42.844376	44.086491	36.860767	32.044437	33.19981
gene-MA16_Dca017095	10.593604	9.31478	8.225617	10.867311	9.922169	10.659269	23.379513	19.472136	23.017895
gene-MA16_Dca017542	21.231586	19.946096	19.14674	7.1009	7.290143	6.440936	6.66841	8.632228	8.23482
gene-MA16_Dca017543	1003.0368	1016.44275	976.970886	538.608093	516.569397	539.207825	544.117188	605.725891	594.046509
Dendrobium_catenatum_newGene_8091	7.414061	7.747968	7.077053	1.7174	1.791924	1.578607	3.376051	3.251531	2.439241
gene-MA16_Dca005863	47.741188	49.578747	44.707077	15.218952	12.350163	12.307591	22.541794	25.090645	26.606234
gene-MA16_Dca004802	9.51938	11.649274	9.703609	1.316427	1.261707	0.913555	0.902696	1.088941	1.245925
gene-MA16_Dca019695	75.125748	82.144943	78.112244	22.913813	18.703072	24.307907	37.924274	42.088741	33.441341
gene-MA16_Dca005040	15.939898	12.715366	10.868557	38.378773	34.291241	35.758327	69.507286	69.061081	65.422729
gene-MA16_Dca007766	21.143326	21.672417	22.268068	23.926546	22.443644	23.310469	5.768015	7.17259	7.560472
gene-MA16_Dca000137	10.424858	10.53436	8.826085	4.71084	5.05667	4.50481	5.813351	6.131012	6.813447
Dendrobium_catenatum_newGene_4376	0.844625	1.022921	0.733381	1.1714	1.161775	0.703403	2.97926	1.839139	2.278759
gene-MA16_Dca010278	59.847134	61.09787	65.296745	27.301773	25.511749	28.591875	15.378099	14.813099	16.338175
gene-MA16_Dca008442	12.485148	12.164553	13.456181	26.170343	26.308952	30.901358	31.949396	33.80769	31.330038
gene-MA16_Dca020430	2.573076	0.891956	1.798864	4.928467	3.852433	4.2353	2.355215	3.201347	4.430778
gene-MA16_Dca014563	141.263687	142.549149	149.290054	76.536591	64.850975	73.020454	62.848072	58.492996	57.910126
gene-MA16_Dca003449	2.794512	5.173249	4.744978	4.668564	4.002518	5.012302	12.835231	13.436044	13.914476
gene-MA16_Dca000865	16.731035	20.279312	14.889743	11.992491	10.802541	12.087318	4.620027	5.143311	4.454019
gene-MA16_Dca000864	356.171265	348.501617	342.362946	247.039322	262.872345	246.214203	164.526871	154.064575	164.661743
gene-MA16_Dca002238	124.070663	135.452652	131.111069	164.726105	154.947403	146.387802	309.626953	268.381958	291.693726
gene-MA16_Dca002795	9.404089	12.458814	11.688148	5.083238	4.437993	5.896691	2.551259	4.217676	3.222255
gene-MA16_Dca023186	36.83075	40.733395	35.398613	10.398553	8.500906	7.020401	7.810523	9.699913	9.219337
gene-MA16_Dca000401	2.7908	2.016249	2.03015	1.580627	0.409684	0.828405	3.537653	1.950311	2.507462
gene-MA16_Dca000405	0.843893	0.555815	0.815041	1.845458	1.566114	2.277797	0.591056	2.208493	1.150034
gene-MA16_Dca022589	145.217361	141.351517	140.425049	27.186544	27.589653	28.066252	33.936214	34.073425	32.60733

gene-MA16_Dca017709	6.459543	8.700085	6.394522	2.607797	2.432484	3.23525	3.582202	4.620924	2.961161
gene-MA16_Dca015703	294.904053	286.355408	285.027832	38.54425	42.342373	38.41441	55.841919	38.589367	53.979275
gene-MA16_Dca013286	23.454069	23.513012	24.636414	41.958866	39.261101	42.436211	126.137444	138.53476	138.271469
gene-MA16_Dca013284	117.974083	108.356491	118.699928	56.338573	56.762089	57.762051	57.73785	58.929653	60.792511
gene-MA16_Dca025316	21.942736	19.887474	20.217607	8.857619	8.417972	8.120961	10.532875	11.189109	10.978893
gene-MA16_Dca025317	308.246399	312.852814	304.394409	139.356369	132.07373	138.499207	133.442993	134.019424	134.809769
gene-MA16_Dca021944	0.626966	0.71829	0.929619	1.138972	1.393423	0.532515	1.807639	2.020572	1.172535
gene-MA16_Dca023849	2.745899	1.664696	1.08433	6.898629	6.642865	8.206673	8.478719	9.174899	7.831235
gene-MA16_Dca023840	139.059799	140.179428	137.991226	119.749138	115.894867	124.620651	428.308472	411.509186	412.938751
gene-MA16_Dca001227	118.874138	116.742554	118.899368	91.000359	87.019707	84.778275	53.604866	51.457413	56.490582
gene-MA16_Dca004307	5.74527	6.965784	6.872409	0.342681	0.436812	0.665346	0.246722	0.613253	0.492624
Dendrobium_catenatum_newGene_6681	37.829746	38.529694	34.914265	48.703629	43.400406	47.651005	95.365303	82.899124	93.585884
Dendrobium_catenatum_newGene_9485	12.231526	13.536803	14.969089	12.002028	14.087263	16.47821	33.811478	35.144196	36.254089
gene-MA16_Dca002223	14.119205	14.736834	13.718269	6.299318	5.886376	7.288115	5.826647	6.009469	7.054172
gene-MA16_Dca006212	310.219879	296.640686	305.244446	132.368256	120.107719	128.568192	68.977859	66.364548	73.073189
gene-MA16_Dca024420	1.799758	1.334687	1.07132	2.033068	1.405993	2.025105	3.321623	3.325652	3.750281
gene-MA16_Dca028884	29.745787	32.870499	30.393066	14.971201	14.830001	11.999512	20.545748	26.334974	26.263493
gene-MA16_Dca013918	1.636871	1.722248	2.010994	1.833143	1.113171	2.113604	5.964404	3.542672	3.225649
Dendrobium_catenatum_newGene_10246	36.574425	32.084288	31.602244	38.826621	38.762864	40.092121	18.062002	19.010426	16.301159
Dendrobium_catenatum_newGene_6896	4.061347	3.883842	3.853093	2.369073	2.546541	1.282018	1.788756	1.02808	1.537154
Dendrobium_catenatum_newGene_16379	0.764178	1.86112	1.21318	1.906919	3.200856	2.92389	3.652033	3.244541	3.399273
gene-MA16_Dca027301	19.641991	23.2948	19.666927	2.392794	2.751824	2.213196	1.972332	2.347625	1.9475
gene-MA16_Dca028308	32.162987	35.389187	31.577923	20.286301	16.624035	21.266535	17.166704	15.754163	13.437543
gene-MA16_Dca022742	596.371094	611.403381	576.861816	1312.17944	1364.44617	1315.51428	1987.75122	1947.46008	1967.90503
gene-MA16_Dca007125	2.366061	2.335663	2.168861	3.938386	5.220748	3.809355	5.100739	5.047513	5.228488
Dendrobium_catenatum_newGene_11682	0.953857	1.699575	1.438663	3.935946	2.796978	3.280648	5.710799	6.07691	6.140716
Dendrobium_catenatum_newGene_2406	0.474185	0.870422	0.232152	0.65781	1.202097	1.396468	2.408308	1.630737	1.876001
gene-MA16_Dca019363	68.260811	67.29509	66.696152	139.820312	152.919327	153.874695	77.589088	78.417793	86.383133

gene-MA16_Dca019364	12.525651	10.200985	9.924722	25.609846	23.103218	26.43153	38.681366	43.148491	37.039639
gene-MA16_Dca019365	9.903667	12.814281	9.061179	19.97629	22.27104	25.235363	37.988628	46.21048	37.166042
gene-MA16_Dca018941	2.779143	2.796037	2.566885	1.961569	1.919336	1.59744	3.645815	4.404372	3.666883
gene-MA16_Dca018943	68.760284	78.190254	73.466248	1.658449	1.719885	1.397096	1.536112	0.373449	1.569707
gene-MA16_Dca018942	143.55191	157.508484	145.596176	2.182079	2.89739	2.598882	2.801036	3.820906	2.954283
gene-MA16_Dca018947	112.981575	121.860023	107.121292	46.035286	46.10289	40.166679	29.84202	28.72871	31.370874
gene-MA16_Dca019095	51.015415	53.70166	51.622761	13.422612	11.317801	11.792838	8.158136	7.225897	7.886953
gene-MA16_Dca019090	19.105469	17.533686	16.998959	6.386371	5.253304	6.010742	10.082372	16.250113	17.558378
gene-MA16_Dca005810	51.08984	51.329899	53.080669	28.526728	25.697483	25.992819	27.379236	29.449184	29.891922
gene-MA16_Dca020110	84.121933	79.787941	86.680405	6.180164	4.791572	4.066184	6.826453	6.537255	7.834598
gene-MA16_Dca020114	0.129415	0.167395	0.208827	0.172836	0.222705	0.385567	2.478164	2.537017	1.47593
gene-MA16_Dca006054	3.25876	3.113051	3.412193	5.782629	4.033491	5.465609	9.792004	6.775449	6.502859
gene-MA16_Dca006055	22.155937	21.667221	22.669924	3.469703	3.875115	2.59682	2.757397	2.346769	1.418651
gene-MA16_Dca001587	4.147417	5.410965	4.313241	1.474995	1.570264	1.175011	2.232355	2.373419	2.124902
gene-MA16_Dca006581	13.830433	13.563345	17.597336	0.949396	3.056358	4.221995	5.934007	6.480449	4.548334
gene-MA16_Dca001058	121.196518	127.388687	110.201607	39.656456	35.443806	42.320557	21.706398	21.819609	21.379713
gene-MA16_Dca005772	15.347687	15.67303	13.818261	6.801292	8.743234	7.90607	11.625223	14.164516	12.793894
gene-MA16_Dca021422	56.321716	59.214741	51.460098	18.395948	17.417189	18.875378	19.063139	18.609541	18.609674
gene-MA16_Dca006879	37.685978	33.408272	32.90736	22.249783	21.590269	23.541334	16.16033	16.555544	14.417001
Dendrobium_catenatum_newGene_5690	12.14485	10.979035	11.351357	1.363804	1.117402	1.27648	0.924932	0.980901	1.393851
gene-MA16_Dca003960	2.916697	2.418822	2.616772	2.915501	3.786105	3.92468	7.504369	6.323477	7.854546
Dendrobium_catenatum_newGene_7695	2.002142	1.262742	1.200728	1.354079	0.997792	1.343918	3.461541	3.137723	2.277337
gene-MA16_Dca013031	387.968445	401.759674	389.361145	228.381149	221.415497	232.915939	130.419739	119.614105	119.584915
gene-MA16_Dca011881	13.371692	13.593812	13.212602	3.897443	4.705569	3.484174	5.138651	6.251964	5.801569
gene-MA16_Dca022179	10.315295	8.348456	10.473944	4.502726	3.945321	4.194118	6.62289	8.274059	6.868904
gene-MA16_Dca025185	15.613375	14.152206	15.625376	34.286217	31.297159	34.833321	36.009975	32.966846	35.274242
gene-MA16_Dca024585	5.395671	6.529894	7.484834	14.081185	16.328444	14.30456	21.673643	26.078808	20.881634
Dendrobium_catenatum_newGene_4643	10.523852	10.332323	10.431364	1.50893	0.987744	1.424423	0.505263	0.981464	0.743228

gene-MA16_Dca012433	22.12285	23.803293	26.246895	11.628844	10.903928	9.847974	11.211964	10.405299	11.271423
gene-MA16_Dca012437	5.722546	5.000103	3.771246	2.729433	2.92964	3.529099	1.42157	2.435608	0.465936
gene-MA16_Dca012436	31.867916	30.823168	26.027737	5.457979	5.15859	5.703093	4.389028	2.276654	3.120405
gene-MA16_Dca003622	88.920403	82.552544	92.371964	167.683228	161.796371	172.746353	218.149872	193.306	197.395142
gene-MA16_Dca011303	42.990486	37.747025	40.62606	1.596559	1.73708	1.406895	2.250376	1.844494	2.832672
gene-MA16_Dca000559	58.960167	65.278717	61.291862	9.417553	10.46075	10.933491	13.179098	15.419638	14.256163
gene-MA16_Dca027110	6.869735	3.574907	5.604419	3.37415	2.785325	3.826768	9.273328	6.027191	11.161321
gene-MA16_Dca012147	240.97406	241.843323	242.501587	66.677444	80.230904	79.661674	69.937828	71.85392	62.36837
gene-MA16_Dca017013	8.105238	10.31864	9.125567	1.288033	1.389288	1.811382	0.357723	0.75908	1.221042
gene-MA16_Dca017015	27.140276	27.54492	25.955265	3.501079	2.784559	4.027685	3.61458	3.625206	5.223953
gene-MA16_Dca023346	2.683794	3.755485	2.777139	3.504733	3.618244	4.513456	9.226657	9.063379	7.270691
gene-MA16_Dca023348	11.144642	7.376155	9.595183	3.058092	2.07255	3.692928	2.924834	1.200911	4.782444
gene-MA16_Dca022436	1.951114	2.349435	2.180985	0.910606	0.525982	0.473353	0.748653	0.50112	1.132916
gene-MA16_Dca014099	46.555618	42.320248	42.283203	20.594448	18.788185	23.13513	18.892681	18.61161	17.522102
gene-MA16_Dca014174	0.790552	0.644135	1.09692	1.399988	0.983498	2.914738	4.28725	6.461535	4.449214
gene-MA16_Dca015327	14.537127	15.257203	12.534272	26.011028	23.85342	28.210459	50.059566	47.954765	49.638023
gene-MA16_Dca021479	35.790871	36.181469	37.895088	17.237169	17.617935	17.594517	19.125484	18.997845	20.556261
gene-MA16_Dca004888	0.396739	0.536529	0.530219	1.150078	1.377017	1.095981	0.758915	1.223198	0.959142
gene-MA16_Dca004880	28.159056	28.930391	31.098476	25.180342	22.950039	24.722954	54.696915	60.06599	61.022938
gene-MA16_Dca004884	20.244682	19.595457	19.616186	5.5925	6.820524	6.505082	5.65029	6.041613	5.676148
gene-MA16_Dca020701	1.273255	1.002729	1.461454	1.867199	2.475183	1.524562	4.012625	3.453494	2.725632
gene-MA16_Dca020702	1.122809	1.02053	0.35522	2.768629	1.708546	2.979809	2.179149	3.099635	2.770489
gene-MA16_Dca000369	13.006109	14.398863	14.481671	4.487144	2.237535	3.26932	1.93301	1.090088	2.699417
gene-MA16_Dca001936	0.394592	0.197074	0.235956	0.611582	0.692771	0.803036	1.212495	1.387849	1.002916
gene-MA16_Dca001939	28.825317	28.160242	29.566423	47.633583	45.732155	48.402534	69.441185	72.811295	67.337112
gene-MA16_Dca006626	14.820512	16.247541	15.409216	9.323766	9.668796	10.616704	6.557076	5.896208	5.393519
gene-MA16_Dca006621	173.585999	175.312317	178.581116	319.725616	307.508942	307.827515	516.822998	484.834808	464.68866
gene-MA16_Dca008371	1.131304	0.611605	1.545492	1.613379	1.755622	2.397154	3.603989	2.25912	4.108048

gene-MA16_Dca008376	10.552191	10.070479	11.6743	1.533349	1.703518	2.233333	2.347562	3.046925	2.761553
gene-MA16_Dca004710	14.219271	15.101124	14.083519	10.466098	9.807197	10.138374	6.71639	8.415178	7.064794
gene-MA16_Dca004714	15.828361	20.787931	15.702157	6.179784	5.989933	6.466317	9.249352	8.481	9.135957
gene-MA16_Dca011142	1.766597	1.619484	1.466993	0.112161	0.284809	0	0.219551	0	0
gene-MA16_Dca000712	7.790997	8.245078	7.375819	4.016973	3.868672	4.592533	3.212125	2.960062	2.723654
gene-MA16_Dca002715	36.459621	36.35535	33.870132	3.785872	2.865837	3.878237	2.394835	2.887831	3.213295
gene-MA16_Dca002711	4.833402	5.11335	4.580255	1.418518	1.386413	1.135834	21.204058	13.703982	13.918554
gene-MA16_Dca026553	2.315032	2.363953	2.762639	1.919265	2.023543	1.985936	4.568182	4.302849	5.348912
gene-MA16_Dca026552	0.414954	0.372952	0.352299	0.495858	0.31497	0.741631	0.917931	1.205606	0.960555
gene-MA16_Dca009188	293.190704	303.009766	285.707123	157.809708	154.778534	154.997177	138.638229	127.113274	134.872223
gene-MA16_Dca014253	2.478949	2.756528	2.312917	0.3236	0.567622	0	0.431147	1.536956	0.796036
gene-MA16_Dca024042	55.067253	53.107552	50.82214	24.450638	25.306498	22.29084	25.306644	17.807426	24.169281
gene-MA16_Dca019312	46.436661	52.481453	49.429302	7.402611	9.733523	8.565105	6.200393	6.787878	5.154133
gene-MA16_Dca007022	5.374486	5.676403	5.022113	10.786508	10.396531	9.986692	17.431215	16.073614	15.567329
gene-MA16_Dca017195	16.923332	17.338221	14.914602	9.447532	13.8621	7.78142	32.342957	21.454016	33.339233
Dendrobium_catenatum_newGene_16717	3.78698	3.398111	3.295817	1.100813	0.908534	1.390387	1.800829	1.374064	1.163977
Dendrobium_catenatum_newGene_838	3.235887	3.727936	6.284857	0.657049	2.932599	1.340869	1.183951	1.535733	1.408576
gene-MA16_Dca017225	15.440375	13.614257	14.47936	3.066669	3.668371	3.168108	2.2775	1.771887	3.211225
gene-MA16_Dca017227	286.693268	280.798065	279.253326	128.695511	134.393127	130.436707	26.399809	23.352062	28.558142
gene-MA16_Dca017226	569.204712	570.239197	549.036499	243.785492	237.135025	231.874557	173.618362	157.054184	158.316986
gene-MA16_Dca014751	11.659211	11.474957	12.342747	26.553934	24.603573	24.886868	29.322554	26.237574	27.842461
Dendrobium_catenatum_newGene_6603	0.834069	2.076496	0.839241	2.879559	2.833278	2.663056	2.427713	3.762067	2.700879
gene-MA16_Dca006297	15.871549	14.426865	14.907193	3.901115	3.594667	1.97888	21.246935	18.950907	20.454847
gene-MA16_Dca006299	15.318849	15.632651	19.21715	28.604258	27.903137	32.278545	15.210338	11.728922	13.552584
gene-MA16_Dca005671	25.858927	18.597992	19.637905	2.707162	4.036846	5.310725	3.122942	4.064664	2.740255
gene-MA16_Dca005673	102.186661	101.840164	99.941422	51.171398	44.35915	52.778088	37.691631	37.976639	37.191319
gene-MA16_Dca005675	2.584072	3.091894	2.57459	0.485098	0.186922	0.711108	0.070838	0.174718	0.696606
gene-MA16_Dca001746	7.516424	5.915982	6.81926	0.584298	0	0	0.200993	0.161304	0.312732

Dendrobium_catenatum_newGene_4147	16.333603	14.877012	18.192806	8.150569	6.020488	7.269232	14.048215	12.489934	9.625622
gene-MA16_Dca008654	5.43043	5.837926	7.244332	2.490291	2.86381	4.57375	2.143005	1.446081	2.388592
gene-MA16_Dca008650	7.374925	5.148594	5.079777	8.148741	10.386271	11.754857	17.787508	16.604307	17.106533
gene-MA16_Dca008651	8.50402	7.421285	7.51268	19.135859	21.296791	19.928041	27.519829	20.000147	25.025364
gene-MA16_Dca028384	6.059481	5.407528	5.099203	14.964396	12.42443	13.935643	7.926609	10.032588	6.365476
gene-MA16_Dca028383	4.284609	4.644056	1.928467	10.421634	7.065846	9.412271	14.6238	13.720694	14.190303
gene-MA16_Dca009688	12.671123	13.23269	13.24662	3.603796	4.122577	3.351007	5.798536	5.354609	6.132974
gene-MA16_Dca016133	0.679019	0.746094	0.671676	1.761949	1.7967	1.928486	3.168128	2.547182	2.714661
gene-MA16_Dca002504	1.872508	1.69751	1.722737	4.250483	4.478052	4.111605	0.933039	0.629629	0.50162
gene-MA16_Dca002508	33.494946	32.82983	31.144764	73.590393	66.271301	68.464333	133.554901	138.958008	136.36853
gene-MA16_Dca010990	49.854259	54.32539	60.302334	21.156185	26.268976	23.264055	22.89802	23.554396	22.304888
gene-MA16_Dca010994	1.799202	1.875163	0.840763	2.1319	3.242263	3.064625	4.672948	4.434678	3.896993
gene-MA16_Dca003291	48.899948	51.430534	51.774353	64.222328	66.544983	64.528229	149.852417	154.891403	151.238327
gene-MA16_Dca020193	1.243222	1.607905	0.445278	6.758716	6.8732	6.970639	8.414783	6.682477	6.76736
gene-MA16_Dca001508	4.742486	5.42358	5.072283	2.267046	2.239981	2.583659	2.303321	2.130666	3.121894
gene-MA16_Dca007835	5.015805	3.890224	3.42166	3.00961	2.032529	2.506254	1.847016	0.795465	0.517765
gene-MA16_Dca022903	57.521767	57.36525	60.253155	10.491085	11.548994	10.268211	15.226973	13.02969	10.813509
gene-MA16_Dca022908	1.972287	2.562442	3.000201	6.76486	6.166434	4.321972	10.368961	9.558138	10.287991
gene-MA16_Dca004022	39.483372	37.433239	37.187134	13.0191	11.70476	13.925997	18.797234	16.719692	17.170469
Dendrobium_catenatum_newGene_5567	46.270516	47.356622	46.887139	29.180322	31.129727	35.343146	18.134115	17.496327	17.763353
Dendrobium_catenatum_newGene_6031	0.610297	0.832447	0.94913	0.05586	0.332617	0.211791	0.169966	0	0.249534
gene-MA16_Dca009337	432.112823	432.682709	431.98819	166.213989	158.006409	151.501678	181.615479	169.014862	165.40918
gene-MA16_Dca009330	2.117699	2.507847	2.318636	1.079006	0.808099	0.786161	1.239374	1.496228	1.865668
gene-MA16_Dca009331	4.071204	4.346915	3.832574	1.302202	0.99176	1.92303	1.749347	1.791452	1.965689
Dendrobium_catenatum_newGene_2644	1.19461	1.160322	0.983538	2.477818	2.068367	1.348888	3.066519	3.959955	2.885747
gene-MA16_Dca012925	338.937561	340.763336	332.648285	127.966248	120.375153	121.551994	132.049759	129.198105	133.813995
gene-MA16_Dca004519	21.485643	20.066017	24.029675	6.627372	4.680288	5.784935	3.785383	5.463469	7.029032
gene-MA16_Dca009807	13.286573	13.453061	15.382732	6.128638	4.313015	3.839283	6.352998	4.344868	5.667834

gene-MA16_Dca010389	190.427155	188.850845	190.556396	103.43541	105.581429	108.106537	99.254578	88.955399	94.854996
gene-MA16_Dca010381	29.91671	31.641933	28.720318	9.577075	10.795635	10.126866	12.675579	11.530242	12.174536
gene-MA16_Dca011386	9.22683	8.309007	6.58231	11.480164	13.518578	13.830156	15.208164	20.750076	18.814367
gene-MA16_Dca011387	4.555918	3.870348	4.136223	7.353966	5.885212	6.466909	15.447841	11.953815	14.370323
gene-MA16_Dca011521	16.605398	17.877737	17.516624	15.204962	15.78942	15.647523	32.167774	33.512798	29.799114
gene-MA16_Dca011525	109.857269	115.74826	114.875	66.982658	63.511078	74.531677	57.832111	58.478481	56.47913
Dendrobium_catenatum_newGene_10863	30.962093	30.473103	30.107655	637.966431	645.266785	617.107178	184.702225	193.666199	206.992111
gene-MA16_Dca023691	2.477328	2.842834	2.315507	1.456902	1.826496	1.214204	0.556409	0.76207	1.347397
gene-MA16_Dca002334	60.560341	55.332672	54.218975	54.610889	54.805901	55.72961	139.706345	137.007004	141.404892
gene-MA16_Dca018826	5.989149	5.133986	4.272769	3.578211	4.309851	3.086959	0.718788	1.645262	0.988657
gene-MA16_Dca021169	76.334763	78.827812	72.71209	122.186722	121.177483	125.55703	180.796539	169.632675	181.063629
gene-MA16_Dca026399	11.256048	12.279016	10.177967	3.280564	2.993413	3.12797	3.395442	2.942297	3.016623
gene-MA16_Dca007208	38.696022	37.547348	38.30143	12.563685	11.448672	12.926757	17.257891	16.320759	15.013583
gene-MA16_Dca007203	25.838308	28.776239	26.663561	9.405437	11.27202	9.618659	20.577143	17.261688	21.298862
gene-MA16_Dca027781	3.187859	4.442803	3.711377	4.970828	5.248149	5.182779	9.635016	11.109902	11.062448
gene-MA16_Dca020789	23.904518	25.008566	23.691689	14.196408	11.599953	12.69495	12.502419	12.069514	12.980029
Dendrobium_catenatum_newGene_8866	5.95702	5.183433	6.175613	6.769627	4.108757	4.655569	14.993974	15.076976	14.279924
Dendrobium_catenatum_newGene_8864	0.718933	0.475422	0.845867	1.50042	0.9799	0.85016	3.168078	2.213622	2.26751
gene-MA16_Dca027258	1.519352	1.527159	1.040902	1.185646	1.460606	1.373154	0.245205	0.174345	0.728377
gene-MA16_Dca004797	20.540874	22.052542	19.379583	18.203794	21.202944	18.068947	11.658072	8.896746	11.448445
gene-MA16_Dca005544	103.175636	108.874039	113.431564	81.864479	86.460762	88.099007	44.350212	37.21064	34.742123
gene-MA16_Dca005541	5.451091	6.266978	6.867193	2.319214	4.216627	4.149436	0.094084	0.629933	0.655237
gene-MA16_Dca005548	1.500324	1.103675	1.256046	0.492389	0.577558	0.610987	0.299375	0.399462	0.469643
Dendrobium_catenatum_newGene_5796	0.340161	0.142787	0.312992	0.284627	0.571548	0.796888	0.629509	0.535073	0.939518
gene-MA16_Dca009109	8.204052	7.438858	9.164583	12.767425	10.656412	14.231641	18.566662	19.087027	20.377611
gene-MA16_Dca009105	12.211847	10.810124	10.218179	22.13866	21.398134	21.209909	51.362804	49.701263	48.651272
gene-MA16_Dca022637	41.218739	36.83115	33.509521	70.066124	71.939301	78.682785	104.656616	103.885292	102.105263
gene-MA16_Dca022632	268.442139	270.596619	278.492645	212.737946	210.922516	197.697998	125.192284	131.284119	121.258148

gene-MA16_Dca000219	7.912042	8.321219	6.936212	6.846827	6.954665	7.072478	16.12532	15.163281	13.870968
gene-MA16_Dca020547	20.064466	18.297281	19.918449	40.229187	38.627697	39.134834	18.308447	17.789869	20.273083
gene-MA16_Dca016994	1.337536	1.770778	1.342423	1.40841	0.919413	1.005578	2.469583	2.478738	2.573111
gene-MA16_Dca012061	1.590216	1.617759	1.694777	1.264367	0.833639	1.965448	3.990613	4.24073	2.577487
gene-MA16_Dca003741	53.766045	57.750851	56.444141	30.499695	27.419138	27.987007	18.846956	16.16164	17.384344
gene-MA16_Dca003740	138.512939	125.199348	133.066315	22.434008	19.731934	27.567989	13.235878	8.967422	10.654991
gene-MA16_Dca003746	620.610718	625.69043	579.252869	137.918289	150.192963	143.621399	267.180664	243.373566	258.896759
gene-MA16_Dca004616	79.231461	77.771713	83.910797	18.01272	17.605623	17.640583	21.254927	20.807652	22.710535
gene-MA16_Dca023222	0.380548	0.219152	0.905424	1.146168	0.995805	2.584245	2.369824	1.526944	2.060974
gene-MA16_Dca026058	1.228872	1.624708	1.951895	2.2877	1.77776	2.069333	3.865813	3.901593	2.948307
gene-MA16_Dca026059	8.309642	6.989922	7.788571	3.266683	2.667214	0	0	0	0
gene-MA16_Dca018164	3.168189	2.531288	3.093753	1.951891	1.631802	0.331265	0.407839	0.723401	0.335751
gene-MA16_Dca018169	0.336216	0.347771	0.434675	0.308358	0.499668	0.898464	1.570533	0.94411	1.517426
gene-MA16_Dca017806	1.441475	1.433302	1.574114	1.629224	1.464024	1.102548	4.702778	3.841516	3.495003
gene-MA16_Dca017808	4.891378	5.272705	6.734042	1.407882	1.661679	2.18309	1.16336	1.048175	0.943145
gene-MA16_Dca003355	61.064053	60.088791	60.913208	24.266935	23.335629	24.812113	25.593554	25.603508	25.096245
gene-MA16_Dca018787	150.052353	156.670364	154.798203	48.947021	46.995823	49.557449	52.10648	56.539761	58.982021
gene-MA16_Dca015405	3.839278	3.121476	4.052415	5.629965	4.525554	4.184025	10.806495	9.286105	9.001454
gene-MA16_Dca004964	50.773663	51.493435	49.363598	56.16877	55.756168	56.627529	29.099131	27.303028	26.323673
gene-MA16_Dca016536	23.148073	19.985199	19.998859	14.069573	16.1017	16.697752	9.051404	9.883083	11.256674
gene-MA16_Dca008522	13.52972	15.134271	16.658674	7.827836	7.059492	6.903425	7.398486	7.387132	7.704072
gene-MA16_Dca008524	39.11869	36.723831	35.881069	16.872536	15.30174	20.328753	9.157904	8.182835	9.066184
gene-MA16_Dca004187	250.582565	254.501175	235.219772	75.019577	74.477516	71.026268	55.440559	53.287956	53.590008
Dendrobium_catenatum_newGene_3943	32.395264	29.546268	27.715122	1.990839	3.032319	3.669182	7.195136	6.347939	7.077488
Dendrobium_catenatum_newGene_14793	3.302599	3.617644	3.156417	0.889095	1.158593	1.158985	1.227928	0.900504	1.509427
gene-MA16_Dca005155	10.680533	11.294213	11.252257	10.997765	10.053684	10.889407	23.11101	22.822931	23.550709
gene-MA16_Dca011775	11.861973	12.372693	13.664019	10.479477	11.045069	10.384862	20.397272	23.13929	24.108261
Dendrobium_catenatum_newGene_11484	0.395311	0.280517	0.282251	0.798968	0.89139	0.606646	0.706183	0.858433	0.828172

gene-MA16_Dca000984	12.046616	10.69225	10.758364	21.680738	19.648659	24.889448	30.340487	31.446791	28.83564
gene-MA16_Dca002587	2.945536	2.435468	1.529521	0.961722	1.134786	0.954629	1.805184	1.732971	2.822596
gene-MA16_Dca002585	8.888309	9.555296	11.315583	3.118347	2.946065	3.695901	2.976061	2.140225	3.329809
gene-MA16_Dca002581	6.22499	5.873556	6.4408	2.856209	3.555371	2.426485	2.451419	2.257113	2.679686
gene-MA16_Dca003182	6.938139	8.405904	8.297551	17.822403	18.513823	21.741367	21.545555	23.547281	21.062712
gene-MA16_Dca016207	1.662644	0.96543	3.329652	5.040628	6.490105	3.017782	6.121908	4.587061	4.674232
gene-MA16_Dca010914	4.803226	4.938495	4.865351	1.83872	1.947117	2.032127	1.406345	1.808136	1.561928
gene-MA16_Dca023022	52.605427	50.777657	47.222347	3.184928	3.143779	3.723632	7.711958	5.183814	4.660544
gene-MA16_Dca023023	9.773648	9.98713	8.764405	4.709415	4.958795	4.964248	5.226629	6.021498	6.431976
gene-MA16_Dca023028	4.381997	3.482604	3.994059	4.232654	3.492991	3.640601	2.145246	1.812296	1.293157
gene-MA16_Dca026212	4.402304	5.080158	4.759422	1.196963	0.841933	0.948463	1.463587	1.30083	2.219161
gene-MA16_Dca008950	281.048279	269.521973	274.793976	141.662766	139.197144	144.123276	127.889679	137.011124	132.143066
gene-MA16_Dca008956	9.482811	8.778006	7.347914	1.123171	1.129743	1.543948	2.266826	1.421035	1.745289
Dendrobium_catenatum_newGene_11021	6.073044	4.571774	5.28524	2.366449	3.041282	3.000591	1.769427	2.160444	2.679625
gene-MA16_Dca025741	9.316852	8.06892	8.074454	8.787478	8.968742	9.293312	28.361908	31.327738	29.668642
gene-MA16_Dca025746	3.083632	4.949822	3.015355	0.247228	0.402666	1.093253	1.288863	1.06367	1.511811
Dendrobium_catenatum_newGene_3201	829.854919	838.771545	844.60321	992.714111	1004.13519	967.035095	486.996155	465.26767	504.26358
Dendrobium_catenatum_newGene_475	1.191868	1.176204	0.902768	0.098199	0	0	0	0	0
Dendrobium_catenatum_newGene_14961	220.850342	215.350052	224.248428	66.137932	61.046825	65.005074	39.098591	43.076622	46.776985
gene-MA16_Dca021535	5.002735	4.94929	4.383398	3.060972	1.897743	2.480854	2.023452	2.473203	2.292157
gene-MA16_Dca021537	2.309029	0.768087	1.602207	2.770319	2.451308	4.91828	8.061434	5.901987	6.437728
gene-MA16_Dca021538	0.533144	0.652215	0.633252	1.360695	2.902395	4.299426	3.87544	4.419109	3.890347
Dendrobium_catenatum_newGene_13538	0.904537	1.025539	0.867832	0.691025	1.256346	2.034703	2.054552	2.319239	2.044779
gene-MA16_Dca020665	20.396469	18.781202	20.599724	1.665408	1.534353	1.449877	1.660126	1.985483	2.731348
gene-MA16_Dca015937	8.105843	9.14963	8.648251	4.881007	3.966209	4.781065	3.642508	4.098711	3.504838
gene-MA16_Dca026619	15.009439	15.074396	16.428493	54.413204	55.332443	54.000141	81.387177	65.675522	68.485168
gene-MA16_Dca022981	37.999928	31.575642	36.006073	0.659113	0.401759	0.507419	0.172043	0.21976	0.159438
gene-MA16_Dca026157	25.01539	25.028999	28.571533	36.316647	36.289417	36.887951	16.589848	17.061071	16.135218

gene-MA16_Dca026154	0.941584	1.108424	1.231548	0.451383	0.565415	0.396627	0.297522	0.520883	0.647239
gene-MA16_Dca024579	22.59943	20.798241	22.981979	36.06654	40.024704	36.056789	54.559601	52.511566	55.831367
gene-MA16_Dca018080	1.986906	2.200182	2.159846	2.117126	2.006044	1.932376	6.490628	5.355561	5.949818
gene-MA16_Dca018083	9.175997	8.879493	8.925755	18.971003	16.702528	16.702681	99.987808	96.470261	99.787262
gene-MA16_Dca017307	1.422802	0.963986	1.533787	4.157722	3.158812	3.60165	4.102633	5.685795	4.10014
gene-MA16_Dca017305	147.859299	144.685669	150.835144	22.663399	24.873886	23.708832	12.679414	11.86449	16.611624
gene-MA16_Dca017300	50.867798	45.747898	48.417606	12.73991	12.039453	12.208951	11.49737	12.235677	13.148554
gene-MA16_Dca008161	1.482421	2.749224	2.036666	1.506406	1.231917	1.889972	0.812955	1.169441	0.45129
gene-MA16_Dca019762	2.097382	2.838408	1.905725	1.003785	2.033949	0.900018	1.255179	1.027328	1.105162
gene-MA16_Dca019760	1.394213	2.360732	1.726904	1.550098	1.204175	1.415071	0.384242	0.557396	0.391648
gene-MA16_Dca014651	1.250719	1.26843	1.0523	2.530801	2.635226	4.469376	2.397466	3.014045	2.989717
gene-MA16_Dca014650	4.188948	5.235054	6.196794	2.934768	2.014876	2.378103	2.219059	2.341089	4.168008
gene-MA16_Dca005916	37.649014	32.721233	37.739681	17.98061	15.787836	22.918228	19.227972	13.436557	15.204742
Dendrobium_catenatum_newGene_138	1.881761	2.26905	2.276436	2.925819	3.44457	3.443661	5.211136	4.8324	5.094697
gene-MA16_Dca000681	17.925648	16.452829	20.406046	4.696566	3.480621	3.856394	6.00957	6.083995	5.791876
gene-MA16_Dca006394	4.538867	3.379072	3.350135	0.538741	0.334131	0.42184	1.249118	0.729447	0.291593
gene-MA16_Dca007660	34.012054	34.305973	37.284004	53.042675	50.326981	51.872452	80.090454	84.83847	78.676003
gene-MA16_Dca005136	0.258136	0.59077	0.543223	0.894766	1.023228	0.988758	6.053782	3.882831	6.334968
gene-MA16_Dca005131	51.269169	46.435955	44.565361	21.398746	18.276859	20.235598	23.16415	20.915171	18.97677
gene-MA16_Dca005139	1.496919	1.25425	1.335824	0.498699	0.715924	0.545584	0.530728	0.7876	0.558756
gene-MA16_Dca009280	7.268161	4.973699	7.11188	2.206421	2.903573	4.361229	3.117995	2.246644	2.027409
gene-MA16_Dca008284	15.113798	15.477816	15.63948	23.000114	21.284367	22.67597	42.563789	39.027668	39.70882
gene-MA16_Dca008283	1.825881	3.298188	2.451619	0.116216	0.15895	0.212385	0.283559	0.529176	0.344601
gene-MA16_Dca004669	23.15634	21.108879	21.727648	2.307461	2.852893	2.767866	0.331215	1.11758	0.73052
gene-MA16_Dca012234	2.153598	2.253254	1.956193	4.622749	4.778148	5.450381	4.76728	4.230462	5.024086
gene-MA16_Dca003575	1.778419	2.143231	1.920072	8.353892	7.897436	8.440053	19.084394	19.479528	22.592333
gene-MA16_Dca003571	0.981816	1.270934	0.746792	1.671485	1.197552	1.450743	2.035358	1.876513	2.765464
gene-MA16_Dca003573	39.865841	41.702011	40.855026	18.221605	17.616261	19.381498	21.993155	22.098017	24.586714

gene-MA16_Dca002623	775.769226	771.407654	766.891357	428.198334	416.689301	434.531311	392.531097	386.379608	389.469421
gene-MA16_Dca002620	53.648602	53.124039	65.540665	44.288387	39.568016	44.103062	10.04115	9.145689	4.427837
gene-MA16_Dca010899	9.589836	8.715882	8.968646	17.977455	19.838943	19.790939	24.614115	23.78463	26.496687
gene-MA16_Dca010898	7.560781	7.144409	7.964794	4.635469	6.096267	4.888837	5.123167	2.809116	3.171344
gene-MA16_Dca026395	59.051216	55.526104	54.899601	58.560089	55.320946	58.183849	27.879107	27.14797	29.468653
gene-MA16_Dca012791	8.616237	6.430765	7.664006	0.279048	0	0.304419	0	0.19708	0
gene-MA16_Dca017670	21.595058	21.23909	20.433939	29.615858	33.870739	30.544748	54.618168	49.588749	50.565937
gene-MA16_Dca018537	83.298851	78.578568	82.609642	17.00823	16.423435	17.322107	15.226475	12.88718	13.989239
gene-MA16_Dca011852	12.374143	12.789123	14.322949	7.052669	7.032789	8.323626	6.336197	7.0222	6.938247
gene-MA16_Dca011855	1.921307	2.235408	2.571312	1.166036	0.948264	2.079358	3.025913	3.454296	4.003262
gene-MA16_Dca019667	3.566111	3.314985	4.012875	0.253229	0.343725	0.876655	0.763141	0.198514	0.61675
gene-MA16_Dca025159	2.772727	3.812344	2.819765	5.493402	5.859095	4.892287	7.190294	8.112615	6.904747
gene-MA16_Dca025157	5.456297	2.183348	4.457909	7.851939	7.929654	5.621337	11.472206	10.152778	7.586535
gene-MA16_Dca025154	21.572598	20.134426	21.554804	46.4967	44.85202	48.063015	48.096428	46.124767	47.532299
gene-MA16_Dca021703	6.123733	8.623918	6.237612	1.233179	1.502605	1.517042	13.995212	14.141547	15.066009
gene-MA16_Dca020252	10.242846	10.420179	7.693636	14.208023	13.156881	13.569602	34.609985	32.175949	29.771364
gene-MA16_Dca001422	0.785553	0.624005	0.624993	0.159336	0.310474	0.338336	0	0.095757	0.12635
gene-MA16_Dca001398	17.303577	16.950123	19.958443	3.255892	5.523467	4.75195	4.985157	4.279757	2.371542
Dendrobium_catenatum_newGene_9883	6.6036	6.903018	6.880022	13.418241	14.433291	10.798316	16.340445	14.334111	15.00515
gene-MA16_Dca020473	5.434124	4.794911	4.248579	11.354893	11.233905	9.00861	13.474135	15.495529	14.000585
gene-MA16_Dca025835	53.777298	53.956879	52.68248	26.068182	27.131521	25.824354	22.211891	24.987337	23.379013
gene-MA16_Dca025831	5.483449	7.727384	6.539508	6.606318	4.497915	5.193154	1.707111	2.796938	2.507169
gene-MA16_Dca017533	1.440617	1.34686	1.339276	2.786288	3.075224	3.41313	4.191152	4.908551	5.002286
gene-MA16_Dca017538	52.721638	50.872593	46.635967	19.549225	15.055257	19.16095	18.139734	15.457332	19.802292
gene-MA16_Dca004278	81.722542	79.43428	79.403473	26.965189	31.128378	28.764345	30.490128	31.517988	28.745462
gene-MA16_Dca004271	3.666578	5.222767	5.718707	2.681684	2.474647	2.195222	5.483006	4.811867	4.101082
gene-MA16_Dca005895	23.663664	22.224266	20.894342	11.844321	11.358996	9.674385	8.936672	9.664291	10.524469
gene-MA16_Dca003802	11.808496	10.908616	11.709813	6.113776	5.40802	6.613733	2.864099	4.309737	3.202031

gene-MA16_Dca012802	49.726414	42.722313	47.128063	12.399408	12.595431	13.246257	11.808582	10.407417	12.219646
gene-MA16_Dca012803	67.648346	63.630859	59.004589	8.195557	9.82945	10.897196	15.295659	14.058647	15.744094
gene-MA16_Dca002860	16.001257	18.782188	19.230379	9.501824	8.606994	13.231204	8.602358	9.053217	8.493037
gene-MA16_Dca001987	16.615316	19.002436	17.983124	42.983913	37.956192	43.914375	45.431759	49.414265	45.120472
gene-MA16_Dca000292	0.376012	0.695453	0.806149	0.505286	0.571622	0.57236	2.43259	2.631491	1.974286
gene-MA16_Dca011640	102.539597	99.49929	96.500511	37.968014	40.484161	39.44014	43.59824	39.676754	42.714462
gene-MA16_Dca011643	0.26468	0.417448	0.264232	0.564739	0.359465	0.175339	1.595684	1.074156	1.482917
gene-MA16_Dca016917	5.549993	3.772764	5.62362	6.435235	5.822136	6.169141	11.876484	15.565719	12.431194
gene-MA16_Dca000833	173.475388	184.585327	178.096848	371.00766	374.812897	365.622131	173.539322	173.328201	172.23764
gene-MA16_Dca022869	9.922561	10.76766	14.178671	9.443676	9.197062	10.472768	2.444084	1.49367	1.195828
gene-MA16_Dca022866	4.881352	4.560972	5.483132	0.942648	0.91034	1.151329	1.82798	0.799745	1.445464
gene-MA16_Dca014425	26.846737	26.724049	25.110294	11.965408	9.265494	9.540146	11.77917	12.131122	13.263119
Dendrobium_catenatum_newGene_2248	2.153857	1.943391	2.951354	6.089052	5.199252	4.366461	0.844552	1.522504	2.357197
gene-MA16_Dca025580	36.725067	39.491703	36.741753	10.657248	11.43671	11.763526	5.230668	4.741323	3.985659
gene-MA16_Dca025582	29.194132	25.652794	26.210999	7.062943	7.341247	5.79966	1.906915	3.237309	2.137748
gene-MA16_Dca017732	1.781437	1.926695	1.046863	4.521096	6.31	4.226596	10.514519	8.830119	12.436638
gene-MA16_Dca026580	11.946297	7.17407	10.147101	5.21081	8.503821	6.622519	3.384559	2.993426	2.506061
gene-MA16_Dca026583	95.386871	96.53083	94.631012	6.694561	6.925814	5.063516	9.982175	9.434473	9.11797
gene-MA16_Dca026586	30.061285	28.431156	30.512583	12.750278	12.09146	13.483595	8.348612	10.218686	8.192905
gene-MA16_Dca024988	26.313919	24.96212	25.166759	0.652748	0.769044	0.473406	0.832314	1.234253	1.064815
gene-MA16_Dca018701	65.631889	66.984024	64.184792	46.956188	38.50349	42.618168	20.38298	21.212233	18.119148
gene-MA16_Dca018706	27.65461	28.555227	29.120367	6.321605	5.176044	4.938226	9.029126	8.645929	9.555736
gene-MA16_Dca017884	34.759724	31.432516	32.558895	12.266813	14.257248	14.038662	12.190987	11.189388	10.09029
gene-MA16_Dca015480	29.492636	29.079523	28.070547	11.000682	11.231652	11.282557	10.203434	10.487086	11.559724
gene-MA16_Dca023872	2.191588	2.352776	2.329285	0.242583	0	0.227131	0.574347	0.455963	0.152916
gene-MA16_Dca006745	47.678394	50.615242	46.968277	16.560797	19.205782	17.854197	19.335886	15.628779	18.448246
gene-MA16_Dca004455	1.520148	1.496459	1.577271	1.387141	1.251305	0.868043	3.34068	2.830062	3.4738
gene-MA16_Dca007908	114.829315	124.446167	113.445107	56.673923	45.055237	47.883526	41.277222	35.050808	34.731136

gene-MA16_Dca007907	73.209122	70.779602	73.775345	33.215347	33.394207	34.185577	45.228886	48.009422	44.467751
gene-MA16_Dca009029	17.732328	18.895893	17.051218	10.446021	8.371665	9.990497	9.835567	7.950177	7.550739
gene-MA16_Dca009022	0.328861	0.537625	0.305003	0.608723	0.406595	0.473897	2.735984	1.729459	1.832688
Dendrobium_catenatum_newGene_7273	8.68529	5.943724	5.776397	0.374222	0.789803	1.628186	0	0	0
gene-MA16_Dca006226	62.904308	61.165569	65.480186	147.20726	148.976044	149.026169	232.276627	222.756912	207.027252
gene-MA16_Dca008048	27.115355	26.065283	27.748772	8.135747	7.456085	7.416761	5.516984	6.718505	5.653889
Dendrobium_catenatum_newGene_4774	14.466169	13.18437	13.419503	11.270731	10.10604	12.934822	60.020817	56.246582	55.371387
Dendrobium_catenatum_newGene_4198	0.674328	0.66777	0.708118	0.071284	0.200539	0.157447	0.147147	0.127151	0.227633
gene-MA16_Dca010727	22.294064	22.523016	21.944571	37.570084	37.638756	34.703735	58.817715	54.597443	57.43515
gene-MA16_Dca000315	69.333481	62.947418	64.027328	101.089325	94.305374	90.688057	175.901993	160.338882	169.717499
gene-MA16_Dca000318	1.876339	2.035222	1.478069	3.983938	5.344734	4.686683	4.474294	4.884506	6.326713
gene-MA16_Dca016893	17.521496	11.697157	11.576379	0.258996	0.665013	0.387142	0.06494	0.269345	0.684256
gene-MA16_Dca012387	5.816489	6.669765	6.087749	11.299187	10.922129	9.983596	13.931186	13.326282	16.897434
gene-MA16_Dca012383	0.973262	0.945559	0.942774	0.309349	0.603872	0.54051	0.23807	0.370192	0.174656
Dendrobium_catenatum_newGene_6849	0.720615	0.923685	0.576969	2.048707	2.008895	2.908053	5.121625	3.260192	5.362806
gene-MA16_Dca023044	23.925337	26.241669	24.379482	1.092429	0.646706	2.091541	1.062676	0.67846	0.652298
gene-MA16_Dca018007	13.588823	11.068469	13.415639	12.083447	12.08165	11.465227	6.956115	4.295202	6.912992
gene-MA16_Dca027828	49.967045	45.007175	41.888012	18.325441	15.491345	18.852451	19.272757	20.429522	17.797857
gene-MA16_Dca018008	0.983751	1.149871	0.728191	1.072084	1.158153	1.089968	1.891995	3.258769	3.880205
gene-MA16_Dca025468	5.803803	5.985966	7.232476	1.588065	2.498175	2.133137	2.17	1.941171	1.488929
gene-MA16_Dca026270	9.993881	8.911554	9.210784	4.74985	5.234116	4.451981	4.659154	5.086078	3.270313
gene-MA16_Dca026271	28.214205	25.66715	30.09549	7.467315	7.694898	9.681581	19.153473	16.624201	17.547844
gene-MA16_Dca007373	7.828353	6.77442	7.344839	8.445463	9.172306	9.219067	24.689083	19.902998	21.726036
gene-MA16_Dca005205	333.865631	337.604736	355.359161	405.327728	443.105988	385.555847	754.464844	712.511353	717.836487
gene-MA16_Dca005201	4.994581	5.038327	4.696178	2.626733	2.071766	1.82932	2.158587	2.143187	2.252259
gene-MA16_Dca005202	7.93189	7.006021	8.038356	1.456233	0.997255	0.418914	1.68708	1.893201	0.967601
gene-MA16_Dca005203	5.398587	6.793362	5.192727	8.030278	7.438089	6.556203	15.923985	18.370541	17.090956
gene-MA16_Dca004559	40.924175	44.937004	40.832088	26.562248	24.141169	25.596663	21.420622	23.429195	18.871725

gene-MA16_Dca000666	1.95294	1.236512	1.50028	2.26848	1.770129	2.27734	0.338793	0.677667	0.638846
gene-MA16_Dca016567	16.170963	16.042704	14.645557	7.568172	5.610789	4.012467	2.736414	2.528686	3.325399
gene-MA16_Dca016562	60.532925	61.114227	65.767952	34.56731	34.850548	34.442852	26.183815	23.930889	23.311895
gene-MA16_Dca006843	0.293437	0.249773	0.11214	0.966231	0.895965	0.648835	0.717031	1.214205	0.948781
gene-MA16_Dca006848	17.374477	16.574537	16.294481	9.224256	6.498737	8.428246	6.888745	9.650021	7.779385
gene-MA16_Dca026311	2.353406	3.139899	1.938091	7.426598	7.58729	8.487756	22.754705	21.48448	21.373028
gene-MA16_Dca026313	1.279395	1.615307	1.094363	0.551443	0.14268	0.368135	1.204965	0.670304	1.151297
gene-MA16_Dca026314	2.200649	1.658944	2.392901	1.840595	1.627562	1.426123	0.932223	1.010796	1.044195
gene-MA16_Dca022127	2.466661	1.602232	2.829327	0.6392	0.975168	1.212754	0.947722	0.930315	0.680396
Dendrobium_catenatum_newGene_3368	7.606443	6.565853	6.643271	5.840981	4.775239	6.093455	32.885269	30.25774	32.306313
gene-MA16_Dca013042	17.101849	12.075741	14.477558	10.592157	8.209154	9.537145	27.872566	30.203127	32.344887
Dendrobium_catenatum_newGene_3094	4.121142	5.134743	3.69085	1.711601	2.014982	2.117712	1.68023	1.50034	2.888032
gene-MA16_Dca010713	29.808897	30.632195	29.073433	2.954369	3.772515	3.370339	1.522762	2.632355	1.567225
gene-MA16_Dca024205	46.509335	49.251495	49.347343	17.493023	19.029108	17.08046	15.267095	14.450781	16.22654
gene-MA16_Dca010717	0.064967	0.393134	0.267347	0.275037	0.375072	0.593669	2.481414	1.253879	1.665245
gene-MA16_Dca010027	0.566818	0.394807	0.286698	1.069445	0.883535	0.740781	0.827878	1.299658	0.745788
gene-MA16_Dca020612	1.415801	1.016525	1.36912	3.355206	3.17635	3.131027	2.608966	2.761514	2.535553
gene-MA16_Dca020611	0.103764	0	0.026301	0.263036	0.145171	0.343488	0.773376	0.950696	0.830052
gene-MA16_Dca011375	34.579147	37.341068	40.054039	20.934172	18.756123	18.021402	13.656475	12.684677	13.539487
gene-MA16_Dca011372	8.962874	10.232101	8.653861	4.493991	4.34422	5.739322	5.2515	4.939961	5.19798
gene-MA16_Dca017028	14.987557	15.168075	14.570778	0.141576	0	0.360416	0.184119	0.167861	0.343006
gene-MA16_Dca025577	164.15329	172.054123	174.54425	100.462791	93.701523	81.632446	63.316864	56.331814	57.669128
gene-MA16_Dca019149	4.412338	5.312296	5.548419	3.330229	3.501252	2.840692	3.279002	2.37326	2.165489
gene-MA16_Dca019146	19.132715	20.423435	19.86665	3.350146	3.176776	2.520422	3.18361	4.140141	3.651921
Dendrobium_catenatum_newGene_19	18.896486	18.19546	21.3289	36.274045	41.610347	42.955546	82.02708	100.276351	81.75739
Dendrobium_catenatum_newGene_1141	4.22686	5.016624	5.580982	2.174515	1.964	2.658352	4.670833	4.049426	5.116714
Dendrobium_catenatum_newGene_9798	3.492942	3.346261	3.441523	0.694141	0.654042	0.517831	0.446231	0.736717	0.678797
gene-MA16_Dca015312	1.989271	2.337277	2.104013	4.991838	3.375837	4.387923	5.804266	4.425735	4.753594

gene-MA16_Dca004800	33.183052	36.042561	30.755207	11.311796	11.398405	13.739344	10.201384	9.215072	10.824841
gene-MA16_Dca008413	8.990926	9.847887	9.718213	20.267145	21.367393	20.049978	16.92211	20.271715	17.257992
gene-MA16_Dca010225	7.044063	9.200704	9.810579	4.897408	3.950996	4.711358	4.943923	4.315865	4.26298
gene-MA16_Dca005591	12.962083	10.721955	11.754308	5.769672	6.253953	5.36554	8.730793	7.10422	7.461257
gene-MA16_Dca005593	3.533913	4.410906	3.197783	0	0	0	0	0	0.187286
gene-MA16_Dca005598	4.970392	3.678752	4.593951	14.74373	11.359752	13.571947	17.672522	16.186235	21.478209
gene-MA16_Dca003295	0.510582	0.302555	0.23639	0.069587	0.19041	0.286979	1.454333	1.065271	0.707594
gene-MA16_Dca002744	11.549131	12.889183	9.244763	27.167553	21.380013	25.848949	10.883586	9.406766	9.932479
gene-MA16_Dca023151	0.837272	1.246063	0.968698	2.897958	2.288024	1.973378	2.123056	2.64456	2.620116
gene-MA16_Dca028456	1.910865	2.009453	1.563267	0.248613	0.760146	0.654626	0.228075	0.525619	0.56046
Dendrobium_catenatum_newGene_7747	0.638412	0.646992	0.953575	1.249997	1.490173	0.767886	2.022786	2.192557	1.222815
gene-MA16_Dca022684	393.312439	420.324341	392.445892	87.040092	84.737679	84.375801	70.302032	66.592064	69.698242
gene-MA16_Dca023488	2.033617	2.782824	1.360411	2.555799	4.148103	3.350659	15.077177	12.410168	13.572362
gene-MA16_Dca011955	2.088032	2.50294	2.169879	3.934515	4.187838	3.414795	7.524563	5.179792	7.266952
gene-MA16_Dca011953	2.038745	1.022404	1.081431	0.871796	0.869383	1.115177	0.522944	0.561985	0.586669
gene-MA16_Dca011959	90.774666	91.879524	91.197945	48.912769	46.190834	48.938602	52.568592	53.488064	52.259468
gene-MA16_Dca014207	2.336659	2.010809	2.115573	2.233529	2.568869	1.611367	4.426001	4.837103	7.139393
gene-MA16_Dca025360	273.892914	264.082672	257.681122	139.993591	136.672104	131.898605	114.372818	104.958801	111.049149
gene-MA16_Dca021621	4.217412	5.329213	4.068122	15.550013	15.243752	17.302832	12.788564	15.366808	10.886891
gene-MA16_Dca019436	23.097063	21.063322	25.215569	27.035814	25.931669	29.071518	58.348862	49.018597	51.123528
gene-MA16_Dca021997	18.606245	20.830717	20.388905	0.409778	0.211125	0.21102	0.081273	0.070948	0.132803
gene-MA16_Dca027662	2.982324	4.49543	3.722197	7.075168	6.968669	5.036271	8.798688	10.416272	7.784365
gene-MA16_Dca018011	147.80423	139.813507	144.459076	11.658725	12.362735	10.771979	27.687532	27.967928	28.209183
gene-MA16_Dca026719	2.43856	2.629457	2.434794	5.978258	5.384723	5.670424	6.933812	7.159992	6.221794
Dendrobium_catenatum_newGene_8361	2.534154	2.639407	3.827987	1.430812	1.508503	1.348804	0.35531	0.600376	0.768314
gene-MA16_Dca011984	7.33549	7.344219	7.416218	3.485153	3.316423	2.131558	0.880407	0.814736	1.447219
gene-MA16_Dca009122	3.24129	3.033562	2.680228	2.954713	2.7104	2.997472	0.596329	0.681409	0.895442
Dendrobium_catenatum_newGene_6638	1.081821	1.049406	1.388152	0.57561	0.559648	0.615575	0.917806	0.664194	1.200904

Dendrobium_catenatum_newGene_1936	2.579542	1.93617	1.802121	2.833384	3.830015	4.07913	5.106298	4.913463	4.395444
Dendrobium_catenatum_newGene_6188	15.872126	17.877371	16.055473	7.629888	7.01565	7.816457	4.252906	4.591409	7.064762
Dendrobium_catenatum_newGene_7580	300.736969	291.36264	286.659302	67.880913	67.92189	18.341427	65.750168	61.330185	60.2258
Dendrobium_catenatum_newGene_3464	65.949929	62.604374	64.325777	16.833746	16.431494	17.14012	17.315239	16.272583	15.421163
gene-MA16_Dca013948	21.94117	23.795055	21.665071	8.754822	8.621155	8.521423	7.923222	7.671729	7.933725
gene-MA16_Dca005627	0.520725	0.748381	0.698331	2.405126	1.931756	1.724332	6.890867	6.574109	6.203476
gene-MA16_Dca008624	202.418823	209.127014	200.217712	60.141129	59.33416	58.495136	57.666534	54.966579	57.854881
gene-MA16_Dca008623	5.796687	5.702898	6.570488	2.230901	2.084094	2.110242	1.828024	1.156815	1.180009
gene-MA16_Dca012306	25.572254	22.650614	23.341679	14.327561	13.048875	12.466759	9.049765	9.624819	10.522511
gene-MA16_Dca012302	573.157532	560.772766	572.368591	950.585449	953.095215	921.114868	1550.2981	1504.73474	1502.67725
gene-MA16_Dca011455	711.307556	736.601318	703.551941	422.095093	400.295135	413.996521	361.928864	349.558136	340.000336
gene-MA16_Dca002536	3.192031	2.456753	3.033264	4.864916	3.465653	3.884258	13.119973	9.306033	10.767741
Dendrobium_catenatum_newGene_12160	26.931299	24.080761	20.213394	13.39449	9.895145	12.404589	11.574392	12.567652	9.00727
Dendrobium_catenatum_newGene_2479	145.331329	155.041718	152.342651	10.314253	9.183331	9.817256	29.201672	27.404644	32.801395
gene-MA16_Dca018622	154.334473	151.937393	152.475235	189.835266	183.819489	196.762527	387.153534	370.014282	371.38266
gene-MA16_Dca018626	9.3127	8.26723	9.380526	22.147003	18.785349	20.380417	20.100384	22.88559	24.061735
gene-MA16_Dca026774	4.897562	6.177621	5.502185	6.74057	6.550463	8.455237	14.139721	14.37779	15.126903
gene-MA16_Dca024739	2.092738	1.03121	2.196007	2.337847	1.145221	1.796923	6.661682	3.824525	5.108338
gene-MA16_Dca021815	645.515991	644.248657	642.820435	411.531799	407.700165	398.861725	276.05838	269.478088	257.497375
gene-MA16_Dca021813	6.356923	6.861872	6.981946	0.186899	0.423557	1.670669	1.111646	0.730535	0.959532
Dendrobium_catenatum_newGene_10566	113.857536	126.023598	114.997734	20.58563	21.28087	19.338078	9.243031	8.743424	7.501375
gene-MA16_Dca022648	31.909706	33.825603	30.318914	14.870378	12.010106	11.91471	16.780262	15.361963	19.241581
gene-MA16_Dca006026	6.620149	5.015553	4.978398	2.942361	4.514937	7.360249	0.59149	1.043443	0.671832
gene-MA16_Dca009192	620.154358	611.86499	607.31665	325.648193	312.380859	325.551971	343.096893	323.826263	322.884583
gene-MA16_Dca007862	1.532382	1.567117	2.758737	3.181251	1.717524	2.808093	5.403075	4.755744	5.193088
gene-MA16_Dca007860	24.326431	25.393646	24.910097	48.304367	48.031643	52.020294	63.330791	61.034016	65.265121
gene-MA16_Dca005702	73.50222	69.82782	66.885223	26.557278	26.867733	27.506105	24.28661	24.140038	24.724026
gene-MA16_Dca004073	1.292935	1.154454	1.52764	0.242818	0.216608	0.388027	0	0.037486	0.116061

gene-MA16_Dca003934	33.518436	35.702217	37.499546	17.7474	17.983917	15.578607	23.634348	22.315416	22.110939
gene-MA16_Dca009341	12.018093	16.202183	13.041666	3.287886	3.031838	2.404238	0.614959	0.718435	1.006227
Dendrobium_catenatum_newGene_6392	19.039562	17.124563	19.462179	8.647544	7.236665	8.197879	19.767385	20.137016	20.434874
gene-MA16_Dca006348	53.554287	50.664677	52.228256	21.531759	16.917591	19.742945	75.66584	72.374947	72.455597
gene-MA16_Dca024284	29.522978	25.682539	23.236645	13.822189	11.000088	12.936798	10.629784	11.56109	9.568851
gene-MA16_Dca014244	1.028713	0.973936	1.472434	0.391219	0.28972	0.360612	1.070766	0.532724	0.472388
gene-MA16_Dca014980	24.483463	28.813236	26.132532	13.693788	13.47109	13.919098	35.358822	34.851086	33.933872
gene-MA16_Dca014982	48.432102	35.554413	31.360542	48.122948	52.198307	44.304886	117.279625	105.675835	110.482697
gene-MA16_Dca013215	33.579208	30.799335	34.28009	3.908242	2.37856	3.596642	4.577882	4.703898	4.811649
gene-MA16_Dca013216	34.902103	34.618618	37.293453	14.100276	14.262261	16.748697	12.44403	13.282576	12.833317
gene-MA16_Dca010602	430.919952	417.45282	414.606415	113.501823	115.468689	104.370003	115.506691	118.500626	115.42746
gene-MA16_Dca010607	10.399559	10.329254	7.903746	5.662681	4.04498	4.697549	6.756128	7.477108	4.58947
Dendrobium_catenatum_newGene_10394	0.214078	0.336464	0.393623	0.504454	0.427954	0.731324	1.223624	1.538322	1.613299
Dendrobium_catenatum_newGene_10398	1.172668	0.792214	0.746662	1.633274	1.995146	2.593933	1.93564	0.935918	1.60922
gene-MA16_Dca000582	0.670211	1.0237	0.992385	1.372098	1.328067	1.913449	4.37165	3.315139	3.32102
gene-MA16_Dca000267	21.552406	16.792475	17.217682	6.39769	8.590702	8.614717	8.875719	10.603902	10.431787
gene-MA16_Dca003056	0.838066	1.207464	1.622568	0.785481	0.76658	0.872411	0.409546	0.562788	0.691517
Dendrobium_catenatum_newGene_13159	5.108814	6.024313	7.005569	0.155101	1.779635	0.142101	6.060104	7.141027	0
gene-MA16_Dca013641	126.394661	118.683578	120.397774	248.833206	246.015121	246.898651	330.771667	303.418365	303.269073
gene-MA16_Dca013645	59.316162	61.981167	60.842724	30.328817	26.777807	24.854183	36.73098	36.755623	31.634832
gene-MA16_Dca014519	1.857621	1.270155	1.327733	2.105949	1.634716	2.754112	3.411828	3.740291	3.459404
gene-MA16_Dca018817	185.341522	194.849915	178.986679	335.904633	322.320007	321.199219	514.338928	511.487366	503.065948
Dendrobium_catenatum_newGene_8734	26.812956	27.607008	24.072351	34.335453	34.304832	34.857128	63.87949	60.705677	66.219925
gene-MA16_Dca021136	5.601493	5.869864	5.953862	3.573022	4.277436	4.112823	3.18487	2.439168	2.739513
gene-MA16_Dca015393	31.711153	30.074413	26.689379	6.283834	7.240987	7.696815	8.068551	6.361248	6.209208
gene-MA16_Dca007235	30.620869	27.706123	26.529921	17.759512	17.390644	16.355118	13.097935	12.376337	13.03864
gene-MA16_Dca007588	62.80901	59.797615	62.284668	32.557552	31.367456	30.964842	30.137402	27.66972	28.992813
gene-MA16_Dca027206	64.395538	63.273598	64.641182	64.775604	70.052628	73.953316	148.520325	144.020981	140.504456

gene-MA16_Dca022321	10.354053	9.644069	11.426551	4.256224	2.724988	2.875097	4.342189	5.291634	4.33254
gene-MA16_Dca024469	12.794717	12.150818	12.199294	3.047326	3.63388	3.362124	3.11081	3.628926	2.75069
gene-MA16_Dca005513	6.377367	6.377447	6.638486	3.205706	2.780651	2.727373	2.746433	2.976559	2.658772
Dendrobium_catenatum_newGene_5741	1.295628	1.172744	0.746724	3.395459	2.531312	3.596314	3.038861	3.062868	2.983765
gene-MA16_Dca003214	71.752411	74.77182	69.233261	81.242195	80.798164	87.22393	39.001583	37.128494	37.612114
Dendrobium_catenatum_newGene_11157	108.717218	109.296101	108.242673	47.694125	47.805532	46.332661	40.02766	38.822829	39.073547
Dendrobium_catenatum_newGene_2826	2.220344	2.394384	3.465088	5.14578	5.968516	8.202389	14.660972	15.80143	14.94
gene-MA16_Dca013875	0.229912	0.291361	0.158413	0.375084	0.629229	0.48972	1.619612	1.549356	1.57852
gene-MA16_Dca015158	2.386207	2.800033	3.410025	3.845657	3.423986	3.741019	0.449998	0.488907	0.86314
gene-MA16_Dca015153	27.590446	30.031364	30.981325	12.337446	12.650166	13.149548	18.79475	20.414282	19.379757
gene-MA16_Dca024090	2.419233	2.207074	2.84876	2.307871	2.246016	2.708722	0.472733	0.543634	0.996473
gene-MA16_Dca015848	13.207729	11.256203	13.792003	6.308993	4.411462	3.413503	5.817983	6.797646	5.038177
gene-MA16_Dca020576	21.637316	22.098997	21.522007	11.415795	10.989046	11.589926	15.9136	15.326522	15.426612
gene-MA16_Dca020574	0.198542	0.291885	0.696741	2.047941	1.566338	2.825479	2.407562	1.020352	1.089465
gene-MA16_Dca026936	15.063456	15.976259	15.684431	37.841122	42.363033	33.86076	23.543119	26.594532	26.196608
Dendrobium_catenatum_newGene_16292	46.77191	48.708712	44.343583	23.505904	24.312394	25.650335	39.617802	35.462245	37.907535
gene-MA16_Dca014442	1.772837	2.495231	2.089	0.643027	0.847845	0.709915	1.893537	1.599848	1.879401
gene-MA16_Dca014496	8.004957	6.749959	8.112105	13.654195	15.875616	12.74021	19.396389	19.901999	15.741837
gene-MA16_Dca014729	6.633461	3.428249	3.911581	4.630006	6.066604	5.485493	9.147059	13.10116	12.819009
Dendrobium_catenatum_newGene_1621	1.397202	1.286099	1.138984	0.592932	0.879887	1.050876	0.514064	0.746801	0.676011
gene-MA16_Dca004957	0.974319	0.806788	0.407095	5.419459	5.137734	5.450398	5.822731	5.542256	5.500376
gene-MA16_Dca004956	46.115913	51.59676	49.28931	18.654039	17.596811	21.105198	14.484521	10.299872	11.737502
gene-MA16_Dca004955	9.148336	9.044814	7.474112	12.69071	14.336623	13.230981	18.481634	18.20162	18.000519
gene-MA16_Dca004951	47.864395	38.827564	43.189705	20.322832	20.294441	20.708054	27.341208	24.562351	27.162701
gene-MA16_Dca007792	12.415411	12.147816	11.922123	2.482223	1.826396	1.606758	2.466398	2.353357	2.819735
gene-MA16_Dca007793	51.790829	54.555611	57.011429	5.541516	7.594676	7.384358	7.112287	5.915961	7.60202
gene-MA16_Dca001795	1.615575	1.633983	2.263651	3.594786	3.996551	5.543554	2.103594	2.500157	2.692694
gene-MA16_Dca006449	10.291533	8.194945	8.999757	3.389476	2.437373	2.140312	9.850331	8.849324	11.093895

gene-MA16_Dca006440	2.609953	1.997252	2.664503	1.274046	0.905984	1.776052	1.333933	0.473019	1.190716
gene-MA16_Dca008519	2.404549	3.181125	2.793351	5.525743	6.559687	5.351229	2.286668	2.378803	2.555972
gene-MA16_Dca004172	11.148459	12.815096	11.175026	3.212267	3.822681	3.752536	3.310902	3.244973	3.356722
gene-MA16_Dca011725	1.533372	1.58468	1.232944	0.239263	0.553961	0.415576	1.587623	1.561039	0.809121
gene-MA16_Dca010927	64.570099	61.511868	60.026562	25.81992	21.322344	23.810553	20.010674	17.887918	17.101067
gene-MA16_Dca012401	1.651864	2.59691	1.651348	0.783495	0.891884	0.982376	0.894865	0.476325	0.819573
gene-MA16_Dca026267	15.225989	15.328601	11.483893	11.514664	16.576611	11.0994	5.617057	4.159898	3.896918
gene-MA16_Dca009587	70.800613	64.48172	61.106533	98.276657	95.950722	90.329605	31.907572	29.28871	28.121138
gene-MA16_Dca009585	223.047165	224.535828	227.585922	152.10054	150.796249	149.031128	108.966652	94.816803	97.634087
gene-MA16_Dca009580	83.422989	77.27372	76.453346	91.218201	91.377281	86.332642	282.890167	239.443787	252.756119
Dendrobium_catenatum_newGene_10907	23.408955	26.724525	26.138638	38.002975	39.047596	39.755486	74.002609	73.041039	72.391464
gene-MA16_Dca025793	0.741154	1.090512	0.819371	1.08112	1.341529	1.035906	2.393169	2.181824	2.120947
gene-MA16_Dca025245	27.110044	25.577469	31.195168	18.236658	22.006037	21.141689	10.693835	9.946446	13.687366
Dendrobium_catenatum_newGene_4253	0.424502	0.160418	0.165193	0.218681	0.5575	0.344929	1.594785	0.878118	1.732468
gene-MA16_Dca010118	168.826431	172.361633	171.832031	56.678726	56.442898	56.796246	52.196541	48.216263	46.321667
gene-MA16_Dca010119	0.299186	0.386392	0.560163	0.118184	0.11077	0.083235	0.071488	0.078524	0.08043
gene-MA16_Dca010112	3.730689	2.798279	3.611309	0.540059	0.682728	0.717586	0.547897	0.609701	0.239075
gene-MA16_Dca010117	3.532986	3.007432	2.834657	7.683614	5.461024	6.071939	8.601687	11.708182	8.897419
gene-MA16_Dca022301	1.792386	3.125167	2.941363	3.416073	4.52347	2.392775	6.478622	6.114069	5.945688
gene-MA16_Dca022977	254.461945	241.451019	267.336212	384.014862	381.84256	387.824524	591.73999	590.746582	548.893311
gene-MA16_Dca018300	7.497072	8.818688	7.92597	4.471882	5.179105	5.190969	3.742573	3.50295	3.446404
gene-MA16_Dca014761	15.734187	16.390779	17.022951	27.456467	29.483019	26.866236	36.640541	34.73798	35.800518
Dendrobium_catenatum_newGene_7335	1.571614	1.25467	1.589119	2.37262	3.174155	3.135077	3.024867	3.36063	3.753546
gene-MA16_Dca001614	2.882364	3.100791	3.987092	2.157254	1.292038	1.574641	13.108078	11.005157	10.366121
gene-MA16_Dca001612	0.66049	0.196478	0.20701	0.330833	0.143366	0.619409	1.710361	1.195546	1.516969
gene-MA16_Dca000052	1.964858	1.457113	1.101062	2.659902	2.556765	3.505738	4.106351	3.589736	3.490133
gene-MA16_Dca010350	3.248139	3.472522	2.386241	0.79877	1.195599	1.192752	3.872671	3.171486	2.589067
gene-MA16_Dca010351	7.314487	7.648489	6.047525	1.584953	1.938407	2.137162	2.680167	1.814345	1.290118

gene-MA16_Dca008704	315.519287	302.447571	298.255066	289.191101	294.698639	298.309631	141.431641	130.745911	141.67923
gene-MA16_Dca016680	1.03885	0.907246	1.305406	0	0.402033	0.071624	0.189778	0.220816	0.035271
gene-MA16_Dca012193	22.957098	27.22584	23.726822	1.367217	1.380785	3.772325	3.090768	3.925448	1.526936
gene-MA16_Dca011555	2.437387	3.273762	2.264675	4.285773	3.191381	2.6766	10.463107	7.739575	7.160391
gene-MA16_Dca016021	8.979134	7.754864	9.117213	7.279977	5.07239	5.300431	0.448927	0.694351	0.714149
gene-MA16_Dca009429	5.591248	6.558516	6.684336	9.283718	6.521733	9.413169	16.101112	11.682549	13.507255
gene-MA16_Dca009423	0.201715	0.064499	0.184254	0.281947	0.457151	0.212208	0.515036	0.544073	0.675729
gene-MA16_Dca002368	28.325499	28.707535	29.001007	18.44771	15.077442	17.934227	13.227122	13.666165	13.064208
gene-MA16_Dca023571	5.096502	4.520227	4.944651	4.245651	6.297256	4.245254	18.684002	17.726965	18.289015
gene-MA16_Dca018549	50.996235	49.611328	51.04044	2.764514	2.158796	2.16397	3.922154	4.254834	4.417126
gene-MA16_Dca018548	25.49976	30.187006	27.276529	9.925529	10.900779	9.518586	8.664907	9.476713	10.9675
gene-MA16_Dca018542	10.123893	9.651554	9.903963	23.264084	21.625366	18.015743	22.236565	20.061132	20.330858
gene-MA16_Dca017641	43.122608	46.536091	47.539143	16.141394	17.214872	17.938795	21.909948	18.635241	22.294136
gene-MA16_Dca026430	43.586823	39.481586	40.479618	0.34273	0.863083	0.53239	0.523646	0.644365	1.244232
gene-MA16_Dca018897	270.03009	255.245438	271.501068	17.631683	16.622925	15.604789	7.434265	5.73857	5.335505
gene-MA16_Dca018899	33.680935	35.782524	34.720901	18.146851	18.172394	18.395512	14.945687	14.364445	14.831645
gene-MA16_Dca020483	1.436873	1.780452	1.724932	0.281493	0.343915	0.370838	0.256779	0.553701	0.62124
gene-MA16_Dca020484	167.007706	165.376938	157.56337	10.670512	9.952299	11.683979	10.274745	10.020905	10.694967
gene-MA16_Dca015040	3.849895	3.763231	2.518025	2.471981	2.70557	2.358423	0.587401	1.227346	0.191624
gene-MA16_Dca024232	341.747253	334.56601	325.83429	72.534882	69.076965	69.629631	39.573811	39.781708	41.362408
Dendrobium_catenatum_newGene_8810	1.405703	1.3487	0.479724	1.399137	1.587766	2.330134	2.875037	2.42306	3.341976
gene-MA16_Dca027282	32.98254	27.363367	28.982224	11.046693	8.830488	7.338985	9.870838	11.322585	8.7571
gene-MA16_Dca027285	78.117706	78.575851	76.855293	29.952036	27.75765	31.940781	77.392517	79.879303	76.647285
gene-MA16_Dca001457	37.575718	39.869297	39.384945	100.851196	105.30925	100.757751	143.470398	150.654358	146.41394
gene-MA16_Dca004244	465.576294	413.223511	437.688538	421.739502	403.639038	352.74942	1166.76025	1051.46216	1036.9281
gene-MA16_Dca004869	1.586146	0.888704	1.304454	1.086787	1.110648	0.564642	5.990355	4.411696	5.683155
gene-MA16_Dca023397	0.439683	0.164359	0.438247	0.700095	0.534342	0.719696	0.80587	0.760679	0.962182
gene-MA16_Dca023394	7.210632	5.605289	6.517032	2.730847	2.274267	2.263879	3.172309	3.41566	2.676164

gene-MA16_Dca011343	39.917061	42.081417	39.516418	8.456025	7.641078	8.838844	13.243956	12.209416	11.580666
Dendrobium_catenatum_newGene_2532	0.788351	0.436802	0.468807	1.684926	1.612698	2.530078	0.761016	1.619519	1.634904
gene-MA16_Dca012839	2.353473	3.078983	2.546599	0.879906	0.755036	0.792013	0.680426	0.651478	0.671558
gene-MA16_Dca019926	45.430954	40.799488	40.329025	17.330709	14.902767	13.278433	16.282839	17.138792	16.212919
gene-MA16_Dca019928	0.547545	0.674062	0.786173	1.429095	1.283431	2.035177	1.291474	1.039282	1.321478
gene-MA16_Dca014866	202.128494	197.842316	195.016235	40.253475	32.003288	32.608471	42.345779	40.981186	39.603271
gene-MA16_Dca000157	11.215002	5.226193	8.266962	0.239188	1.300276	1.010846	2.424322	1.515005	1.014055
Dendrobium_catenatum_newGene_5156	639.650763	592.233807	594.023216	585.323478	592.290837	491.095863	1286.58255	1055.96948	1189.10971
gene-MA16_Dca014419	45.893986	45.337334	45.395756	101.69854	97.490852	108.967651	100.011703	106.762146	103.129677
gene-MA16_Dca009212	1.68946	0.829218	1.389342	2.265645	0.995159	2.585859	4.007844	2.562341	3.906015
gene-MA16_Dca009219	150.842621	138.777328	137.587997	47.742119	47.681149	46.813141	34.799847	32.785141	32.673607
gene-MA16_Dca027957	67.270798	59.651211	65.679924	19.788504	42.568817	16.235012	15.984507	24.543156	28.409307
gene-MA16_Dca025554	70.809929	68.452019	75.269165	144.156418	145.430939	148.631454	206.9328	196.95343	202.130783
gene-MA16_Dca017856	13.819618	12.897615	12.85572	21.408833	16.204647	20.338589	44.519653	35.605629	38.828407
gene-MA16_Dca017853	14.250247	18.752266	16.923717	33.95274	37.587543	34.676064	36.090397	34.423595	36.38599
gene-MA16_Dca019487	28.636673	25.983768	31.236759	11.920455	16.587454	15.722898	10.218478	12.460841	17.096664
gene-MA16_Dca019486	29.904442	28.816507	27.939291	15.911448	16.634844	16.414423	13.44194	14.804867	14.199631
gene-MA16_Dca021388	231.960587	233.375336	224.930695	91.327255	84.384087	88.297058	77.88446	72.879822	74.490334
gene-MA16_Dca021921	3.563516	4.068545	4.296785	1.660137	1.897561	1.709717	2.079771	2.16045	2.298989
gene-MA16_Dca021927	31.710844	27.005232	28.764553	4.876283	3.528301	4.064487	1.771302	2.211077	0.341684
gene-MA16_Dca021926	173.250473	176.690826	202.049973	81.447365	87.249275	77.43531	45.95488	57.086391	48.738113
gene-MA16_Dca021924	12.677017	9.638823	13.880523	15.351892	14.410674	16.455475	8.086596	6.456543	5.31585
Dendrobium_catenatum_newGene_14747	2.973744	3.277626	3.070226	2.866697	2.065984	1.960475	7.912516	10.046122	9.321995
gene-MA16_Dca004134	6.156872	5.994611	6.771767	2.440561	1.744095	2.974958	2.048514	1.27771	0.986324
gene-MA16_Dca004136	9.877764	8.779169	6.511894	2.982513	3.255353	4.672527	1.114362	1.896822	1.523171
gene-MA16_Dca005417	1.854989	1.728756	1.598128	1.73462	2.521955	1.568014	0.187338	0.507126	0.385117
gene-MA16_Dca004132	15.563163	16.976637	16.759842	9.036737	7.153271	7.305674	9.675627	9.435245	10.390628
gene-MA16_Dca003150	187.446411	186.005859	182.965088	84.781532	92.556419	89.911995	71.137535	55.96759	66.892303

gene-MA16_Dca028003	1.278517	2.111309	1.840132	1.436514	0.911251	2.000204	3.951172	2.828375	3.790274
gene-MA16_Dca023007	31.039335	38.927147	40.873604	7.862184	6.82202	9.485444	6.044735	10.521625	12.583891
gene-MA16_Dca023004	1.606395	1.772118	2.503532	8.551185	7.16473	8.787567	14.077641	12.942864	16.76664
gene-MA16_Dca013358	9.839701	10.105742	11.470351	16.58378	12.966343	11.719871	40.032829	48.10284	37.1628
gene-MA16_Dca022097	11.454168	11.411636	12.690429	6.767451	6.314665	6.154735	4.94317	4.717624	3.460939
gene-MA16_Dca022757	388.226776	245.066086	303.065491	87.356285	176.28804	92.191704	124.008743	121.13176	146.823715
gene-MA16_Dca022756	585.436218	579.500854	470.423035	138.122604	338.911133	205.074036	253.971237	262.228821	259.0737
Dendrobium_catenatum_newGene_16804	0.912694	0.758151	0.542066	0.894473	0.414136	0.442397	2.003198	1.613076	2.15603
gene-MA16_Dca028827	25.129547	26.283897	24.791422	55.939316	55.965504	54.890038	116.991982	107.504021	103.517632
gene-MA16_Dca024190	2.82179	3.258461	5.298915	5.087886	6.515223	7.149985	15.115287	13.615833	15.07352
gene-MA16_Dca015963	0.739624	1.266522	0.371554	2.397744	2.273942	1.908602	2.654603	2.427974	4.666472
gene-MA16_Dca000344	18.887482	17.445755	20.339247	30.487551	29.887503	32.669273	58.925289	55.93779	53.267773
gene-MA16_Dca011244	4.688002	3.878699	5.457288	6.862064	5.222789	7.312428	2.387479	2.912937	2.636046
gene-MA16_Dca014584	41.730526	42.472076	42.609463	53.158669	52.480267	58.791397	98.378082	88.794899	89.710854
gene-MA16_Dca014580	63.728737	65.703545	61.252659	5.470491	4.699041	5.472252	4.227226	5.057606	4.710575
gene-MA16_Dca016731	1.00476	1.115498	0.554769	1.221013	0.677681	1.490216	2.876893	2.599852	2.451077
gene-MA16_Dca012601	17.459293	14.886724	16.221195	2.296239	2.475785	2.890711	3.374579	3.687201	4.078673
gene-MA16_Dca012602	18.436531	15.245629	15.771593	2.736329	2.550714	3.693712	3.536012	2.72892	4.677526
gene-MA16_Dca018074	30.541794	33.976295	33.593967	5.256289	4.643435	5.473768	7.713049	7.55793	5.559336
Dendrobium_catenatum_newGene_11556	29.289667	31.130804	30.099907	13.482931	12.580584	11.59588	7.818575	13.06646	11.410117
gene-MA16_Dca025450	0.81711	0.744947	0.926457	1.705724	1.714173	1.986831	2.047246	1.972657	2.530329
gene-MA16_Dca019388	2.648188	2.852554	3.256243	0	0	0	0	0	0.15147
gene-MA16_Dca019382	5.049964	4.121892	4.730419	2.499463	1.586856	3.637838	0.314404	0.593436	0.545007
gene-MA16_Dca018383	7.296108	12.384673	10.294924	2.242751	4.980914	2.126412	0.997389	1.932898	1.300978
gene-MA16_Dca025768	0.605492	0.450408	0.348724	1.347631	1.160038	1.563517	1.093556	0.752681	0.631579
Dendrobium_catenatum_newGene_147	1.04983	1.060679	1.055652	2.199751	2.525713	3.053085	2.810191	2.267194	3.478134
Dendrobium_catenatum_newGene_145	1.558475	1.956892	1.614214	2.565013	2.901533	2.676054	1.456101	1.244665	1.231042
gene-MA16_Dca018928	5.70802	3.79071	3.583532	5.855578	4.644027	4.644028	9.901462	8.390902	9.732859

gene-MA16_Dca015550	4.315868	5.056518	5.624441	1.245227	1.020503	0.936184	0.89481	1.613043	0.825997
gene-MA16_Dca015553	24.135046	23.112202	25.604233	65.83728	63.734245	62.210564	139.733673	142.875336	142.804245
gene-MA16_Dca020172	0.712309	0.711692	0.525855	0	0	0	0.677399	1.041985	1.065084
gene-MA16_Dca005279	8.69501	10.736605	9.141397	4.025116	3.512213	3.839048	2.716109	2.234985	2.656689
gene-MA16_Dca001696	21.523178	20.41785	22.438623	31.952639	29.5655	31.504227	51.410721	48.818626	41.855873
gene-MA16_Dca001699	309.317322	300.05304	316.791626	361.174347	349.075806	364.081299	781.794189	763.729858	761.943176
gene-MA16_Dca005188	7.121715	7.629834	8.780935	0.377605	0.27493	0.552458	2.797416	3.126092	2.372027
gene-MA16_Dca008252	1.981466	2.177333	1.469264	2.093974	1.27886	2.000367	5.441443	4.042302	4.146948
gene-MA16_Dca004584	49.586788	55.821526	50.48708	18.50798	16.225592	18.662525	16.336466	13.961883	18.35541
gene-MA16_Dca004580	126.405235	134.003128	134.501251	19.939367	18.541122	20.519762	4.7184	4.803288	4.775741
gene-MA16_Dca001037	203.457886	216.947067	205.543427	142.856705	131.754257	137.367355	68.776749	62.578613	69.016525
gene-MA16_Dca011009	0.929284	0.580655	0.668559	2.440417	1.154091	1.829234	4.70727	5.299236	4.954466
gene-MA16_Dca000632	87.087486	90.079086	87.782814	11.843521	13.469231	13.354083	24.988729	24.839542	25.30039
gene-MA16_Dca000634	1.981121	4.176454	3.464348	4.35833	5.951605	4.199986	11.606476	7.991292	6.514274
gene-MA16_Dca000638	675.447876	656.155273	678.465393	371.106323	384.703949	365.311859	303.459686	289.978699	266.715576
gene-MA16_Dca012724	1.586592	1.68454	2.597845	2.716826	2.431434	2.773586	5.502796	5.817956	5.558508
gene-MA16_Dca002090	4.220893	3.116008	4.49487	10.398801	8.418028	9.467053	13.009362	11.453328	10.57338
Dendrobium_catenatum_newGene_2006	1.189311	1.519394	1.648071	4.252268	5.586129	4.921785	3.022806	4.415146	4.430295
gene-MA16_Dca022117	14.604096	13.128243	17.069065	49.753246	43.833176	48.706497	58.5313	48.969471	50.23653
gene-MA16_Dca022114	5.745144	4.697165	5.680613	5.314311	5.211484	5.387201	18.415787	16.302282	17.212086
gene-MA16_Dca024878	9.731104	9.529923	9.457291	3.929929	4.538486	3.509177	4.733559	5.180452	5.177145
gene-MA16_Dca014371	5.007853	5.190559	4.63837	4.23744	4.942873	5.187289	36.946022	36.557152	41.334858
Dendrobium_catenatum_newGene_4513	4.438997	4.148548	3.691274	2.661772	3.179518	2.294697	0.894717	1.643541	1.710271
gene-MA16_Dca015020	209.225891	204.97998	206.640701	9.500467	12.94115	11.163256	8.324949	8.18365	8.159287
gene-MA16_Dca021755	5.740919	5.205975	4.710906	0.854417	0.58524	0.898279	1.103111	0.775145	1.558785
gene-MA16_Dca021756	0.524296	0.586256	0.401674	1.078842	1.131714	0.702741	2.087121	2.325363	2.574174
gene-MA16_Dca021753	1.298613	0.715528	1.309195	2.642886	2.213669	2.429178	3.397925	2.35754	3.40105
gene-MA16_Dca020409	892.212219	866.41449	876.249695	405.487549	395.816193	389.135193	245.262619	246.477127	256.063812

gene-MA16_Dca020402	0.971503	0.488914	0.188109	0.689922	0	0.640491	1.843398	1.783694	2.092974
gene-MA16_Dca027750	14.75464	17.598938	18.568991	23.335493	28.203438	29.843601	41.719635	43.741337	46.906406
gene-MA16_Dca023983	136.47229	140.263	142.156693	67.040878	66.769295	68.935234	51.936077	46.780128	48.94236
gene-MA16_Dca023322	95.73864	97.166595	105.027084	37.100071	34.378296	37.487614	55.413803	53.038403	45.917755
gene-MA16_Dca025860	36.192165	38.889515	34.791973	0.271997	0.587442	0.742043	0.607238	0.388374	0.275836
gene-MA16_Dca025869	87.30307	86.778992	81.676216	23.996883	18.12381	20.568113	22.202282	22.845684	21.589729
gene-MA16_Dca019170	11.964095	12.217596	13.646401	3.850455	4.502881	4.530344	5.640564	5.368116	6.134031
gene-MA16_Dca019178	8.266148	10.209988	8.104845	1.232164	1.854423	1.638659	1.732004	1.355562	2.208145
gene-MA16_Dca014030	128.075317	161.579163	113.550484	54.012737	77.712929	65.063148	46.842533	35.030388	46.74165
gene-MA16_Dca005846	6.377629	7.386596	7.889584	2.014366	1.280375	1.748967	1.829598	1.326509	1.377011
gene-MA16_Dca005848	55.724335	55.590099	48.868294	15.407818	12.48734	13.952938	13.687418	13.779039	13.815207
gene-MA16_Dca021416	4.323927	4.821951	4.003412	11.262101	9.516635	10.399026	18.607443	15.284555	18.177448
gene-MA16_Dca003878	72.029015	62.926773	57.037369	18.737986	12.656759	18.872128	14.623782	11.270405	12.135666
gene-MA16_Dca003875	5.630464	4.506284	4.384168	0	0	0	0	0.261723	0
gene-MA16_Dca007551	31.246059	31.041052	29.802641	3.660832	2.819899	3.527707	3.071729	2.693661	3.641158
gene-MA16_Dca007556	9.564692	6.404932	9.643289	0.173067	0.435002	0.184742	0.916602	0.456141	1.225077
gene-MA16_Dca007555	41.830063	42.125515	41.386883	4.926569	4.526883	3.883552	6.771199	5.094367	4.553141
gene-MA16_Dca011219	3.650676	2.568871	3.008083	5.575836	5.696237	6.1057	6.71175	6.075306	6.99126
gene-MA16_Dca010250	9.90586	11.183482	8.638814	12.0099	11.373323	10.463508	2.683509	2.454154	3.355333
gene-MA16_Dca004775	19.054525	17.552174	18.177193	2.481009	2.470793	1.509521	1.974054	1.709896	2.13887
gene-MA16_Dca008428	17.987606	16.407721	18.108961	39.972839	40.162319	39.217297	58.181187	62.696457	54.491875
gene-MA16_Dca003467	0.601958	0.731142	1.147904	0.035402	0.041643	0	0.397751	0.760943	1.084846
gene-MA16_Dca016962	219.416718	218.916641	209.78624	31.157238	27.921314	28.203199	18.480034	15.847533	17.691822
gene-MA16_Dca016964	1.70999	1.869019	1.559997	0.587995	0.594112	0.337265	0.60471	0.585584	0.514468
gene-MA16_Dca012098	53.490967	53.875187	54.969097	104.817604	111.741524	116.351273	116.451134	116.361565	122.85997
gene-MA16_Dca003790	6.130068	7.101796	5.25803	2.704946	2.370905	2.516825	1.884999	1.904394	2.410306
gene-MA16_Dca003792	18.98201	22.942303	21.710878	8.821297	8.023097	9.959624	7.190692	8.800436	9.668893
gene-MA16_Dca011613	1.365224	1.490659	2.124739	5.471442	5.927102	4.070706	4.368202	3.539889	3.347268

gene-MA16_Dca011610	14.131591	14.573686	19.253057	0.672195	0	0	0.431222	0	0.372467
gene-MA16_Dca011611	1.960306	2.302388	2.072602	0.972057	1.187387	0.91076	1.644	0.768544	0.756859
gene-MA16_Dca022838	0.105466	0	0.096739	0.118822	0.054201	0.094676	0.858363	0.840917	0.350278
gene-MA16_Dca005510	12.801328	12.711946	13.480817	8.680684	6.972004	8.383879	6.870655	6.066944	6.523464
gene-MA16_Dca009293	135.442688	131.286713	132.82341	45.05904	41.01194	39.825932	54.854961	55.374805	57.0481
gene-MA16_Dca017768	34.3792	31.861113	32.372574	4.521166	5.742768	4.193015	5.764764	3.641287	5.708763
gene-MA16_Dca015720	3.858496	3.895761	3.936473	8.262855	8.813507	8.779738	11.452271	12.746258	11.278517
gene-MA16_Dca014239	195.980896	193.459854	190.437271	278.875793	278.459137	274.306458	477.386505	443.029236	436.0159
gene-MA16_Dca014231	17.338276	16.610903	17.487034	6.257419	4.662043	3.180885	2.635622	3.146558	2.946921
gene-MA16_Dca025334	50.006458	51.145935	47.307819	61.813438	59.461498	62.084934	110.757225	108.141266	107.451744
gene-MA16_Dca018475	13.039593	12.212105	10.671327	18.313194	14.219707	20.566326	39.172333	41.122684	45.425938
gene-MA16_Dca006962	489.766083	481.089203	449.375641	235.327103	229.633438	232.688599	492.920807	453.11972	481.486328
Dendrobium_catenatum_newGene_5297	2.857932	3.466053	3.330146	0.722535	0.478734	0.747901	0.910535	0.711789	1.014883
gene-MA16_Dca027654	32.294388	34.663082	36.58057	7.126677	3.930859	6.701902	6.488811	5.492193	4.893297
gene-MA16_Dca023828	0.244945	0.547092	0.347967	0.92956	0.350174	0.347727	1.830807	1.374022	1.612262
gene-MA16_Dca004981	17.235561	16.903442	15.870067	8.556687	8.83686	8.799945	11.95585	11.234326	10.372682
gene-MA16_Dca028282	119.118477	127.644157	110.361794	18.725288	21.178865	19.327579	7.501738	6.144003	7.860258
gene-MA16_Dca006278	170.82074	180.236359	168.894135	76.276375	76.714996	78.733536	78.353767	73.111542	78.157967
gene-MA16_Dca006274	2.772999	2.214736	2.999413	5.652016	4.951866	4.721761	10.37854	10.32731	9.308878
gene-MA16_Dca006273	11.884568	13.619964	9.375297	17.237211	16.169584	15.113078	31.218157	28.024334	27.659306
gene-MA16_Dca013931	24.608501	24.617147	24.546307	10.633046	8.061169	8.9203	4.904395	5.464571	4.843976
gene-MA16_Dca009940	10.182365	13.207084	10.395306	2.759642	4.363744	4.281564	4.343753	3.527011	3.346062
gene-MA16_Dca012682	34.301254	30.194616	37.862362	68.408653	60.7976	64.193596	94.141953	88.087212	92.502106
gene-MA16_Dca012685	27.97613	27.077375	25.607376	13.160261	15.438581	12.496263	17.481344	18.462805	17.091976
gene-MA16_Dca011427	6.695917	5.682103	3.79172	2.335838	3.554318	4.590003	12.955618	10.399559	9.641954
gene-MA16_Dca013481	31.31325	33.322956	34.306335	43.215691	46.458511	39.828579	23.623804	19.413965	19.10644
gene-MA16_Dca013153	12.278036	10.300286	11.54124	3.532259	6.353972	5.29288	5.914844	8.318798	4.894724
gene-MA16_Dca023775	52.023388	51.182938	51.866463	42.44487	39.101891	44.994301	14.743726	13.681095	16.684877

gene-MA16_Dca023776	24.920359	24.257519	22.450256	21.267757	22.534224	18.497213	8.815861	8.153641	8.958561
gene-MA16_Dca026185	5.71454	4.671242	7.260074	20.437708	11.810018	19.255192	27.052797	28.118143	28.422579
gene-MA16_Dca014687	16.411119	16.432959	16.380121	9.828663	11.203874	10.513697	8.845184	7.056883	9.623595
gene-MA16_Dca022603	0.61896	0.786931	0.945228	0.444301	0.122881	0.311006	0.897773	0.681746	0.443272
gene-MA16_Dca024764	418.08609	399.054535	428.790039	187.101974	198.443436	196.567719	183.128067	179.237534	184.34285
Dendrobium_catenatum_newGene_490	5.662571	6.326342	4.901515	0.343302	0.42462	0.244655	1.72051	0.757273	0.583566
gene-MA16_Dca022930	20.353815	22.094231	18.9258	8.663925	8.606791	7.366069	5.071594	4.981859	4.504272
Dendrobium_catenatum_newGene_9503	15.867708	18.546719	16.304663	3.413522	3.37394	2.995136	2.429935	3.465244	3.76408
gene-MA16_Dca007851	41.60009	41.419449	42.82132	8.296732	6.94954	7.938718	8.257898	9.635299	9.526649
gene-MA16_Dca005756	1.157643	1.207	0.6655	1.013013	1.188374	1.368097	2.881185	3.186545	3.375104
gene-MA16_Dca016534	23.696228	23.066717	22.597628	39.359993	39.208252	36.155651	58.839226	56.966339	54.380219
gene-MA16_Dca003943	2.103411	2.667459	2.332182	0.308187	0.241412	0.399989	0	0.427858	0.11221
gene-MA16_Dca003949	242.493073	244.947571	243.023224	146.80191	131.385803	144.506729	89.25145	92.308876	88.633972
gene-MA16_Dca009396	345.088135	328.011688	338.220245	135.347275	141.632202	138.628433	137.249344	133.986435	123.144951
gene-MA16_Dca009395	77.178185	73.928513	68.349388	20.811209	21.117434	21.808102	19.076015	18.447897	20.411509
gene-MA16_Dca009392	3.645816	3.348429	3.785857	1.706327	0.804296	1.143565	1.922049	1.432038	1.16096
gene-MA16_Dca013011	128.596161	121.829567	122.476974	227.069473	245.274521	243.08992	114.172218	120.800522	111.83268
gene-MA16_Dca012943	730.677734	722.263123	725.909241	1335.08972	1279.0033	1271.67773	2268.5332	2148.98291	2133.01685
gene-MA16_Dca012941	317.679382	309.986389	315.458893	617.693176	595.149963	586.547668	1118.63879	1104.32446	1055.74292
Dendrobium_catenatum_newGene_2993	1.283793	0.976985	1.008806	0.836345	1.18089	1.354383	2.285023	2.136981	3.08868
gene-MA16_Dca001589	0.817501	1.107076	1.373872	2.212445	0.972274	2.376579	5.051588	3.446909	3.621444
gene-MA16_Dca008236	4.851416	3.277613	6.038364	0.840186	1.022696	0.771055	1.482362	2.093372	2.123804
Dendrobium_catenatum_newGene_14438	1.896181	2.122845	2.846293	3.912056	3.582285	2.629316	7.318847	6.853438	6.110881
gene-MA16_Dca010631	175.013275	178.088623	166.862854	93.435051	84.636391	86.072075	312.065735	313.309937	319.42511
gene-MA16_Dca010630	0.38837	0.416044	0.296896	0.692342	1.595927	0.685367	1.844582	1.423642	1.565705
gene-MA16_Dca016670	2.941102	2.394459	3.02827	4.625489	4.644912	4.578987	14.017823	13.829855	13.073166
gene-MA16_Dca016678	1.913033	2.382137	2.391921	0.287384	0.419624	0.858947	0.090011	0.429328	0.12771
gene-MA16_Dca017692	17.288334	16.780453	15.990628	5.773202	6.556962	6.234068	5.197344	3.962875	4.479703

gene-MA16_Dca017694	3488.99341	3432.3523	3546.65332	2054.64795	2103.54663	2047.66821	1227.49963	1279.75024	1198.74829
gene-MA16_Dca017696	1607.68359	1640.47778	1646.85095	1035.43567	1089.88232	1015.68842	699.510193	753.961304	760.730835
gene-MA16_Dca018849	2.371834	3.030972	3.573975	0.788094	1.128402	1.12612	1.195122	1.762617	1.358931
gene-MA16_Dca015699	9.736537	9.213485	7.717664	1.850778	0.950605	0.779763	0.509724	0.281976	0.918068
Dendrobium_catenatum_newGene_8740	4.375113	3.668088	2.259877	3.176646	2.953581	2.894333	1.185509	0.701779	0.723065
gene-MA16_Dca015347	0.945966	1.457309	0.860512	2.044264	1.727037	2.436776	3.572704	4.524814	4.317655
gene-MA16_Dca001956	0.24077	0.436293	0.713037	0.743844	0.659065	0.731442	1.153138	1.04911	1.663909
gene-MA16_Dca000265	51.142693	50.945213	51.701908	19.059227	18.192036	16.354237	21.486761	19.986065	18.572945
gene-MA16_Dca008357	86.925056	81.017143	86.339043	1.815241	1.983442	1.678713	1.99385	1.919806	1.286355
gene-MA16_Dca008356	10.366324	11.598434	10.474154	6.326414	5.091172	5.194472	2.691827	1.747781	2.383942
gene-MA16_Dca000260	4.878693	4.283629	4.180134	0.64526	0.807032	0.459377	1.400267	0.959423	1.31231
gene-MA16_Dca001377	14.073636	14.071088	14.356796	18.210917	20.894199	17.98703	68.805923	62.955605	64.747658
gene-MA16_Dca014401	0.690161	0.255688	0.424048	1.012575	0.73657	0.876824	3.578906	2.476067	3.595315
gene-MA16_Dca023429	15.534016	10.370608	16.149309	1.241074	0.426405	0.966797	0.680991	0.486642	0
gene-MA16_Dca025698	0.817658	0.480072	0.882979	0.567326	0.212046	0.240857	1.034154	1.263682	1.607459
gene-MA16_Dca011125	39.279823	43.299606	40.979858	16.282846	14.16426	16.482718	11.951666	13.174097	12.073559
gene-MA16_Dca000777	144.599869	135.841995	131.391724	49.750801	47.550102	55.987999	64.846535	65.817276	69.156769
gene-MA16_Dca016470	87.743263	80.975471	85.267899	183.885239	183.318253	193.527451	243.068604	234.080658	233.288879
gene-MA16_Dca004292	0.357162	0.436876	0.849005	1.269917	1.018526	1.516161	3.947716	3.737287	2.953896
gene-MA16_Dca004299	2.332575	1.851299	1.28656	6.707846	4.183908	4.508115	4.423982	4.196878	3.261807
gene-MA16_Dca023125	3.584628	3.352308	3.856738	0.358585	0.211718	0.274124	0.053192	0.242534	0.133199
gene-MA16_Dca008831	11.853204	12.676802	12.967891	0	0.502291	0.105506	0.509751	0.718677	1.018541
gene-MA16_Dca025005	27.521198	26.920551	31.787735	11.078571	9.301321	11.303226	17.588459	13.799009	14.98333
gene-MA16_Dca022385	0.402783	0	0.159435	2.545759	1.679617	0.877378	8.751765	7.200827	6.815374
gene-MA16_Dca024061	4.255911	4.830959	5.407838	2.711987	2.201801	2.220796	1.865886	1.871721	3.070634
gene-MA16_Dca024066	64.020996	62.357037	63.382347	27.070221	25.170643	25.768427	56.189098	52.665855	59.539806
gene-MA16_Dca020528	8.879079	7.380456	8.888156	5.955489	4.366923	4.217991	2.515387	2.648157	2.677088
gene-MA16_Dca016516	0.577467	0.873068	0.590772	0.401162	0.639566	0.532215	0.2861	0.393879	0.194946

gene-MA16_Dca016511	50.127132	50.18438	50.774887	28.293112	29.255276	25.284277	25.169384	25.643829	23.720287
gene-MA16_Dca029129	21.333662	21.77981	20.181973	45.823223	41.163765	34.141685	61.860619	52.139923	54.021866
gene-MA16_Dca023246	2.602988	2.590919	1.84185	4.136858	4.69	2.146386	5.240768	4.978143	5.413994
Dendrobium_catenatum_newGene_10605	0.291128	0.446895	0.656717	0.578773	0.728009	1.024139	0.223816	0.256134	0.158014
gene-MA16_Dca026611	13.739098	14.44604	12.469058	4.2294	3.754732	2.674953	1.069408	1.990642	1.880037
gene-MA16_Dca014774	135.146545	138.001709	138.057892	44.672165	41.709671	43.749722	44.820171	47.765057	46.994049
gene-MA16_Dca028858	15.42443	12.690991	13.636704	7.258853	5.584307	6.348711	6.229495	8.229973	5.139679
Dendrobium_catenatum_newGene_1678	0.883632	0.868022	1.215264	0.891012	1.037133	0.949514	0.57822	0.426947	0.407932
gene-MA16_Dca004902	4.729874	4.949944	5.291917	2.918422	2.612373	1.593121	7.781834	9.538604	11.962242
Dendrobium_catenatum_newGene_7530	4.235168	3.737742	3.623606	0.622631	0.501298	0.449798	1.136258	0.973658	1.117273
Dendrobium_catenatum_newGene_7539	67.967697	67.85791	66.779694	118.042892	112.742851	113.596275	193.892685	179.227448	173.005356
gene-MA16_Dca007762	0.152026	0.119224	0.153888	0.591414	0.336746	0.126384	0.933991	0.573589	0.48302
gene-MA16_Dca005692	0.876849	0.788989	0.730906	2.016078	1.718881	2.297369	2.877733	1.958287	3.901377
gene-MA16_Dca005691	15.296094	17.108149	11.542382	0.152356	0	0.069938	0	0.292404	0.047835
gene-MA16_Dca006411	1.415566	1.115448	1.277353	1.379526	1.052387	1.393359	3.974138	3.830385	4.239762
Dendrobium_catenatum_newGene_15710	1.163664	1.896973	1.353753	1.282233	1.344615	1.048996	4.584991	3.17651	3.978228
gene-MA16_Dca016849	29.395458	30.018049	31.120607	17.956488	15.201852	16.014139	14.58286	15.564003	14.259056
gene-MA16_Dca012358	43.44458	43.953907	44.638386	16.174105	17.334547	17.284386	14.804284	16.2728	14.735376
gene-MA16_Dca011715	8.231263	7.023738	7.321729	24.712353	26.476786	28.059401	25.930239	25.939249	25.517265
gene-MA16_Dca016154	3.539206	4.815641	3.169114	2.085479	1.598498	2.12386	1.89626	1.841208	1.372356
gene-MA16_Dca016157	120.889	118.772972	118.432549	67.238281	60.495216	63.388538	61.17939	56.454185	55.467239
gene-MA16_Dca016153	3.726885	4.081433	2.937816	1.299472	1.667125	1.739159	1.161287	1.913037	1.651687
Dendrobium_catenatum_newGene_2138	10.861362	7.949295	10.836549	1.861905	1.985709	2.365454	3.375598	1.966416	2.976324
gene-MA16_Dca025908	4.632973	4.466069	4.890292	0.255158	0.154923	0	0.157982	0.06263	0.082954
gene-MA16_Dca025636	15.387689	15.968266	18.117563	6.415095	8.882003	5.226068	9.837237	11.048999	9.768675
gene-MA16_Dca025907	3.587887	4.316682	3.744859	0.069066	0	0.054037	0	0	0.09493
gene-MA16_Dca012439	0.597973	0.338406	0.567835	0.185527	0.167294	0.078827	0.342523	0.241294	0.458276
gene-MA16_Dca012438	77.047401	81.254509	75.7892	5.978762	5.322371	5.570034	5.260312	4.895706	4.495881

gene-MA16_Dca022027	10.35713	9.570797	8.507335	3.755987	3.989825	3.775755	4.136889	4.184375	4.680912
gene-MA16_Dca013385	2.173542	2.629864	2.79801	0.17931	0.607331	0.455929	1.022805	0.809379	0.888223
gene-MA16_Dca019521	18.349499	18.405703	19.086794	12.739182	11.024796	14.016875	9.110688	8.807193	7.533739
gene-MA16_Dca019524	60.390129	59.515312	56.827511	31.877024	28.141758	29.114813	28.853745	29.357611	28.532393
gene-MA16_Dca025279	5.526198	4.991983	5.133829	2.856279	2.756653	2.678902	1.662078	2.079419	1.910683
gene-MA16_Dca025274	2.413774	1.905664	2.530505	3.904409	3.11366	2.987139	1.134846	1.398556	0.953831
gene-MA16_Dca025270	639.339722	568.458374	618.696594	629.089844	665.756165	589.556824	1878.23608	1688.95056	1661.20032
Dendrobium_catenatum_newGene_10025	3.678065	2.760513	3.137186	4.385514	4.011265	4.060187	7.364214	6.54651	6.869966
Dendrobium_catenatum_newGene_4205	6.39102	7.878616	6.640491	1.999585	2.726405	2.233499	1.68753	2.225151	2.85947
gene-MA16_Dca028624	18.588875	19.256723	20.354866	3.22472	3.525873	2.848295	4.051547	1.702527	1.423696
gene-MA16_Dca028621	21.309505	18.542301	21.353798	9.80876	9.265491	15.781193	8.143978	7.650177	12.206339
Dendrobium_catenatum_newGene_889	4.37747	5.163588	4.279039	7.722637	7.295609	7.510952	10.416493	10.847979	10.675285
Dendrobium_catenatum_newGene_887	5.371793	6.01774	6.680439	0	0.266358	0.046306	0	0.039322	0.168211
gene-MA16_Dca022927	36.262287	36.963184	36.60656	69.534409	67.462906	73.518303	85.40271	82.898239	80.394547
gene-MA16_Dca022924	9.86216	10.856565	9.400135	7.508985	5.389091	6.516451	3.449465	3.192887	3.874563
gene-MA16_Dca008174	52.546871	56.820343	50.490314	18.050611	13.759575	15.062	16.956661	16.915075	16.96277
gene-MA16_Dca017360	2.347382	1.553663	1.500795	4.501175	3.695585	4.667396	4.952361	4.550724	4.132209
gene-MA16_Dca017361	0.322856	0.390143	0.523907	2.360061	2.749966	2.907334	1.251322	1.599172	1.398958
gene-MA16_Dca017367	20.899267	21.093414	19.867449	12.391209	10.896165	8.740148	7.393384	9.392415	7.706702
gene-MA16_Dca018357	46.234318	41.501678	38.455311	42.094063	44.494637	45.025707	14.08424	16.539669	14.028195
gene-MA16_Dca018358	14.702298	13.667823	14.002548	1.053666	2.669381	1.955031	0.398528	0.668834	1.520405
Dendrobium_catenatum_newGene_9637	179.593128	183.796302	181.002387	72.688952	70.491548	73.413496	58.165638	58.519594	61.310426
Dendrobium_catenatum_newGene_9639	0.260349	0.255028	0.213183	0.593045	0	0.31166	2.979868	2.619219	1.966563
Dendrobium_catenatum_newGene_1002	20.286666	21.701212	18.674401	46.472555	41.384738	42.546084	50.210266	49.859797	50.611187
gene-MA16_Dca009318	168.04248	157.59816	173.893219	89.999863	92.401482	88.879166	65.997368	63.796909	64.439926
gene-MA16_Dca008192	1.872798	2.190721	0.980776	3.499774	3.432729	3.532828	3.857763	4.055726	3.547289
gene-MA16_Dca000063	69.786812	77.661232	75.017761	36.114658	33.080761	36.490971	29.235098	26.575771	26.490664
gene-MA16_Dca008730	4.549926	4.017221	3.689789	0.587383	0.807163	0.76076	1.766302	2.574636	1.73566

gene-MA16_Dca012252	0.028809	0.148503	0.262208	0	0.513488	0.201126	0.78043	0.858393	0.893022
gene-MA16_Dca020959	22.727198	20.413992	17.949295	0.623503	0.525393	0.524159	2.301617	2.071191	2.428429
Dendrobium_catenatum_newGene_16184	15.598813	15.751059	16.208878	3.952983	5.418807	4.18391	3.896749	3.386291	3.117063
gene-MA16_Dca002684	283.12674	272.444977	248.307449	81.359299	68.996147	77.450401	43.485443	43.340084	45.969624
gene-MA16_Dca013611	7.076376	8.239636	9.7821	6.55599	5.549066	4.989662	16.138161	11.361125	12.101573
gene-MA16_Dca009454	317.012878	316.02002	308.744202	151.526871	149.554703	153.199661	143.615509	148.384705	146.512253
gene-MA16_Dca018591	59.782341	58.579769	57.437397	7.506227	7.949145	8.818182	8.714964	9.945667	10.817879
gene-MA16_Dca026449	163.560822	183.929047	181.57016	365.302887	370.35965	387.921753	608.89856	556.663269	349.136536
gene-MA16_Dca026448	688.353333	628.605835	649.820557	996.219299	1085.87878	989.651611	1607.51819	1526.19885	1558.31177
gene-MA16_Dca015611	284.67099	284.451752	279.118225	114.561745	104.89341	104.745644	130.435364	121.945457	124.661125
gene-MA16_Dca021143	35.299549	33.150845	33.186729	22.644958	22.133219	23.297184	14.69898	15.713356	15.029957
Dendrobium_catenatum_newGene_9036	4.109172	4.479181	4.939965	4.31373	4.298853	3.992513	1.780443	1.281257	1.877308
gene-MA16_Dca020238	2.664831	2.435559	2.59434	0.471343	1.157466	1.069814	4.453519	5.048659	3.465379
gene-MA16_Dca020235	82.104691	78.433601	79.246567	42.988056	41.668232	41.877644	54.771538	49.469498	51.61964
gene-MA16_Dca020236	3.339237	2.612205	2.335344	1.028407	0.735537	0.961287	1.160118	0.965171	1.502484
gene-MA16_Dca024843	3.250064	1.65396	1.845034	17.89707	20.313354	18.433788	8.057653	7.309334	9.194764
gene-MA16_Dca024842	44.392223	53.449886	52.548153	1019.07575	999.924805	1014.00476	333.132538	315.432465	332.620972
gene-MA16_Dca024841	81.405128	86.515663	85.024796	915.571655	869.449707	924.245728	502.910065	470.079865	486.027191
gene-MA16_Dca010216	1.761424	1.626394	2.476319	0.481086	1.166853	0.69616	0.902888	1.449124	0.980391
gene-MA16_Dca005563	7.278111	8.385379	8.490788	15.842994	10.248302	14.239746	66.814163	66.309639	64.254089
gene-MA16_Dca006910	1.297557	0.783716	0.700596	1.340473	1.329926	1.294696	1.916798	1.939222	2.353184
gene-MA16_Dca003244	57.649509	52.312534	48.933319	7.417403	7.619712	7.56437	5.551361	6.626226	6.343163
gene-MA16_Dca003245	9.768539	9.505861	9.010056	0.808465	0.482729	1.005397	0.544219	0.121319	0.394263
Dendrobium_catenatum_newGene_9772	0.859511	0.623026	0.494961	0.484464	1.445805	0.503401	1.809742	1.677702	1.038838
gene-MA16_Dca002119	9.883376	11.364245	11.016594	2.968286	2.748353	2.259167	4.895849	6.127147	4.518888
gene-MA16_Dca002117	2248.36255	2239.40918	2285.69336	7989.51465	8046.40772	8116.12256	4516.91553	4271.39893	4224.97412
gene-MA16_Dca002116	761.484741	763.520752	751.387085	422.395355	386.553162	394.906677	743.213501	713.3974	714.883301
gene-MA16_Dca002111	28.379333	29.02837	28.276594	18.961044	18.432255	21.182726	13.70248	13.69443	15.057064

Dendrobium_catenatum_newGene_4564	1.204189	1.096784	1.171881	1.338713	1.709002	1.923413	7.172501	6.545973	7.337915
gene-MA16_Dca012867	29.438057	31.373371	30.681154	16.172855	14.900682	16.145092	15.464578	15.5661	15.729072
gene-MA16_Dca024353	4.90718	6.134019	7.305291	2.150239	2.034614	2.986023	1.394021	0.920671	1.708479
gene-MA16_Dca013809	7.55476	7.314016	6.425734	3.173485	2.530926	2.852484	2.770693	3.178694	2.929488
gene-MA16_Dca000184	44.966591	38.549305	44.975212	68.632622	78.133255	69.175064	131.179688	140.63504	118.020172
Dendrobium_catenatum_newGene_10687	9.492716	10.233766	9.865764	15.675495	15.740338	15.775954	21.191443	21.752169	19.365984
gene-MA16_Dca017137	1.387398	1.095802	1.724079	0	0	0	0	0.066268	0.04221
gene-MA16_Dca016266	1.17704	1.757428	1.524795	1.267995	1.044161	0.789455	0.65534	0.556227	0.495709
gene-MA16_Dca002952	12.724582	12.636784	12.798314	3.209986	3.987442	3.443468	4.040347	2.750806	4.45908
gene-MA16_Dca022514	15.602083	16.375956	15.609994	7.991724	8.393332	7.920015	8.245292	9.061617	8.948072
gene-MA16_Dca022511	189.211334	191.462509	187.110733	193.743164	188.00795	189.198456	407.226379	401.025238	405.114685
gene-MA16_Dca027928	61.560814	57.051121	54.534977	29.565968	26.669384	26.588758	115.788765	103.072021	106.86525
gene-MA16_Dca015798	2.007143	1.886576	1.476733	0.560151	0.526299	0.535042	0.773756	0.411709	0.713131
gene-MA16_Dca019230	14.868752	11.088905	12.137717	19.472643	20.58684	22.343258	59.712936	63.4837	55.06308
gene-MA16_Dca015421	54.876495	53.167957	52.314278	25.670797	26.466633	28.179197	23.920851	21.857327	23.575781
gene-MA16_Dca007458	0.33796	0.327137	0.207424	0.877749	0.388216	0.763848	1.117852	0.870936	0.438481
gene-MA16_Dca001859	17.669708	16.644571	15.809982	5.201816	5.906356	7.400585	4.793111	4.162615	4.020805
gene-MA16_Dca005345	5.829899	5.666783	5.683257	1.092399	1.130394	1.121671	0.831404	0.600235	0.8383
gene-MA16_Dca004431	18.526905	22.199081	19.44809	41.709686	42.370193	44.104008	159.273407	135.886978	139.79631
gene-MA16_Dca004432	151.09938	161.297913	155.414658	15.541612	16.156769	13.523287	16.997477	19.360079	17.332382
gene-MA16_Dca008548	1.264775	2.498058	2.224852	6.003753	5.592531	6.212668	6.77121	6.247358	7.849539
gene-MA16_Dca009967	87.616219	89.470284	82.36396	72.481354	70.876633	71.988808	44.165543	42.183945	40.727371
gene-MA16_Dca001149	0.622446	0.670595	0.454385	0.307322	0.227618	0.447434	1.151855	1.281257	0.839864
gene-MA16_Dca001141	40.931728	46.875027	44.082489	18.101482	18.428532	17.832891	12.521786	13.600368	12.063882
gene-MA16_Dca000435	26.681141	27.467165	26.451859	2.366001	1.523732	1.861524	1.770476	1.21153	1.428188
gene-MA16_Dca000437	6.373524	5.614019	6.64344	2.332354	1.471192	1.857084	2.029967	2.214851	2.712952
gene-MA16_Dca004396	7.932098	8.961926	9.129307	1.776154	1.586858	1.808228	2.108654	2.742128	2.849092
gene-MA16_Dca007117	83.328293	80.235283	86.125992	24.84165	24.012371	26.280005	35.155903	35.058132	31.376371

gene-MA16_Dca006093	1.62872	2.163366	1.634514	4.314722	3.86147	2.857189	4.362388	4.844626	3.373321
gene-MA16_Dca022716	216.865036	185.777939	250.064545	75.896706	146.929626	80.725739	66.282806	81.964897	128.748749
gene-MA16_Dca010979	55.989006	52.467148	51.458038	18.776865	18.293854	17.047194	17.25404	18.821201	18.153587
gene-MA16_Dca023008	50.941471	51.178848	49.527702	9.460919	11.285037	12.398569	17.407053	14.907177	16.149088
gene-MA16_Dca009626	7.870553	8.821346	9.758762	15.955495	14.277619	14.702103	20.596443	21.539013	18.536707
gene-MA16_Dca009625	51.189274	39.005535	41.362591	11.120946	9.132043	13.266481	9.888457	6.970408	8.403711
gene-MA16_Dca022758	579.724731	415.965179	469.948486	155.7173	293.077362	150.618805	201.469543	218.148148	238.182114
gene-MA16_Dca026233	0.696936	0.471507	0.614958	1.67728	1.182413	1.484792	4.153913	4.096552	3.450932
gene-MA16_Dca028586	73.004196	70.777679	68.457954	20.715008	20.025692	22.29957	10.86929	10.626613	10.797009
gene-MA16_Dca009088	96.396225	92.633751	90.952065	2.362126	2.121613	1.707542	3.725433	2.881732	4.308283
gene-MA16_Dca013307	25.300512	26.049625	25.947285	13.709448	14.946414	14.649076	12.791975	12.654753	12.965795
gene-MA16_Dca024494	325.769257	313.76239	330.391815	636.940796	609.531616	649.155212	799.764221	805.045654	768.717773
gene-MA16_Dca024147	16.101934	18.573372	20.653774	0.088538	0	0.111342	0	0.137313	0
Dendrobium_catenatum_newGene_4289	128.712555	129.010757	131.285431	63.793678	57.479412	65.369698	61.014061	62.199932	58.039898
gene-MA16_Dca021517	8.381831	7.434552	8.98803	4.112024	3.659143	4.341859	3.780957	2.102126	4.825107
gene-MA16_Dca021512	1.334582	0.973807	0.695299	0.179064	0.143406	0	0.150836	0.230302	0.276887
gene-MA16_Dca021513	43.993347	43.856503	40.812592	7.134118	5.389328	6.391262	8.05174	7.574512	6.319701
gene-MA16_Dca020642	11.778464	12.887647	14.791131	6.610072	7.203482	6.104241	8.630761	7.107518	7.007949
gene-MA16_Dca015913	16.87524	19.231932	18.944441	22.233471	21.17231	21.477812	39.852272	42.151413	42.699764
gene-MA16_Dca011217	2.710239	3.645741	3.204237	0.97204	1.629382	0.921241	7.256414	4.091116	3.360826
gene-MA16_Dca015042	3.275383	4.847236	5.207634	0.401114	0.791478	0.675308	0	0	0
Dendrobium_catenatum_newGene_8768	2.471441	3.661113	4.008429	4.784127	4.814091	4.804102	6.301084	7.322109	8.382092
gene-MA16_Dca009676	26.266685	27.918978	31.114935	14.849768	15.871213	16.962385	13.349491	12.203011	10.537034
gene-MA16_Dca014635	43.151493	41.354935	43.766205	5.03699	6.116547	6.118857	6.875182	8.060565	8.148364
gene-MA16_Dca019009	5.052459	5.724584	5.523653	3.432739	3.278935	2.699317	2.497822	1.876614	2.725202
gene-MA16_Dca019007	509.56842	525.605103	514.548645	185.084259	183.122543	193.699493	224.213577	221.975037	209.433121
gene-MA16_Dca019000	1.45984	1.273926	1.537793	0.198981	0.353797	0.569539	0.532463	0.530869	0.477911
gene-MA16_Dca021257	799.757568	743.144287	723.094727	278.672028	275.413086	270.185486	290.800323	267.279144	289.552917

gene-MA16_Dca007396	1.864964	1.37688	1.720989	1.987057	2.086009	3.04091	4.975827	4.4071	4.468592
gene-MA16_Dca007391	2.313573	2.144801	1.702855	2.524399	1.782888	1.903868	4.5645	4.744792	4.546991
gene-MA16_Dca003556	324.529846	310.062042	300.581451	154.601486	154.62561	159.314545	143.092194	152.769714	137.947418
gene-MA16_Dca013563	78.585068	79.626999	75.26902	14.166872	15.516563	14.300761	15.355875	17.208317	13.475904
gene-MA16_Dca013696	10.505224	9.174061	10.146747	20.493528	20.136368	18.29948	22.523268	24.966112	23.862707
gene-MA16_Dca013697	5.118442	5.483164	4.635119	2.113233	2.253461	1.880378	1.736055	1.363536	1.591976
gene-MA16_Dca002608	47.278603	53.447273	44.601864	36.184502	36.283852	34.724823	20.31192	18.158201	19.226418
Dendrobium_catenatum_newGene_12565	6.838853	6.270065	6.516284	8.265536	8.001523	6.513546	2.893314	3.979415	2.444096
gene-MA16_Dca023635	20.558739	18.16312	24.831902	14.145339	12.268302	11.695053	8.129741	7.405513	8.10978
gene-MA16_Dca016052	1.19556	1.594671	1.49146	0.365044	0.197662	0.438607	0.816879	0.629455	0.993469
gene-MA16_Dca016055	14.271811	13.77172	17.385717	7.503124	6.06938	6.948545	4.68551	5.438092	4.517984
Dendrobium_catenatum_newGene_5600	18.033207	16.174629	15.766496	34.882137	31.143015	37.680202	48.00589	42.151333	39.8848
gene-MA16_Dca026379	3.717513	4.456403	4.341981	3.70254	3.970707	4.937324	1.999035	2.124378	1.770013
gene-MA16_Dca018514	14.591569	15.172183	16.386606	15.298521	13.80122	16.108414	115.034271	112.579796	114.35833
gene-MA16_Dca018511	1085.3075	1080.4978	1073.87817	1409.89172	1339.0083	1394.18982	439.071381	406.997009	412.175079
gene-MA16_Dca024845	15.569251	14.79094	16.078329	346.678864	332.304626	345.18811	669.190247	666.895386	647.01123
gene-MA16_Dca024844	5.695525	6.237831	4.267872	82.638641	88.406433	87.955048	37.319447	32.935696	34.2248
Dendrobium_catenatum_newGene_2927	2.198256	1.785608	1.95841	5.526087	6.079726	3.57045	6.217212	5.88427	7.167745
gene-MA16_Dca024840	3.811192	3.915941	3.308029	12.138209	9.13656	12.21249	10.234471	11.757822	10.820519
Dendrobium_catenatum_newGene_2929	0.296411	0.467117	0.347228	0.999979	0.477834	0.549783	1.353667	1.105314	0.841469
Dendrobium_catenatum_newGene_4565	8.831493	8.658171	9.181239	6.787578	7.813738	7.634205	19.162493	15.699656	15.941949
Dendrobium_catenatum_newGene_3789	0.277757	0.477224	0.425861	0.517068	0.850378	1.142481	0.743823	1.013493	1.097208
gene-MA16_Dca027762	40.867386	45.116722	39.855824	22.094994	25.747818	21.039978	19.163076	19.003691	15.519191
gene-MA16_Dca009576	1.826924	1.747679	1.207554	0.751331	0.296059	0.67575	0.754012	0.620094	0.758861
gene-MA16_Dca025854	69.729179	63.910931	60.068237	70.337616	65.745193	69.429977	186.133133	175.440704	180.954727
Dendrobium_catenatum_newGene_16542	24.013045	25.447185	26.498519	13.132363	10.709232	13.307256	10.533019	10.662296	10.624604
gene-MA16_Dca018250	2.928482	3.432815	3.529538	2.963489	2.415319	2.835265	1.428556	1.820108	1.581199
gene-MA16_Dca006997	202.385178	193.152573	215.199448	115.376968	109.098434	133.219101	111.160095	103.00663	99.090996

gene-MA16_Dca012497	15.879473	14.716883	17.936291	4.731067	4.532359	4.259459	6.412137	5.349978	4.054964
Dendrobium_catenatum_newGene_1294	6.728026	7.772243	5.260551	1.493734	2.513543	1.571069	1.916259	2.905178	1.884997
gene-MA16_Dca003826	39.519474	40.652225	40.288563	17.99971	18.017395	19.608938	21.196064	22.337046	21.123274
gene-MA16_Dca003827	0	0	0.044955	0.209984	1.980718	0.98083	1.367558	1.780091	2.514826
gene-MA16_Dca003824	76.560814	72.669037	77.356819	130.797165	121.791473	125.308571	158.102905	166.982254	167.360916
gene-MA16_Dca003829	0.613726	0.638708	0.561556	14.734691	14.012338	12.859087	30.788788	27.07048	24.570436
gene-MA16_Dca002883	9.642774	8.607979	8.49446	5.484344	2.847282	3.29313	5.487177	5.691369	6.624057
gene-MA16_Dca000105	27.516665	31.58021	31.835222	10.627368	10.433489	10.725863	9.031592	9.979411	9.151418
gene-MA16_Dca000100	0.579427	0.573191	0.463699	0.813906	0.760441	1.091256	2.336617	1.767635	2.406955
gene-MA16_Dca010281	53.206398	54.721203	51.136059	13.367549	13.225926	12.921165	12.695291	12.50738	12.934619
gene-MA16_Dca016938	4.499321	4.543969	3.931313	0.303167	0.670338	0.391733	0.689766	0.826827	1.488235
gene-MA16_Dca011622	62.905033	60.45507	61.107216	3.292848	4.07841	3.207871	1.890469	0.488661	0.496036
gene-MA16_Dca000816	1.143163	1.623115	0.512417	2.609667	1.48219	2.839511	4.806463	3.335221	3.534017
gene-MA16_Dca022593	8.581537	8.099628	7.764798	1.093735	0.648373	0.523422	2.578382	2.9724	2.89416
gene-MA16_Dca022599	6.53464	7.059275	7.474987	1.946692	1.469669	1.968	4.566575	4.049447	4.136096
Dendrobium_catenatum_newGene_12068	1.047974	1.533668	1.386658	0.4195	0.334484	0.388	0.225932	0.378992	0.154864
gene-MA16_Dca022338	27.790667	25.854513	26.435928	10.858837	9.463351	10.601808	12.276508	11.59017	10.780439
gene-MA16_Dca026650	22.133236	21.1814	21.538052	11.404962	8.393158	12.543717	7.104167	4.53934	3.57227
gene-MA16_Dca026652	11.995856	12.26125	13.710601	10.491584	7.70163	10.000286	3.992433	3.878685	5.547757
gene-MA16_Dca024653	237.493454	228.649368	241.419464	542.938843	577.43988	540.576843	450.699951	431.752472	406.90271
gene-MA16_Dca025302	6.200138	8.994379	9.162866	7.728559	7.218477	6.558284	2.207942	2.505981	1.162216
Dendrobium_catenatum_newGene_13825	2.233135	2.385628	1.821452	1.413598	1.500741	2.90543	5.752935	5.172676	6.073723
gene-MA16_Dca020087	175.68042	180.304016	174.448715	97.737656	86.201355	98.945122	93.380959	89.399323	91.708275
gene-MA16_Dca016577	18.355797	19.390148	20.711863	5.48349	7.821399	6.524592	7.896045	5.626658	6.738477
Dendrobium_catenatum_newGene_11301	6.348112	5.583468	5.945756	4.816567	3.635949	4.070943	2.922519	3.248134	2.74018
gene-MA16_Dca006766	3.816727	5.678317	3.160781	2.446199	2.030532	1.558217	0.868122	1.376513	1.798672
Dendrobium_catenatum_newGene_6252	1.791716	1.617298	2.255749	1.258778	1.244969	1.22624	0.75424	0.586505	1.101099
gene-MA16_Dca001233	6.17615	6.098877	7.087122	12.837944	13.324347	12.602096	20.155054	19.659151	19.113047

gene-MA16_Dca005447	1.816671	1.876382	1.217305	2.799455	2.559208	2.013505	1.319309	0.508994	0.796197
gene-MA16_Dca023293	29.680107	37.152477	32.941963	3.178392	3.369112	2.530227	3.70372	4.323377	1.824064
Dendrobium_catenatum_newGene_1825	2.013352	2.764958	2.03583	1.449888	1.401269	1.583495	0.82293	1.10424	1.132967
Dendrobium_catenatum_newGene_5341	1.206285	0.543322	0.771617	0.663278	0.547607	0.597421	0.356126	0.117867	0.366135
gene-MA16_Dca009119	0.560381	0.286579	0.468741	1.810246	1.966963	1.836096	2.654477	1.822141	2.492163
gene-MA16_Dca004317	0.770203	1.129984	0.584892	1.006149	0.688352	1.44748	2.205545	2.200075	2.467314
gene-MA16_Dca004316	18.693951	15.618731	21.261517	1.245287	1.433724	0.96401	1.271193	1.188203	1.28028
gene-MA16_Dca004310	25.778862	20.654041	19.555073	5.194879	9.235003	4.915403	3.960869	2.811797	2.539364
gene-MA16_Dca007156	5.720437	5.610405	5.646885	8.711428	7.541656	9.452868	14.518107	12.493814	13.144242
gene-MA16_Dca007158	1.566452	1.023474	0.893923	3.211398	3.363997	2.247025	3.421408	4.771733	3.713201
Dendrobium_catenatum_newGene_2753	0.43902	0.367163	0.539074	1.784274	0.629851	1.514975	2.773233	3.016429	2.332418
gene-MA16_Dca006200	3.471521	5.214526	4.316084	13.8037	12.239185	12.605894	13.844482	14.063803	14.808652
gene-MA16_Dca003665	57.894608	57.490623	55.788956	24.883526	25.170572	21.978453	46.484921	44.653786	44.215973
gene-MA16_Dca024412	7.629958	9.976424	9.247668	3.145154	3.82037	5.017191	5.217996	7.229908	5.924466
gene-MA16_Dca015992	18.107443	15.738199	18.905001	7.778274	8.487083	6.560578	10.411462	9.193747	10.430555
gene-MA16_Dca016746	0.161113	0.342096	1.004736	2.508163	2.870961	2.833326	4.117967	3.938691	4.565256
gene-MA16_Dca027809	7.101899	4.647366	3.343292	1.759872	2.504289	1.294596	0.497024	1.134447	1.894847
gene-MA16_Dca017908	5.19626	4.938547	6.3793	27.849781	26.876701	25.416641	29.079979	29.918377	29.524368
gene-MA16_Dca018689	24.866535	28.079018	23.344753	7.250091	8.251784	5.379663	7.134162	7.387622	6.546313
gene-MA16_Dca018685	2.515038	2.785519	1.299906	0	0	0	0.131466	0	0
Dendrobium_catenatum_newGene_8456	0.039466	0.967399	0.434786	0.107514	0	0	0.846728	0.902824	0.858344
gene-MA16_Dca006010	7.185965	8.43198	7.948497	3.505224	2.981915	2.616724	2.298944	2.156474	1.84738
gene-MA16_Dca021214	51.688934	48.052834	49.4506	0.715632	1.199944	1.765598	0.70939	1.009675	1.311932
gene-MA16_Dca005222	190.617783	188.357162	184.796127	122.928078	116.606865	122.564529	85.785965	87.790474	85.394417
gene-MA16_Dca005220	2.741686	2.990683	2.792763	3.005922	2.287432	2.499379	0.791372	0.771888	0.312579
gene-MA16_Dca004576	100.738937	99.258636	100.569199	173.33606	177.097626	164.197433	231.228668	230.268143	219.487885
gene-MA16_Dca004571	7.874555	6.969589	7.190839	4.768365	4.16749	3.274766	3.146671	2.721324	4.295318
gene-MA16_Dca004572	72.139595	70.792221	75.144058	40.27063	42.418652	38.23283	37.05275	32.836308	30.361582

gene-MA16_Dca008223	17.695827	16.557188	16.200962	9.633821	9.50715	8.878436	8.860125	8.619113	8.809106
gene-MA16_Dca008222	318.758148	314.947662	317.585144	152.903046	151.898132	155.47525	114.053085	115.347618	120.793449
gene-MA16_Dca007882	1.836611	1.751959	1.949796	5.234709	4.855471	5.597533	1.919192	1.848455	2.272771
gene-MA16_Dca005768	6.18483	7.269962	5.729276	10.989459	9.507553	9.52028	16.241068	15.948064	14.360788
gene-MA16_Dca013713	5.33192	5.195023	4.907655	3.273017	2.80457	3.321545	2.376725	2.385684	2.45218
gene-MA16_Dca013712	164.403442	153.71196	155.881699	100.006561	91.518509	97.411049	442.706177	412.789825	409.453094
gene-MA16_Dca013716	137.428116	139.423233	134.7995	101.995544	100.988457	101.583649	57.9473	58.346752	60.842384
gene-MA16_Dca016508	0.649422	0.380185	0.736214	0.110672	0.152138	0.135381	1.910869	0.933398	1.448165
gene-MA16_Dca010831	424.054596	409.738983	413.686584	252.613846	241.846085	240.172256	121.951172	115.69075	112.021286
gene-MA16_Dca003338	4.648052	5.245437	4.785493	2.898873	2.639413	2.089743	2.575696	2.554744	2.409849
Dendrobium_catenatum_newGene_7683	1.722134	1.956934	1.972831	1.78996	1.673737	1.832544	0.993584	0.916313	0.578857
gene-MA16_Dca007053	1.789013	1.496609	1.971195	0.242593	0.293446	0.204305	0.404119	0.123233	0.275297
gene-MA16_Dca025643	2.317616	1.052367	3.480679	6.789807	7.580373	8.068462	4.599611	4.50121	2.705743
gene-MA16_Dca005963	3.260448	2.389929	2.010797	4.89612	5.219667	4.601685	6.62396	6.318089	5.876378
Dendrobium_catenatum_newGene_16954	4.067101	4.676755	4.582892	1.592481	1.331619	1.696702	1.715521	1.703389	2.040122
gene-MA16_Dca006085	19.132357	16.236225	16.656496	3.166394	5.134775	3.167912	5.815489	4.868176	5.466569
gene-MA16_Dca002419	30.327108	30.092245	30.815182	19.248068	19.567276	22.747498	48.863964	49.068665	48.059532
gene-MA16_Dca011318	1.718005	2.199162	2.069732	1.487861	1.109667	1.128648	0.541667	0.936712	0.810323
gene-MA16_Dca011317	78.355713	78.230797	67.471855	21.536955	23.651567	19.847675	16.254229	15.569936	18.202993
gene-MA16_Dca025395	9.692465	11.659103	9.975366	17.449045	16.777885	19.948645	21.093004	25.178612	25.430943
gene-MA16_Dca020455	74.562004	79.548012	78.054031	13.175419	15.163401	11.759256	10.98598	12.381409	13.136541
gene-MA16_Dca000522	29.043489	28.54862	31.585443	15.331864	16.574234	18.128302	9.169445	7.020255	8.775
gene-MA16_Dca023375	8.566407	9.49114	8.536954	40.510811	38.979694	41.522396	3.574712	2.876042	2.477712
gene-MA16_Dca023372	41.130989	39.864071	43.450645	22.58774	22.568443	24.30349	20.9795	21.181379	19.371805
gene-MA16_Dca019462	43.103024	44.322128	46.768856	6.068097	7.09779	6.895698	6.574896	6.447721	5.895909
gene-MA16_Dca027003	5.58817	3.8628	4.178169	3.952625	2.842047	2.683222	1.132021	2.145685	1.076949
gene-MA16_Dca001926	36.380661	32.99144	32.845203	99.631073	94.981285	99.080833	242.137634	252.146835	251.521957
gene-MA16_Dca015713	177.766647	189.181854	200.044952	68.810097	64.767647	65.707024	55.91317	48.687401	48.895081

Dendrobium_catenatum_newGene_3857	0.070946	0.035546	0	0.161653	0.226654	0.37171	1.021953	1.713397	0.726508
gene-MA16_Dca011159	0.4345	0.50976	0.858773	1.659643	1.468723	1.375637	1.031773	0.966526	1.337017
Dendrobium_catenatum_newGene_4854	4.29306	2.551607	2.74058	11.396259	13.642297	13.567654	11.131685	13.889419	7.46627
gene-MA16_Dca000720	6.661032	6.50529	6.170032	12.639151	11.542398	12.001639	23.367041	24.006191	22.400175
gene-MA16_Dca000722	7.908812	8.256708	7.50102	3.978365	4.180795	4.769207	1.805268	1.527456	2.154723
gene-MA16_Dca000724	1.337476	1.901069	1.421621	0	0.035812	0	0	0.07563	0.215071
gene-MA16_Dca000725	3.898964	4.923995	3.396557	0	0.037285	0	0.345581	0.322319	0.321637
gene-MA16_Dca002767	11.096375	11.932055	12.872795	7.043096	7.359162	7.644426	5.346391	6.208653	6.466577
gene-MA16_Dca000897	112.989021	105.603645	112.951477	63.152592	63.090942	61.49802	35.730801	37.487579	36.86602
Dendrobium_catenatum_newGene_16445	2.969597	3.308268	3.496108	7.086504	7.333238	7.066489	9.657478	8.94268	7.495222
gene-MA16_Dca023179	0.72713	0.808884	0.74103	2.753087	1.848308	1.771636	1.631819	1.292464	1.009867
gene-MA16_Dca009712	59.772198	56.589893	51.003063	31.750561	33.759079	34.552597	26.169718	26.628099	23.612589
gene-MA16_Dca009716	23.295843	20.932386	23.06498	13.319785	9.803425	12.893341	10.000221	12.236505	9.649907
gene-MA16_Dca009717	9.597166	7.887527	7.623809	9.645672	7.813327	7.667998	18.509029	15.103137	19.076035
gene-MA16_Dca009718	128.841248	132.67334	127.333389	57.429726	53.431316	58.206596	51.839622	52.33873	53.308842
gene-MA16_Dca012544	0.186319	0.277267	0.154534	0.473013	0.444634	0.741552	0.661769	1.231167	0.739746
gene-MA16_Dca028479	16.396997	17.950006	16.520584	14.426555	13.270033	11.931519	5.694024	4.442978	5.491006
gene-MA16_Dca024960	10.743181	10.232554	9.594213	8.637953	9.345558	7.834466	4.587849	4.105734	3.565906
gene-MA16_Dca014886	7.702411	6.217975	5.440611	10.219008	9.742972	8.058379	18.556688	15.2178	16.234554
gene-MA16_Dca019453	11.373598	15.389397	13.992407	1.552913	1.256587	2.332868	2.301995	3.448083	2.923793
gene-MA16_Dca021603	6.644184	6.347311	6.523833	4.199536	5.273847	4.203768	10.829589	8.271492	9.669337
gene-MA16_Dca024035	114.274956	115.05024	115.839722	73.543571	73.613228	71.861115	54.833794	55.302444	53.743992
gene-MA16_Dca018174	102.205238	111.03627	110.810425	43.220413	42.870457	41.013931	51.556564	53.385586	46.177509
gene-MA16_Dca018198	73.386765	73.8078	73.070145	19.267628	18.652367	21.490213	8.575262	7.13276	4.744722
Dendrobium_catenatum_newGene_11257	24.723553	22.383507	24.003122	0.203108	0.455516	0.3309	0.668252	0.266436	0.463821
gene-MA16_Dca014747	342.373596	320.352661	322.39621	5.33469	8.240319	5.989715	4.950488	5.924708	5.788403
gene-MA16_Dca014746	18.60132	22.450647	19.379089	29.516493	31.804119	29.489456	69.137817	61.578175	61.841816
gene-MA16_Dca014748	1.504748	1.588155	1.381323	0.602572	0.827566	1.4818	0.509733	0.661746	0.721557

gene-MA16_Dca019716	118.544136	118.437637	124.990257	93.735359	88.733597	97.12851	204.710526	192.666245	210.220123
gene-MA16_Dca019718	115.763748	107.759491	111.497536	144.989334	140.238785	148.852707	245.643509	237.0905	239.792816
gene-MA16_Dca006284	4.808107	6.070931	5.07235	0.674104	0.29356	0.257321	0.850599	0.46006	0.398993
gene-MA16_Dca001882	1.338828	0.339185	0.538399	0.635823	0.876743	0.799961	1.893691	2.192811	2.639087
gene-MA16_Dca005399	1.133272	1.153597	1.105673	4.142953	4.868181	4.137031	14.559745	12.593588	12.59707
gene-MA16_Dca013968	3.216265	3.537636	3.188574	5.241879	4.395845	5.972567	8.063812	8.88502	9.493219
gene-MA16_Dca004110	14.119277	15.83263	13.358296	25.684311	22.559473	26.476753	30.988153	30.116777	30.20042
gene-MA16_Dca008646	3.265765	2.96693	3.696055	5.457443	5.542625	6.529773	7.700029	7.587582	7.573382
gene-MA16_Dca004118	13.216917	13.139786	11.627232	4.696241	4.274131	4.1122	3.958335	4.874467	5.622451
gene-MA16_Dca012363	5.731395	6.011294	5.120894	12.127723	11.405343	14.316603	14.176091	14.12318	12.32502
gene-MA16_Dca000910	316.907654	322.217621	323.907867	216.023987	215.298859	219.147034	131.11528	121.350533	122.877869
gene-MA16_Dca011477	9.482707	5.972261	6.545438	11.760933	17.285242	13.620538	17.138668	19.528063	13.072637
gene-MA16_Dca012265	36.15377	34.50668	36.14563	22.641848	22.734282	24.973749	17.851889	18.856567	17.636242
gene-MA16_Dca009695	3.595817	2.926666	3.577004	3.445851	2.708438	3.947601	0.408621	0.736599	0.593683
gene-MA16_Dca009693	35.088089	47.085556	47.996277	48.435349	49.476345	48.888172	151.250931	154.893509	151.488434
gene-MA16_Dca002511	13.744544	14.249167	13.864191	20.026932	21.488892	23.931845	32.410439	27.540661	28.172693
gene-MA16_Dca002515	9.294312	7.161649	7.000956	26.319094	34.779484	20.388388	25.433466	26.371691	30.949352
gene-MA16_Dca002514	5.670595	6.321491	6.127721	10.045634	9.744473	8.118229	15.072427	16.879883	15.648076
gene-MA16_Dca010981	39.549313	41.753704	47.420284	22.505033	21.003593	18.93	23.230104	20.354303	20.896919
gene-MA16_Dca010982	40.439304	36.987629	38.690907	14.946389	11.895359	12.792274	11.374117	11.420538	11.147508
gene-MA16_Dca024547	3.295759	3.103278	3.238928	4.570189	4.719499	3.610363	9.380632	6.727753	5.704516
gene-MA16_Dca026284	0.318417	0.173722	0.597314	1.688964	1.264827	0.853869	1.633985	2.099056	2.370842
gene-MA16_Dca022076	6723.18359	6568.07861	5660.91797	2857.44873	3469.53784	2888.05713	3613.1023	2930.06226	3438.25342
gene-MA16_Dca025483	73.535255	66.344177	79.720253	12.9214	13.136728	14.699594	4.816596	6.010002	4.56216
gene-MA16_Dca015589	451.317108	489.8927	462.069946	37.667061	34.940376	35.549641	30.952978	26.071495	20.861385
Dendrobium_catenatum_newGene_14206	25.186317	23.443796	26.547611	11.903019	12.094784	10.639973	9.837658	7.985337	9.044916
gene-MA16_Dca014182	43.664673	44.252266	46.14069	23.258684	17.818731	19.109859	11.769007	13.517145	13.707285
gene-MA16_Dca014184	8.55871	7.701402	7.56281	0.853406	1.536582	0.835754	0.577494	0.921891	0.435868

gene-MA16_Dca014186	7.834657	9.11785	7.133779	1.117534	1.170153	1.452688	1.610107	0.675717	0.518578
gene-MA16_Dca022103	24.556976	20.679708	23.463903	9.015975	8.573655	8.768081	5.933249	8.009872	4.637593
gene-MA16_Dca007806	1.104995	1.10648	1.0415	2.029782	1.776453	1.880507	2.54454	3.462713	3.487123
gene-MA16_Dca007807	10.788198	10.080695	11.510191	17.852556	17.338545	13.153975	32.067402	29.232555	28.111055
gene-MA16_Dca007800	4.57376	4.209191	5.11888	0.856496	0.688375	0.872006	0.154608	0	0
gene-MA16_Dca022916	9.267338	8.674274	9.789879	20.69698	21.38542	21.136765	22.958546	21.134066	19.803528
gene-MA16_Dca011834	0.500178	0.940244	1.091668	2.007613	1.414824	2.243538	3.5398	1.984808	2.235332
gene-MA16_Dca025680	1.147933	2.468044	1.376994	2.374453	2.978878	2.600798	4.170176	4.340706	4.148519
gene-MA16_Dca004053	8.85815	10.061852	9.702868	28.645481	25.97122	29.160641	17.220007	16.626308	18.676367
gene-MA16_Dca018472	19.092642	21.551229	17.369215	25.447943	23.138161	24.467237	74.870506	64.904312	62.575901
gene-MA16_Dca005983	1.89984	1.235529	2.983083	1.609878	2.772394	6.037223	5.701461	3.73846	5.618696
gene-MA16_Dca018477	70.345909	71.054527	73.54657	9.48586	19.430206	15.826563	14.50528	17.157707	11.423857
gene-MA16_Dca018478	13.457854	8.831146	10.869708	4.128812	4.721045	5.028824	4.930035	5.983177	5.000965
gene-MA16_Dca002065	14.040874	15.047563	13.098495	9.206168	7.843569	9.107588	6.576995	5.642195	4.790393
gene-MA16_Dca002067	112.149933	105.240753	112.85276	80.696877	78.737022	80.284744	36.881882	35.003937	34.420109
gene-MA16_Dca009320	79.390335	73.407364	68.62957	23.706491	25.552465	23.788296	31.470707	29.756176	30.999954
gene-MA16_Dca009329	54.816696	63.630859	57.125156	19.857821	22.294912	20.37883	27.606115	31.887699	30.868778
gene-MA16_Dca009328	42.886284	45.237339	46.474567	21.083437	18.715607	18.585445	21.042509	25.507608	21.174664
gene-MA16_Dca006362	17.081631	14.944501	15.875202	9.09953	9.592451	12.499988	6.872751	6.112794	6.434781
gene-MA16_Dca012915	0.807427	1.158795	0.707096	1.666545	2.143285	2.017512	2.448946	3.172591	3.191988
gene-MA16_Dca009816	65.560402	64.726555	62.752815	23.235392	25.51963	25.728416	35.173775	30.767292	36.711681
gene-MA16_Dca009813	45.22987	48.312363	45.061508	24.532017	28.302467	27.464979	18.921036	19.010277	17.077852
gene-MA16_Dca019820	15.059927	17.88842	15.721343	8.295299	9.628039	9.319668	6.277696	6.182237	5.951985
gene-MA16_Dca012288	51.777817	49.724777	55.539581	44.331409	48.973824	43.957996	17.737972	12.530951	11.507022
gene-MA16_Dca012286	17.309765	15.667164	16.215185	9.03927	9.47067	10.170035	7.174795	7.814229	8.203097
gene-MA16_Dca020989	811.311768	1073.2666	959.977478	310.387543	497.075439	429.769592	333.072784	305.90686	402.122162
gene-MA16_Dca011534	1.66798	2.098815	1.637753	0.711223	0.719138	0.344402	0.102021	0.425959	0.512234
gene-MA16_Dca003072	75.487312	77.254723	76.480339	26.52437	35.484451	31.293344	23.248024	19.274986	25.464165

gene-MA16_Dca003076	14.532847	13.555596	13.952194	4.781579	3.17023	4.381829	2.113497	1.374121	2.112756
gene-MA16_Dca003078	2.662411	3.761913	3.713526	1.482413	1.52949	1.787536	1.320105	1.813989	1.23642
gene-MA16_Dca013669	14.794641	16.370977	17.675789	7.724499	6.019035	5.76356	7.043334	5.567472	7.365857
gene-MA16_Dca023511	2.599902	2.239224	2.682353	0.672818	0.497299	1.716315	0.857516	0.328872	0.885309
gene-MA16_Dca014577	17.860374	16.314306	16.607809	10.72386	12.124752	10.834726	6.524855	6.664744	7.128919
Dendrobium_catenatum_newGene_2357	7.777104	6.224229	6.360125	5.815519	3.470321	3.617471	3.561424	3.254655	3.902152
gene-MA16_Dca015626	40.73502	40.661102	39.510796	98.009789	89.126328	89.499916	114.182823	109.007317	108.516968
gene-MA16_Dca021119	482.275879	484.021729	464.794861	199.41687	193.022903	198.522675	179.7668	163.75621	154.768982
gene-MA16_Dca013963	13.552556	17.877882	18.86413	19.775856	21.16301	18.481998	5.666336	4.937591	6.450172
gene-MA16_Dca007215	7.093102	9.055578	9.057634	4.893085	4.973987	4.90058	12.645067	9.198024	12.204338
gene-MA16_Dca008551	2.779206	2.137416	1.597487	4.775159	4.838077	5.895794	8.452604	9.07868	9.578593
gene-MA16_Dca028973	10.133825	8.389377	8.758559	0.968719	2.47665	1.553453	1.008705	1.607214	2.678314
gene-MA16_Dca020798	67.649315	63.303528	62.905769	11.55336	17.719574	14.351281	11.328117	11.660143	9.889068
gene-MA16_Dca020792	8.011602	5.389423	11.421147	3.390807	5.945082	4.586838	1.464324	1.212288	3.292348
gene-MA16_Dca006163	19.716095	20.101803	20.092791	19.710838	16.766426	19.352135	148.328339	143.033417	140.974274
gene-MA16_Dca006166	18.350872	19.490206	21.282589	9.52582	8.962419	5.932352	66.382355	64.673653	58.174183
gene-MA16_Dca008305	17.69281	19.290335	15.560475	16.435291	14.980002	14.006392	31.557055	33.030769	33.79216
gene-MA16_Dca026433	21.229853	24.368912	21.313053	0.345687	0.240142	0.345883	0.414432	0.628763	0.338862
gene-MA16_Dca005538	1.183661	2.622382	1.680104	2.396956	3.2967	3.241475	4.192588	3.835451	6.295942
gene-MA16_Dca006943	19.229345	19.340231	17.509745	44.382553	44.320606	41.980556	51.218513	54.117332	45.349579
gene-MA16_Dca006946	192.052063	195.179199	196.091614	95.668602	90.646713	93.23745	89.176559	89.026215	88.370605
gene-MA16_Dca003277	27.409414	25.253349	24.401588	12.735875	9.786225	10.872261	10.01022	8.655623	9.946672
gene-MA16_Dca003279	12.915225	11.960179	12.246538	1.68463	1.62358	0.985188	1.592876	1.676215	0.962199
gene-MA16_Dca028747	11.146855	8.665486	9.142238	2.170438	4.902144	2.697008	0.500076	0.607015	0.757766
Dendrobium_catenatum_newGene_2590	0.402324	0.408651	0.32187	0.691554	0.290167	0.664325	2.490745	1.3684	2.598766
gene-MA16_Dca025072	115.547546	115.642532	119.713806	12.452743	9.30337	11.099873	11.473103	9.915936	8.74637
gene-MA16_Dca012890	2.981935	3.314546	2.197355	3.623153	3.117412	2.751219	6.919835	5.652306	7.583831
gene-MA16_Dca012896	2.278239	1.791149	2.066976	1.035395	0.658529	0.709583	0.669835	0.685112	0.60308

gene-MA16_Dca023701	1.992692	2.410145	2.31296	3.095685	4.856178	5.953937	10.485729	8.535483	8.926805
gene-MA16_Dca024302	6.794992	6.460185	3.530718	15.108825	15.988596	17.446787	17.508183	17.063162	19.35672
gene-MA16_Dca000203	444.605377	450.116882	429.604431	88.739456	83.238335	127.988197	87.527916	83.182182	82.73777
gene-MA16_Dca012070	11.466642	10.08892	12.006008	27.109543	26.033318	26.401907	54.198288	54.344784	56.428223
gene-MA16_Dca017295	86.863213	89.221245	87.225418	123.746056	123.479492	124.357346	224.944931	213.192825	207.449554
gene-MA16_Dca017290	18.551203	18.791504	18.831844	0	0	0	0.204277	0.16032	0.035772
gene-MA16_Dca018112	12.47988	9.964536	11.750431	17.835537	26.061115	23.938564	49.355301	54.145618	59.921505
gene-MA16_Dca018113	1.217368	3.621074	2.019601	4.661559	4.551566	3.396158	10.782245	8.04344	7.745898
gene-MA16_Dca018110	24.187977	23.220682	23.286909	9.041375	11.659226	11.24631	8.308781	8.749546	10.687012
gene-MA16_Dca025536	10.676235	8.65563	7.863172	0.917931	1.667671	0.581559	0.93359	0.488798	0.777152
gene-MA16_Dca017831	476.904907	476.345093	482.712646	43.045792	40.553329	45.101124	71.36409	65.943199	67.634178
gene-MA16_Dca014506	5.74625	5.69402	5.817021	3.433157	4.705203	5.022882	2.581152	2.849979	3.036649
gene-MA16_Dca014503	0.525679	0.689897	0.889201	7.733397	5.135077	6.279701	6.795111	7.366501	5.937346
gene-MA16_Dca019799	91.985832	93.380493	89.264732	360.530701	340.35141	354.138	590.960938	598.178223	566.927063
gene-MA16_Dca015473	3.756147	3.962394	3.950009	0.664526	0.634151	0.360804	1.207709	1.479329	1.507523
gene-MA16_Dca004971	3.549254	2.796369	4.44641	0.189858	0.082071	0.256349	0.201372	0.246867	0.375026
gene-MA16_Dca004973	325.516724	326.212708	317.24234	19.876497	17.812922	20.015177	54.903366	48.448635	52.167141
gene-MA16_Dca004975	61.257103	62.45536	58.906628	20.517982	17.75985	22.048454	12.033641	10.36933	12.434482
gene-MA16_Dca004974	50.381699	49.274239	49.838245	75.752029	67.199913	69.686623	174.666	159.491058	168.43718
gene-MA16_Dca004977	59.270859	63.231834	59.806274	30.948132	25.323521	26.019258	29.780972	30.858484	34.405647
gene-MA16_Dca020051	47.481678	48.252827	45.531033	30.395004	30.33563	30.695009	22.707743	24.435875	24.597862
gene-MA16_Dca007407	3.458117	2.98144	3.108327	0.269717	0.706016	0.354552	1.203052	0.747111	0.531628
gene-MA16_Dca001802	4.198064	3.684927	4.835572	3.357087	1.761109	2.683151	9.712961	7.636604	8.424259
gene-MA16_Dca001807	102.607498	102.608818	106.293877	58.465549	65.570877	63.509636	25.48423	22.256514	22.188124
gene-MA16_Dca005312	37.28714	40.465294	38.182648	58.255623	55.354008	58.606972	25.774593	27.811722	23.063894
gene-MA16_Dca005313	7.883953	7.802429	8.25691	19.978582	20.002728	20.500074	15.337366	14.125203	12.774811
Dendrobium_catenatum_newGene_15051	2.549862	1.924777	3.52145	6.713077	5.850617	5.929298	4.354836	5.007151	4.381299
gene-MA16_Dca008536	38.80389	41.928028	36.38039	14.756033	16.233595	15.442675	7.013827	6.514999	7.795242

Dendrobium_catenatum_newGene_3954	57.184174	54.571629	56.699619	26.597635	31.24416	28.370783	26.58674	27.701801	27.063673
gene-MA16_Dca004193	35.145142	38.162445	37.230556	28.874159	29.576956	30.678881	15.849954	17.204823	18.827951
gene-MA16_Dca001117	3.48669	4.132044	3.550123	1.656687	1.163121	2.220675	1.517471	1.728876	1.767978
gene-MA16_Dca011749	5.584105	4.002286	5.641086	2.362138	2.300739	2.587996	4.477915	7.845669	5.207508
gene-MA16_Dca010429	7.537359	7.47102	5.923825	11.726832	10.299871	8.522991	4.027126	3.487902	3.290784
gene-MA16_Dca000994	20.180767	23.641478	19.796175	0.935789	0.722967	1.621625	2.272556	3.47267	2.898054
gene-MA16_Dca000992	332.379364	317.795319	344.264099	168.536179	174.422729	170.735153	178.026581	171.365875	166.649658
gene-MA16_Dca000999	2.752895	2.590455	2.611918	0.226981	0.398777	0.314272	0.499343	0.660584	0.215502
gene-MA16_Dca002593	0.638659	0.740624	0.775878	0	0	0.046316	0.071243	0	0
gene-MA16_Dca010901	35.539627	43.160843	43.169529	69.217064	71.54657	81.769676	91.574486	83.609535	84.861267
gene-MA16_Dca009615	56.543873	60.72224	64.197975	16.200089	19.888395	22.286715	17.384338	19.685122	18.684355
gene-MA16_Dca012465	0.20441	0.217014	0.43493	1.033381	0.953555	1.280627	1.105028	0.860281	0.942994
gene-MA16_Dca026204	73.153557	75.533646	78.060349	40.019009	41.80809	43.530396	28.735676	31.261438	33.360645
gene-MA16_Dca026209	16.205555	17.488482	17.311651	13.946087	12.48582	14.003164	9.707875	8.706699	7.819087
gene-MA16_Dca023783	44.081791	40.760368	42.371662	46.99622	45.255127	46.972042	113.269897	107.13826	107.829819
gene-MA16_Dca015278	41.416637	43.06163	40.459789	0.223222	0.113707	0.282443	0.119795	0.063325	0.206323
gene-MA16_Dca015270	1.987147	0.879451	1.179998	1.117242	2.896012	1.933855	2.376648	4.131537	3.607376
Dendrobium_catenatum_newGene_4276	1.204601	1.606537	1.569526	1.441025	0.774024	1.433116	0.437994	0.555221	0.485381
gene-MA16_Dca021520	4.842404	4.425845	4.836159	7.531145	8.007809	7.522459	3.416222	2.291809	3.02843
gene-MA16_Dca020695	37.198059	38.085224	38.5214	17.238766	16.26527	15.955667	19.225948	21.839405	23.082596
gene-MA16_Dca020834	0.364183	0.230467	0.369366	1.81162	1.827917	1.555724	1.87982	1.288668	1.58927
gene-MA16_Dca020831	5.240064	5.224924	4.673187	1.733908	1.785116	2.245873	1.537987	2.036489	1.581677
Dendrobium_catenatum_newGene_11809	0.796561	0.567203	0.712687	6.302279	4.715934	7.447585	5.257902	6.216623	5.331385
Dendrobium_catenatum_newGene_11806	1.699351	0.644394	1.124803	0.889028	1.460562	2.069565	2.580076	3.150478	4.318963
gene-MA16_Dca011220	2.791552	1.990701	2.830328	6.795196	6.2581	6.549649	4.898063	6.4556	6.089274
gene-MA16_Dca011228	1.728768	1.134887	1.812167	0.44411	0.733812	0.382313	1.66946	1.652066	2.232901
gene-MA16_Dca022999	6.439763	4.483977	7.15122	12.75229	12.774145	11.810342	16.749416	16.176403	15.510654
gene-MA16_Dca018094	51.44294	49.903111	52.376949	25.873791	26.063662	24.29184	29.179945	28.323048	25.03105

Dendrobium_catenatum_newGene_873	0.120907	0.256058	0.303719	0.752592	0.412573	0.736205	0.254362	1.116527	0.735591
gene-MA16_Dca019059	0.33972	0.472046	0.176416	0.489494	0.147808	0.157232	1.086995	0.837624	0.873952
gene-MA16_Dca005907	3.434034	5.495463	5.63221	0.784299	0.320185	1.008863	1.390726	2.93758	2.809344
gene-MA16_Dca005905	3.557344	3.874128	4.318974	0.333499	0.197087	0.305277	0.450772	0.493802	0.373937
gene-MA16_Dca005908	244.37999	234.781891	246.751709	82.466049	80.493881	82.405594	106.757957	94.774559	94.630554
Dendrobium_catenatum_newGene_1762	228.693832	226.892548	233.846741	1384.44812	1545.94458	1358.89893	1420.026	1509.39941	1431.25061
gene-MA16_Dca003993	18.962807	22.245325	30.157618	10.948866	13.125091	13.881139	8.194033	7.34089	12.670508
gene-MA16_Dca007679	23.054317	22.54018	23.798141	11.922916	9.842171	10.027537	17.431154	13.591793	16.382137
gene-MA16_Dca009687	9.229332	9.652055	9.063328	3.81276	3.32192	4.00406	8.801391	8.908047	8.722298
gene-MA16_Dca005298	6.851707	5.727576	5.187646	6.089162	5.88588	6.245512	16.961683	13.373943	13.768187
gene-MA16_Dca005291	12.82273	8.947485	9.245209	5.439885	7.348076	5.654669	5.107436	3.855772	2.533235
gene-MA16_Dca009897	96.388603	98.412109	95.1828	36.716743	34.743557	36.411495	60.722286	60.980492	60.641354
Dendrobium_catenatum_newGene_4077	1.407274	1.266683	1.484838	0.424727	0.595307	0.395855	1.082979	1.41652	0.981317
Dendrobium_catenatum_newGene_4076	2.52558	1.834653	1.690306	0.690013	1.054575	0.780152	1.123238	1.014328	1.296649
gene-MA16_Dca012203	7.83289	5.621172	6.334604	3.323291	3.400608	3.610308	1.80921	2.629399	2.897621
gene-MA16_Dca020905	199.135559	205.448669	198.339478	13.106388	13.058828	13.172132	7.164276	6.37285	7.501322
gene-MA16_Dca020902	82.171379	87.154732	83.139267	5.961537	4.520115	5.984194	3.06732	4.133513	3.579168
gene-MA16_Dca002389	1.396358	1.776722	1.250121	3.045506	2.789037	1.577205	5.46868	5.669382	4.834486
gene-MA16_Dca023597	1.182392	1.356164	1.414962	1.924214	2.115997	1.676787	3.47021	2.65638	2.532968
gene-MA16_Dca009408	0.26369	0.409888	0.699939	1.571324	1.28518	1.534457	0.808751	0.459768	1.241445
gene-MA16_Dca012789	3.278651	3.86694	3.366059	0.335079	0.060246	0.38695	0.03624	0.026855	0
gene-MA16_Dca011847	1.534897	1.479386	1.008276	1.329348	1.075076	1.396247	0.805904	0.763139	0.383796
gene-MA16_Dca009534	0.24472	0.415281	0.184672	0.926288	0.423606	0.617004	1.317714	1.06233	1.175863
gene-MA16_Dca014317	0.523419	0.693148	0.529518	1.282497	1.153512	0.867319	3.346563	2.251692	3.793056
gene-MA16_Dca025141	38.91769	38.337616	36.286591	10.288246	11.2674	12.319702	10.31268	7.848943	9.646969
gene-MA16_Dca026746	162.341873	160.30899	148.294052	57.253319	54.384872	59.055779	25.182978	26.676315	25.262657
gene-MA16_Dca008486	0.637977	0.924721	0.865527	0.963735	1.215771	1.107247	2.733716	2.022106	2.408416
gene-MA16_Dca010150	123.492783	133.203812	129.416229	63.369682	66.314888	64.070663	72.65609	71.805046	73.641212

gene-MA16_Dca018132	34.541122	37.576065	38.516861	4.484228	4.573651	5.080448	8.732142	9.580895	9.078178
gene-MA16_Dca007528	0.566788	0.189183	0.314647	0.071471	0.63078	0.389849	1.083795	1.060003	1.192327
gene-MA16_Dca001380	19.848017	16.8736	14.091794	11.193582	10.913857	8.355014	8.300249	8.31846	7.424682
gene-MA16_Dca025805	1.636409	0.647546	1.26114	1.635985	2.786279	1.390817	3.83532	3.775218	4.541493
gene-MA16_Dca017500	3.54331	3.161712	3.204585	0.07389	1.202651	1.142507	0.846053	0	1.08332
gene-MA16_Dca017505	121.210762	133.104675	131.572693	39.943104	33.296017	38.513363	138.901596	137.127563	129.904648
gene-MA16_Dca004262	5.109204	4.502205	4.064923	12.376715	11.019709	10.11609	17.703568	18.402079	22.140223
gene-MA16_Dca001588	38.782745	38.240196	37.673534	70.360214	65.918678	64.105713	85.859421	87.372047	87.139748
gene-MA16_Dca004841	50.521896	55.568027	55.986378	58.963589	63.238522	59.471035	119.340691	123.785469	122.597923
gene-MA16_Dca003810	7.663182	4.208913	5.054131	6.962824	7.371436	5.356355	0.156584	0.204015	0.201132
gene-MA16_Dca003817	3.03793	3.737605	2.926913	0.806625	1.746229	1.518278	1.539822	1.490661	0.996745
gene-MA16_Dca003816	4.116508	3.804105	3.730619	2.576648	1.871329	2.129962	1.835001	1.0571	1.486474
gene-MA16_Dca002144	23.350712	24.034286	22.405605	9.073048	7.300001	7.042656	9.76932	9.60772	9.806161
Dendrobium_catenatum_newGene_1268	0.287835	0.494742	0.476594	0.618385	0.71864	1.319727	1.137637	1.194109	0.72511
gene-MA16_Dca009178	26.384914	30.425726	31.215256	2.76619	2.443655	1.97187	1.107003	0.887042	1.443215
gene-MA16_Dca012812	1.35876	1.337845	1.204553	3.20894	2.411659	3.476696	3.299906	3.083445	2.165865
gene-MA16_Dca002858	13.405492	12.962984	12.470222	7.241603	4.970938	5.465943	5.779647	8.132357	5.259822
gene-MA16_Dca002857	13.57014	13.949471	14.108797	8.430677	10.646891	10.161764	28.654934	27.382122	27.52438
gene-MA16_Dca002851	0.937093	1.123724	1.628317	1.655267	2.599268	2.296502	4.76574	3.044409	2.065477
gene-MA16_Dca019903	34.472099	34.841156	34.931934	16.718998	15.674596	15.241644	19.693367	20.540672	22.468346
gene-MA16_Dca008723	3.156013	3.083183	2.970277	4.767877	3.364523	3.313674	11.275837	14.778221	16.327461
gene-MA16_Dca000178	2.114613	1.388392	1.868131	1.029356	0.455274	0.884802	0.307335	0.313133	0.257169
gene-MA16_Dca000176	1.904459	1.543078	2.547403	0.616511	0.912194	0.98565	3.351887	3.561854	3.861608
gene-MA16_Dca011671	3.528754	4.069086	3.748929	13.869226	12.295403	13.065602	19.532658	21.539778	21.690187
gene-MA16_Dca011673	5.418987	2.938429	5.451494	4.822255	5.127634	4.07618	10.567763	8.810976	12.557177
gene-MA16_Dca011675	29.580921	26.402945	32.11692	45.867203	38.802128	41.44762	64.20929	67.122086	70.750732
Dendrobium_catenatum_newGene_10529	0.405034	0.321486	0.527756	1.10416	1.803993	1.202915	0.631235	1.273652	0.433846
gene-MA16_Dca000827	0	0.648461	0	1.688363	1.548445	1.242383	6.153022	4.620965	4.123254

gene-MA16_Dca003736	45.493847	40.649944	39.737705	128.886444	122.929955	127.692039	224.416626	220.664276	219.083145
gene-MA16_Dca003730	1.199536	1.429245	1.525748	1.636635	0.627694	1.025425	1.003875	0.436676	0.410535
gene-MA16_Dca018021	4.725331	4.640188	5.303474	9.649351	10.612437	11.560427	20.5804	20.261568	21.054501
gene-MA16_Dca003739	11.089277	9.602671	10.150946	5.404155	4.788277	4.595919	5.348481	4.497483	4.322711
gene-MA16_Dca013740	6.170246	4.791653	4.824624	2.610865	4.222684	2.849857	2.692451	1.876261	1.832306
gene-MA16_Dca013741	322.60849	305.866211	317.062103	71.409859	72.324272	70.89698	89.229149	80.418114	81.500656
gene-MA16_Dca013744	3.207603	3.418629	3.28349	4.693892	5.353205	5.438876	9.964341	7.778481	9.985165
gene-MA16_Dca027970	3.265272	2.419808	4.097239	2.161175	1.21698	1.152081	0.134406	0.707181	2.006699
gene-MA16_Dca014430	9.480502	8.216251	9.677935	17.57501	14.36216	15.695577	22.802275	22.306494	23.145819
gene-MA16_Dca014433	0.482979	1.409915	0.778214	2.595537	2.205524	1.953261	5.187667	4.897182	4.805883
gene-MA16_Dca009234	87.986153	94.628738	88.213516	43.861687	40.023613	45.141457	58.553257	52.459473	59.501293
Dendrobium_catenatum_newGene_10898	0.833391	0.579359	0.193669	1.213819	1.410519	1.05468	2.013533	1.417548	1.97282
gene-MA16_Dca026597	5.151175	5.51489	4.306942	9.091811	7.858396	6.564418	13.457534	11.306449	9.995874
gene-MA16_Dca014290	2.257493	2.823519	2.858272	1.569824	1.473908	1.648827	3.862411	3.865314	3.566104
gene-MA16_Dca010243	0.832135	0.393519	0.427799	0	0.40765	0	1.582744	1.669779	1.636229
gene-MA16_Dca010245	1.124467	0.502666	0.720562	2.168022	2.914983	1.802439	3.603544	2.084584	2.75819
gene-MA16_Dca001198	5.387535	5.233377	6.031175	7.195702	7.482189	6.416814	12.630483	12.870937	12.119968
gene-MA16_Dca001191	11.001691	11.504174	11.878421	7.119141	7.257308	7.249707	4.996766	5.033666	4.4895
gene-MA16_Dca001195	3.228915	3.221457	3.116762	1.490223	1.019366	1.282725	2.450125	2.292809	3.074592
Dendrobium_catenatum_newGene_7160	0.208833	0.419389	0.168724	0.529642	0.868944	0.576087	0.630174	0.845633	1.169028
Dendrobium_catenatum_newGene_8396	3.205481	1.414587	1.210746	0.501804	0.347903	0.795481	0.096029	0.26498	0.067868
Dendrobium_catenatum_newGene_8644	148.384827	150.246857	117.817871	48.952148	80.186378	49.643356	59.43573	60.702629	62.139484
gene-MA16_Dca003139	35.624451	35.05241	40.956833	7.141714	7.064649	8.955979	9.76836	8.997451	9.031629
gene-MA16_Dca016970	2.04388	2.607274	2.218185	4.938039	5.285257	5.641886	21.365049	19.108671	20.912987
gene-MA16_Dca008928	20.385996	20.328688	18.753777	4.756475	4.097207	3.548667	3.900979	4.645612	4.036423
gene-MA16_Dca008929	0.863914	0.916307	0.918366	4.690495	2.951328	3.456928	4.974807	6.689516	5.341111
Dendrobium_catenatum_newGene_4764	0.32668	0.205102	0.334933	0.611136	1.185673	0.684276	0.937753	0.918747	0.836486
gene-MA16_Dca015945	131.023056	117.99427	120.07972	3.525844	3.082151	3.054512	2.48299	1.757909	2.343966

gene-MA16_Dca010718	6.32859	7.469532	5.361936	3.630928	2.519975	2.518369	8.798264	6.500103	7.703601
gene-MA16_Dca020619	17.012651	18.344355	19.584196	10.747845	10.612284	12.883975	7.779944	6.970304	8.89502
gene-MA16_Dca000325	64.660538	69.523766	67.433975	30.433451	24.90423	25.228313	45.910812	42.740723	44.948895
gene-MA16_Dca000322	3.099281	1.892821	3.77385	0.6179	0.676649	1.120789	0.076696	0.174817	0.386831
gene-MA16_Dca027345	5.810571	4.933459	4.616296	2.626244	3.228888	1.219149	4.543997	3.597007	3.899681
gene-MA16_Dca027346	8.216586	8.809663	7.367089	6.245079	5.266497	7.707051	15.823993	13.466115	14.393227
gene-MA16_Dca029030	6.846624	6.539556	6.943529	0.748481	1.686962	2.348029	11.005729	11.852374	9.618883
gene-MA16_Dca003676	31.214853	29.072315	31.249245	54.167934	49.322662	50.148674	91.829712	93.392525	95.356232
gene-MA16_Dca016712	104.404175	99.26268	101.766701	83.650406	84.334557	78.323212	49.410538	41.411484	40.647377
gene-MA16_Dca012624	7.045412	5.383218	6.9211	0.418133	1.192178	2.051327	1.797055	1.456763	2.24617
gene-MA16_Dca028348	12.135682	13.535398	10.51167	1.018391	1.922259	3.250235	1.661101	1.986698	1.328854
gene-MA16_Dca017390	25.447138	24.451721	22.485434	9.05353	8.407656	7.586411	7.717033	7.37801	7.970676
gene-MA16_Dca017395	3.997941	3.408921	3.570825	8.599263	6.668052	7.429328	7.599591	10.068824	7.992733
gene-MA16_Dca017394	8.32888	6.462105	7.359288	20.920099	19.460951	22.959747	21.341787	22.162495	21.3999
gene-MA16_Dca026123	18.846422	20.307732	21.344898	2.408726	0.720073	0.849229	0.171132	1.162621	0.401813
gene-MA16_Dca018019	4.738999	7.008348	8.031089	10.876	11.177646	11.676904	19.575844	19.422701	13.631617
gene-MA16_Dca017971	0.305982	0.276541	0.363594	0.227216	0.569462	0.147953	1.144185	0.744499	0.595944
gene-MA16_Dca018905	30.513685	34.577019	33.676605	18.964041	17.081062	20.247566	14.484152	15.75386	15.863931
gene-MA16_Dca015535	352.533264	341.185303	356.121674	122.533447	115.873512	113.223953	139.391388	143.854172	124.260109
gene-MA16_Dca015534	2.096042	2.203696	2.082294	0.03789	0	0	0	0	0
gene-MA16_Dca015533	5.394315	4.927505	6.049578	0	0	0	0	0.114075	0
gene-MA16_Dca021201	3.076962	2.945445	3.084616	1.666197	1.309953	1.314323	0.644379	0.941493	0.688603
gene-MA16_Dca021208	80.431305	82.35585	88.322495	31.967335	31.350578	32.424347	22.221861	22.812832	24.245142
Dendrobium_catenatum_newGene_11261	4.564106	2.357893	3.704491	0.229294	0.27325	0.300228	0.54013	0.374959	0.12256
gene-MA16_Dca007369	14.458781	14.414382	14.436618	2.574628	1.889281	2.183461	2.895221	3.450098	3.879698
gene-MA16_Dca007368	13.344762	13.877922	13.841506	2.539534	2.393403	2.413836	3.481222	4.5954	2.789878
gene-MA16_Dca007367	9.258919	9.890854	9.627669	32.705482	34.485615	33.708828	34.096359	32.466125	31.166882
gene-MA16_Dca008271	4.793588	5.050633	5.327013	1.577529	2.025351	1.990406	2.409101	2.935285	2.121573

gene-MA16_Dca006547	10.360149	11.85709	7.5361	0.403307	0.341499	0.292113	0.587809	0.773218	0.76284
gene-MA16_Dca001015	5.657787	4.399716	5.577767	12.832031	11.089488	10.452188	21.330822	18.538038	18.389868
gene-MA16_Dca001019	9.757985	11.303382	9.856298	6.026973	5.715687	6.209541	4.941745	4.819282	4.721509
gene-MA16_Dca011026	3.685815	2.998878	3.054455	5.544541	3.481312	3.822616	9.38907	7.687137	7.664931
gene-MA16_Dca000653	33.989563	28.900131	27.458866	15.065741	15.661525	13.61639	13.933642	13.768558	10.236189
gene-MA16_Dca000659	615.271484	621.144958	580.334595	124.893013	123.945488	118.289589	221.734421	200.645065	212.976196
gene-MA16_Dca002650	169.237549	155.863937	165.64769	307.151031	301.415619	301.793762	495.610352	453.213409	442.775635
gene-MA16_Dca012014	0.045621	0.343997	0.077941	0.582207	0.128146	0.13579	2.327135	3.876864	2.128038
gene-MA16_Dca003303	1.367651	1.407707	1.388437	2.586479	2.989363	3.878683	2.297046	2.671506	2.508888
gene-MA16_Dca012703	23.063492	24.176373	24.425625	8.145986	8.695085	7.701168	6.653418	6.034676	6.996432
gene-MA16_Dca026327	16.118011	14.744812	15.057422	6.65134	5.838909	8.50299	14.22698	12.266062	17.350945
Dendrobium_catenatum_newGene_2029	12.15344	11.554288	10.374499	39.342151	38.299461	40.819811	42.570798	42.34799	42.592954
gene-MA16_Dca009489	0.585673	1.348468	0.669795	4.528153	5.268094	5.661025	6.816243	6.638508	7.1879
gene-MA16_Dca009484	0.695132	0.44949	0.564757	1.012607	0.776266	1.188281	1.637016	0.825696	1.667292
gene-MA16_Dca009482	67.684219	63.842049	65.829865	62.133083	63.330738	65.852425	184.263062	172.639359	168.840469
gene-MA16_Dca026497	1071.50806	1081.40454	1080.63586	303.338104	268.67865	279.487793	365.737335	334.089844	349.331818
gene-MA16_Dca026491	2.569377	3.059363	3.559672	0.702226	0.825702	0.551134	0.648125	1.066268	0.53922
gene-MA16_Dca025678	1.825051	3.343763	2.529636	0	0.090414	0	0.375494	0	0.134305
Dendrobium_catenatum_newGene_327	5.059599	4.227137	5.405555	7.088746	6.508692	6.589643	13.408623	11.014321	9.271508
Dendrobium_catenatum_newGene_4151	12.929427	12.158155	10.896251	16.082104	16.841047	17.315908	31.113565	22.343849	28.910482
gene-MA16_Dca019618	29.919146	31.391403	29.434147	64.306557	56.58313	61.545052	157.473312	138.646088	145.440887
gene-MA16_Dca019614	259.353668	266.690247	250.226364	113.784538	105.753426	114.508759	151.061035	151.363724	161.631836
gene-MA16_Dca010035	0.623709	0.101811	0.753298	3.222747	1.038657	3.560439	7.767034	4.458792	3.915362
gene-MA16_Dca024235	9.916025	10.410247	8.864775	3.185339	3.075744	4.764778	1.972291	2.851273	4.187437
gene-MA16_Dca024236	22.346994	23.968002	25.026506	10.025735	10.149684	9.10586	9.634825	9.411285	10.311101
gene-MA16_Dca020425	32.313541	34.386417	30.941959	18.090982	19.808878	16.592432	10.591442	12.513752	10.020147
gene-MA16_Dca020426	687.380554	689.088562	674.485413	778.037048	719.960205	775.846619	238.851639	243.048508	236.671082
gene-MA16_Dca011342	21.018745	17.592369	23.04895	11.36067	7.596232	11.263497	10.295748	11.458825	10.965545

gene-MA16_Dca027046	48.51786	50.314022	46.735485	9.915061	9.586271	8.304991	11.489336	10.505591	10.248653
gene-MA16_Dca027044	1.125665	1.672947	1.563057	1.629271	1.230736	1.713923	0.69246	0.519625	0.491304
gene-MA16_Dca027043	16.552317	17.69219	20.93152	17.722132	23.271816	22.076836	7.442264	8.493609	6.969457
gene-MA16_Dca026694	21.832481	23.19396	21.310783	0.722423	0.667619	0.591542	0.943553	1.218885	0.626248
gene-MA16_Dca023309	0.219013	0.495726	0.989912	1.700662	0.516564	0.766269	4.602773	2.741257	2.695706
gene-MA16_Dca025889	25.437778	22.348444	25.523306	5.581201	5.031095	4.871472	8.909997	4.62996	7.946947
gene-MA16_Dca019152	41.402676	36.199516	37.863823	16.730164	13.270836	14.448936	11.955547	13.685743	13.017419
Dendrobium_catenatum_newGene_1150	1.603569	1.554582	0.924479	1.271491	2.403043	2.056978	4.767098	4.420094	2.321902
gene-MA16_Dca014228	0.549614	0.551576	0.2044	1.192281	0.746748	1.288692	3.095668	2.151507	1.945286
gene-MA16_Dca014229	1.63091	1.779229	0.917167	0.229348	0.224231	0.383163	1.380388	1.340627	1.69564
gene-MA16_Dca018761	6.080248	7.82231	4.731136	0.663415	0.285872	0.185543	0.754816	0	0.870951
gene-MA16_Dca007539	2.446423	2.489681	1.565281	0.526841	0.965211	1.206515	1.022271	1.221859	1.174962
gene-MA16_Dca007531	89.025398	88.880699	81.955788	30.913618	29.901392	28.535946	9.551126	10.176535	8.730275
gene-MA16_Dca007533	62.224392	69.10302	62.714222	16.968468	14.80374	16.247623	12.102451	12.782186	11.677429
gene-MA16_Dca000256	0.748324	0.728802	1.111777	1.62405	1.258338	1.563964	2.263253	2.384798	2.009393
gene-MA16_Dca010235	4.800573	5.090696	3.864703	1.651219	3.75376	2.821055	1.61616	2.129554	1.171413
gene-MA16_Dca010234	3.526282	2.859388	3.297202	2.09894	1.574776	2.265321	1.311226	0.86708	1.424524
gene-MA16_Dca010580	0.210762	0.192497	0.286337	0.431617	0.372712	0.565825	0.574091	0.553124	0.453
gene-MA16_Dca003406	7.807133	8.177645	7.928373	29.624901	34.336651	31.851175	51.716846	54.143936	50.064617
gene-MA16_Dca014342	7.880379	8.292576	7.997176	3.148702	2.912121	4.047244	3.911541	4.313766	4.230934
gene-MA16_Dca016322	1.133765	0.482808	1.190957	0.54877	0.621125	0.63945	1.855649	1.539052	1.71119
Dendrobium_catenatum_newGene_6403	1.501068	1.582771	1.659026	2.010617	3.226685	2.334083	3.81591	3.690814	3.789291
Dendrobium_catenatum_newGene_6400	0.657878	0.778482	0.611124	0.699233	0.507558	1.047948	1.468219	1.21291	2.749024
gene-MA16_Dca022813	14.533894	15.485093	14.753707	8.172957	7.636961	8.113008	5.405594	5.469626	5.948597
gene-MA16_Dca022811	12.936227	10.713061	11.559518	25.049089	29.960205	24.957544	39.453022	39.763142	42.161961
gene-MA16_Dca009728	12.728527	15.011605	15.5116	13.292643	11.38676	14.114109	6.157997	6.609932	9.197234
Dendrobium_catenatum_newGene_16416	2.936407	3.846785	4.114913	2.77885	1.751691	1.805928	1.312644	1.84754	1.336892
gene-MA16_Dca026517	3.177794	3.362977	3.537237	5.603275	3.504178	4.149095	0.830972	0.473268	0.706986

gene-MA16_Dca025555	5.998327	6.401714	7.403864	15.825158	19.74292	16.955519	35.419159	32.609486	38.277412
gene-MA16_Dca017749	145.03334	143.441223	150.979019	61.392319	61.887897	65.841721	46.021317	44.376152	47.217968
gene-MA16_Dca012513	100.532608	96.457306	109.692818	18.4849	18.847626	14.859425	28.882839	24.696045	25.613153
gene-MA16_Dca024918	59.061195	55.973305	53.508541	25.682608	23.753809	25.537098	21.988094	22.099501	24.384449
gene-MA16_Dca011961	0.476927	0.31593	0.242169	1.499498	1.337231	0.934237	2.340942	2.003734	4.075485
gene-MA16_Dca014219	617.685486	573.772034	584.662659	115.551414	117.532661	123.200272	106.495407	92.167854	99.036896
gene-MA16_Dca018775	1.383036	1.310953	1.234711	0	0.066959	0.061768	0	0	0
gene-MA16_Dca018770	49.572388	47.947403	45.737621	82.691391	76.646713	64.200478	104.469505	95.550568	106.352753
Dendrobium_catenatum_newGene_288	20.568174	20.383963	22.42078	56.287327	63.550842	62.55439	89.843491	88.97789	85.147934
gene-MA16_Dca019421	16.853594	17.474575	15.791872	11.479984	13.271791	10.785874	4.749173	6.649001	7.737991
gene-MA16_Dca021631	25.145731	26.688196	24.97011	46.996086	42.735256	47.34676	64.963371	68.500427	66.275146
gene-MA16_Dca021635	0.458697	0.633608	0.23742	0.37518	0.312165	0.393458	1.299391	1.637459	1.023723
gene-MA16_Dca021634	0	0	0.070982	0.252701	0	0.07083	0.943747	0.616995	1.337623
gene-MA16_Dca021981	35.609192	31.511456	33.920906	19.888412	16.633177	16.945755	20.138231	17.258902	18.202276
gene-MA16_Dca024008	61.561413	55.791035	60.436012	34.895065	26.322842	26.229719	33.402672	31.55554	27.001101
gene-MA16_Dca020584	2.680422	3.801054	3.137996	1.683019	1.431239	1.363099	2.340496	2.535538	2.866333
gene-MA16_Dca027673	1.902289	1.982035	2.338453	1.884117	3.158669	2.082276	8.257369	6.10145	7.789627
gene-MA16_Dca026982	47.542595	51.186062	45.632328	33.044556	32.44611	32.017712	23.434057	21.893309	24.411716
gene-MA16_Dca023806	11.997796	11.304694	10.441588	5.033288	5.736609	4.057892	5.09068	5.687547	4.142628
gene-MA16_Dca027583	2.425499	1.945606	1.514992	1.020949	1.059624	1.678416	0.57379	0.520629	0.726168
gene-MA16_Dca017260	4.043802	3.691475	2.845272	4.036154	4.460591	5.55387	7.220388	8.570336	7.476413
Dendrobium_catenatum_newGene_5680	0.954948	0.456329	0.547449	0.711507	0.785682	0.733385	1.838073	2.032389	1.888987
Dendrobium_catenatum_newGene_6640	14.841399	15.423709	12.233612	8.591578	8.675344	9.674936	3.772865	2.74064	3.930181
Dendrobium_catenatum_newGene_9191	1.131674	0.616178	0.515145	1.971767	0.7735	1.485881	2.876604	1.92956	1.275355
Dendrobium_catenatum_newGene_9190	2.596544	2.573515	2.805754	3.570017	3.56096	3.033901	7.486042	6.347242	8.279375
Dendrobium_catenatum_newGene_7599	0	0.067301	0	0.215936	0.077849	1.094241	2.234983	2.207882	1.493235
gene-MA16_Dca007703	1.990916	1.684994	1.551409	2.633379	2.414905	2.606436	4.670424	3.663646	5.277182
gene-MA16_Dca002950	2.218186	1.562099	1.017038	0.377668	0.317574	0.403207	0.172653	0.172973	0.258775

gene-MA16_Dca006258	63.832844	58.514366	60.883816	31.330976	31.008156	30.55036	34.04483	35.299217	34.530224
gene-MA16_Dca001704	136.806885	126.757477	130.14798	67.764084	70.741829	70.169762	46.348988	45.036964	43.208748
gene-MA16_Dca001703	78.54348	65.550438	64.948486	59.025837	56.22031	58.430386	32.131035	27.390402	32.817139
gene-MA16_Dca010796	77.848076	79.030647	78.971802	97.274094	93.594177	97.826309	164.040726	172.822922	171.662872
Dendrobium_catenatum_newGene_10201	1.316913	1.349838	1.482817	1.562442	1.537242	1.518998	0.384266	0.44641	0.411288
gene-MA16_Dca016797	40.876263	39.646027	40.017479	51.933857	50.63409	53.488369	89.289978	87.241821	81.263771
gene-MA16_Dca011447	4.618937	4.275408	4.256487	1.503243	1.320599	0.823001	1.030021	0.661169	0.485074
Dendrobium_catenatum_newGene_16332	39.314128	37.824468	35.019255	14.75267	9.770065	13.686039	9.647912	12.713332	14.533962
Dendrobium_catenatum_newGene_11642	159.679474	146.742661	174.181763	148.282928	143.713013	151.101028	53.789043	54.992592	45.711159
gene-MA16_Dca019835	12.738192	14.128524	15.421272	6.195265	7.655777	5.664757	10.983979	8.841543	10.482273
gene-MA16_Dca012492	22.040251	23.772968	19.80447	10.01572	9.428421	8.464051	9.301547	8.616078	8.711066
Dendrobium_catenatum_newGene_10996	14.423358	15.828261	13.356158	6.096716	5.886208	5.803416	6.616868	5.425972	4.264213
gene-MA16_Dca019323	1.814385	1.166944	1.525067	8.002134	7.993552	8.816258	7.837389	7.195901	6.485986
gene-MA16_Dca018982	2.825526	2.72144	2.162763	0.315801	0.685915	0.676341	0.576411	0.125588	0.328836
gene-MA16_Dca021289	2.136397	1.329105	1.419599	2.623295	2.292178	2.770552	5.437661	4.978685	4.712112
Dendrobium_catenatum_newGene_9355	3.298626	4.37094	4.848503	6.381134	4.908823	6.119658	11.457222	8.800247	11.88536
gene-MA16_Dca021863	41.144531	41.386597	42.323898	22.855732	18.278749	20.599449	26.185867	23.927057	27.488016
gene-MA16_Dca021861	4.786395	4.832012	4.421511	1.248078	1.10585	1.47984	3.935427	3.48546	2.848635
gene-MA16_Dca027425	6.85	6.007495	6.626916	2.275964	1.661886	1.551952	1.663486	1.865734	0.930831
Dendrobium_catenatum_newGene_8901	11.082898	8.850189	9.76712	16.985468	17.175428	18.058914	28.794436	30.399612	26.269772
gene-MA16_Dca006019	0.984354	1.22613	1.431368	0.471596	1.014637	0.539265	0.364892	0.231487	0.277469
gene-MA16_Dca004528	12.466298	9.334455	7.631545	1.561852	0.831047	2.022262	3.728755	3.200122	2.715747
gene-MA16_Dca004529	47.657703	45.311646	41.264519	26.350504	28.384901	22.364914	20.64319	20.748623	20.905678
gene-MA16_Dca028652	1.38286	1.457767	1.163011	0.821075	0.729872	0.957473	0.561195	0.544588	0.26597
gene-MA16_Dca001540	0.758678	0.744604	0.995473	1.832865	2.512123	1.554993	6.120012	6.34907	4.102226
gene-MA16_Dca007878	38.737701	42.377121	42.872433	17.49383	15.98334	16.533752	15.228431	14.828918	11.230588
gene-MA16_Dca007873	36.886806	38.330719	32.58569	13.411875	12.128808	11.329366	9.220632	9.249921	9.120099
gene-MA16_Dca001095	4.327103	2.336971	2.248345	1.003565	0.416593	0.494481	3.094133	2.931809	3.765301

gene-MA16_Dca010882	1.458495	1.681645	1.611043	0.252666	0.528711	0.619744	1.163122	0.554717	0.970154
Dendrobium_catenatum_newGene_8120	47.631001	42.253422	44.2673	18.164284	18.736546	15.089849	17.711025	15.011872	14.639354
gene-MA16_Dca004062	168.174469	161.970306	170.393707	218.10791	203.95488	213.071976	444.547974	438.583282	436.932159
gene-MA16_Dca026389	5.556557	5.236608	6.173546	1.322229	0.824686	1.190502	0.403249	0.388675	0.307062
gene-MA16_Dca028103	2.278022	1.69093	1.295082	0.257469	0.043661	0.468717	0	0.253327	0.302238
Dendrobium_catenatum_newGene_1061	0.18885	0.132799	0.422085	0.397664	0.467139	0.533821	0.897468	0.751709	1.17966
gene-MA16_Dca009375	29.747988	32.872887	31.13855	82.422096	76.13237	78.925034	123.750603	110.97805	116.779053
gene-MA16_Dca002031	7.797134	8.454844	7.494905	12.635729	11.479417	11.735575	5.639454	5.843348	4.80276
gene-MA16_Dca012960	37.972466	40.491203	34.790058	15.595457	13.885912	13.17466	13.588387	11.947075	10.910263
gene-MA16_Dca008135	8.822217	8.847919	8.121862	19.939125	21.587734	24.645657	27.805735	28.094103	30.745779
gene-MA16_Dca008136	48.075981	52.100746	48.854992	81.681038	72.302757	77.735474	135.843674	133.067474	128.349503
gene-MA16_Dca008139	44.511147	45.36092	44.94939	12.628196	10.843189	12.865411	49.037273	45.984295	45.090679
gene-MA16_Dca019872	1.250061	2.346655	1.402464	1.002382	1.030962	0.989915	3.97681	3.087004	2.651042
gene-MA16_Dca010616	1.769886	1.149206	2.142555	0.883664	0.695308	0.822077	2.162751	1.304625	0.819287
gene-MA16_Dca026612	6.341189	5.177507	6.283495	3.444296	3.681751	2.626465	2.192101	1.237568	1.275904
gene-MA16_Dca016618	46.618538	48.890499	47.394062	20.289936	21.899923	20.782524	26.318565	28.494911	30.144533
gene-MA16_Dca017058	54.887459	49.526218	55.028427	53.404442	49.940472	49.930878	22.275492	23.475805	20.071037
gene-MA16_Dca017057	22.435143	21.211733	20.027506	0.083224	0.155204	0.141384	0.091768	0.180326	0
gene-MA16_Dca018861	133.491272	131.248611	129.549667	108.503098	109.896545	100.050095	53.240276	56.345024	53.516647
gene-MA16_Dca021128	0.832795	1.084708	1.399096	3.261037	3.577452	3.822313	5.429175	4.490921	4.455304
gene-MA16_Dca021120	12.516397	12.346364	11.879917	8.82105	9.31316	8.553665	4.972001	4.948672	4.031755
gene-MA16_Dca021124	65.699265	63.063854	74.72065	103.710663	114.967697	105.838394	156.045532	166.568314	141.82901
gene-MA16_Dca015367	91.84935	94.865372	94.349388	15.04239	15.485087	15.281951	13.816611	16.541025	16.205124
gene-MA16_Dca020299	13.241625	13.288033	12.742691	3.621861	3.939563	3.596467	4.064775	3.139681	5.131758
gene-MA16_Dca020745	42.488857	42.883617	45.2934	24.767923	26.212475	25.572481	23.387627	22.519722	20.151438
gene-MA16_Dca007244	42.087963	45.961132	40.41082	80.293137	80.372017	84.939323	123.355125	121.26136	142.361465
gene-MA16_Dca007245	0	0.058527	0	2.356874	1.14687	2.221394	8.710509	5.925941	4.683914
gene-MA16_Dca024196	3.491404	3.784758	2.793438	2.331094	1.777671	1.374129	1.822584	1.662493	1.437945

gene-MA16_Dca006198	4.474293	2.534245	2.27357	0.199636	0.227869	0.751792	0.614182	0.078026	0.848487
Dendrobium_catenatum_newGene_9841	42.549271	40.724774	44.034467	21.117131	21.18446	19.80078	14.444167	17.388707	17.766447
gene-MA16_Dca011103	3.458203	2.814642	3.304214	7.445465	6.497674	8.053863	5.208909	4.359904	3.996245
Dendrobium_catenatum_newGene_1456	3.071448	1.837946	3.778024	7.423698	6.772999	7.272867	9.197959	10.011584	11.831047
gene-MA16_Dca020991	534.261475	436.088257	610.883789	212.659714	373.184174	208.540024	244.56601	252.122955	305.565308
gene-MA16_Dca028731	12.745929	10.05758	10.797389	0.903363	0.639823	0.404735	0.727016	0.846801	1.574729
gene-MA16_Dca022678	5.303127	4.353638	3.287874	0.714285	0.587105	1.106873	0.716274	0.245082	0.604889
gene-MA16_Dca008812	5.130173	3.825477	4.152697	5.915961	4.879885	6.474218	2.59783	2.207451	2.260233
gene-MA16_Dca008814	6.428249	5.910119	5.9345	0.602727	0.64816	0.786135	0.905356	0.690194	0.782846
gene-MA16_Dca013240	0.463341	0.154267	0.533445	0.458451	0.487391	0.891153	1.18038	1.525138	1.580348
gene-MA16_Dca024080	5.759667	4.613564	4.78068	0.418796	0.169544	0.131902	0.551251	0.396235	0.446497
gene-MA16_Dca024084	180.168594	183.322128	181.41333	68.965767	62.797665	65.849976	46.790745	42.840359	42.690331
gene-MA16_Dca026759	159.417572	135.445206	159.59407	41.613369	103.778313	42.125439	41.090229	53.017422	61.269127
gene-MA16_Dca015834	13.280752	14.557715	12.93444	11.684381	10.834099	10.479733	6.269823	7.221236	7.525002
gene-MA16_Dca029102	2.318522	3.413311	4.474202	2.233521	4.15548	0.643659	8.650068	9.413727	6.918003
gene-MA16_Dca026906	1.40461	1.086387	1.51613	2.466015	2.893827	2.06421	3.700421	3.60121	3.413575
gene-MA16_Dca026901	1.850855	2.870933	1.688186	5.881034	6.856713	4.945889	2.519198	2.066945	2.955343
gene-MA16_Dca023268	894.542603	922.385925	877.468994	132.524414	111.457947	116.959076	148.680038	134.511322	132.297226
gene-MA16_Dca003158	8.110241	6.34755	7.625815	16.052546	12.532814	13.653567	18.432537	16.739698	15.409343
gene-MA16_Dca026019	8.876454	7.769536	7.246148	0.835556	1.198623	0.941503	1.792642	1.607613	1.557613
gene-MA16_Dca010466	34.15176	35.299244	29.979994	7.22471	6.858959	8.09346	4.566831	3.107783	2.168162
gene-MA16_Dca010463	26.211037	27.391211	29.104109	9.252142	8.207567	9.348589	8.35975	8.656161	5.987973
gene-MA16_Dca019297	32.979893	31.367168	32.027061	13.648491	12.11938	14.91708	20.926702	18.712681	20.440716
gene-MA16_Dca014713	127.340317	125.621307	118.935959	80.390259	85.606125	85.186424	54.077442	47.553387	50.984489
gene-MA16_Dca019741	4.392276	3.522442	4.230282	0.128834	0	0	0	0	0.102235
gene-MA16_Dca021379	19.650858	21.923246	24.144514	6.964804	5.639874	6.323209	9.065772	9.177787	8.083861
gene-MA16_Dca021373	14.882792	14.224918	13.46568	12.464123	14.457286	14.149132	6.779222	6.036939	6.678681
gene-MA16_Dca021371	4.52016	4.445192	4.595359	10.299759	8.560363	6.938067	11.898362	10.372509	11.443406

gene-MA16_Dca021377	24.368107	24.187744	20.420156	60.775593	58.745625	64.519646	119.77285	111.287834	111.825409
gene-MA16_Dca015443	27.933605	25.694994	27.794638	8.204865	8.184688	8.550358	8.864871	8.344699	8.653909
gene-MA16_Dca004924	2.887436	3.11894	4.134167	0.322462	0.910006	0.50546	0.43432	0.538843	0.116328
gene-MA16_Dca005329	4.192509	5.550784	3.780799	1.403463	1.558871	1.508293	1.273448	0.723246	0.788463
gene-MA16_Dca007781	1.66924	0.992003	1.470737	2.214831	2.072788	2.659911	4.092905	3.377952	4.747284
gene-MA16_Dca001836	2.907329	3.010866	2.474296	4.144674	4.368387	3.900935	7.028458	7.279661	7.42808
gene-MA16_Dca006431	240.007385	235.782608	220.927338	117.497765	120.097122	126.382408	63.923351	63.26606	66.860962
gene-MA16_Dca008697	54.2463	56.281662	54.661289	22.16617	21.212637	19.402187	22.010618	21.089235	22.011656
gene-MA16_Dca004147	13.279277	16.569391	16.216232	4.529667	5.429538	7.746277	3.948497	4.239231	4.523049
gene-MA16_Dca000494	42.973335	42.315411	38.873619	28.57745	25.754595	24.672653	17.43454	20.256624	18.237589
