# Supplementary material for: RNA sequencing-based exploration of the effects of blue laser irradiation on mRNAs involved in functional metabolites of D. officinales
Source: PeerJ. 2022 Jan 4;10:e12684. doi: 10.7717/peerj.12684 (PMC8740519; doi:10.7717/peerj.12684)
Supplement: Supplemental Information 1 [file peerj-10-12684-s001.zip › Supplemental information/Table S5.docx]

| **Table S5** POD activity of leaves in *D. officinale* under different light treatments | | | | | | | | |  |
| --- | --- | --- | --- | --- | --- | --- | --- | --- | --- |
| Light treatments | Light intensity (µmol·m^-2^·s^-1^) | Photoperiod (h) | POD activity value 1  (U·g ^-1^FW) | POD activity value 2  (U·g ^-1^ FW) | POD activity value 3  (U·g ^-1^ FW) | POD activity value  (U·g ^-1^ FW) | Standard deviation | Duncan (5%) | Duncan (1%) |
| White | 100 | 12 | 850.00 | 760.00 | 670.00 | 760.00 | 90.00 | c | C |
| Blue | 100 | 12 | 1670.00 | 1890.00 | 1580.00 | 1713.33 | 159.48 | b | B |
| Blue Laser | 100 | 12 | 2010.00 | 1710.00 | 1880.00 | 1236.67 | 150.44 | a | A |
