# Supplementary material for: RNA sequencing-based exploration of the effects of blue laser irradiation on mRNAs involved in functional metabolites of D. officinales
Source: PeerJ. 2022 Jan 4;10:e12684. doi: 10.7717/peerj.12684 (PMC8740519; doi:10.7717/peerj.12684)
Supplement: Supplemental Information 1 [file peerj-10-12684-s001.zip › Supplemental information/Table S7.docx]

| **Table S7** PAL activity of leaves in *D. officinale* under different light treatments | | | | | | | | |  |
| --- | --- | --- | --- | --- | --- | --- | --- | --- | --- |
| Light treatments | Light intensity (µmol·m^-2^·s^-1^) | Photoperiod (h) | PAL activity value 1  (U·g ^-1^FW) | PAL activity value 2  (U·g ^-1^ FW) | PAL activity value 3  (U·g ^-1^ FW) | PAL activity value  (U·g ^-1^ FW) | Standard deviation | Duncan (5%) | Duncan (1%) |
| White | 100 | 12 | 42.21 | 52.25 | 56.40 | 50.29 | 7.29 | c | C |
| Blue | 100 | 12 | 184.33 | 168.50 | 166.25 | 173.03 | 9.85 | b | B |
| Blue Laser | 100 | 12 | 187.10 | 177.84 | 183.73 | 182.89 | 4.68 | a | A |
