# Supplementary material for: RNA sequencing-based exploration of the effects of blue laser irradiation on mRNAs involved in functional metabolites of D. officinales
Source: PeerJ. 2022 Jan 4;10:e12684. doi: 10.7717/peerj.12684 (PMC8740519; doi:10.7717/peerj.12684)
Supplement: Supplemental Information 1 [file peerj-10-12684-s001.zip › Supplemental information/Table S8.docx]

| **Table S8** Flavonoid contents of leaves in *D. officinale* under different light treatments | | | | | | | | |  |
| --- | --- | --- | --- | --- | --- | --- | --- | --- | --- |
| Light treatments | Light intensity (µmol·m^-2^·s^-1^) | Photoperiod (h) | Flavonoid contents 1  (mg·g ^-1^DW) | Flavonoid contents 2  (mg·g ^-1^ DW) | Flavonoid contents 3  (mg·g ^-1^ DW) | Average Flavonoid  contents  (mg·g ^-1^ DW) | Standard deviation | Duncan (5%) | Duncan (1%) |
| White | 100 | 12 | 33.93 | 27.88 | 27.88 | 29.90 | 2.85 | c | C |
| Blue | 100 | 12 | 57.20 | 51.62 | 48.82 | 52.55 | 3.48 | b | B |
| Blue Laser | 100 | 12 | 73.96 | 75.82 | 72.56 | 74.11 | 1.34 | a | A |
